# Machine Learning‐Driven Prediction, Preparation, and Evaluation of Functional Nanomedicines Via Drug–Drug Self‐Assembly

**DOI:** 10.1002/advs.202415902

**Published:** 2025-01-10

**Authors:** Chengyuan Zhang, Yuchuan Yuan, Qiong Xia, Junjie Wang, Kangkang Xu, Zhiwei Gong, Jie Lou, Gen Li, Lu Wang, Li Zhou, Zhirui Liu, Kui Luo, Xing Zhou

**Affiliations:** ^1^ Yunnan Key Laboratory of Stem Cell and Regenerative Medicine Kunming Medical University Kunming 650500 China; ^2^ School of Medicine Northwest University Xi'an 710068 China; ^3^ Department of Pharmacy School of Pharmacy and Bioengineering Chongqing University of Technology Chongqing 400054 China; ^4^ Department of Biomedical Engineering School of Engineering China Pharmaceutical University Nanjing 210009 China; ^5^ Department of Pharmacy Xinan Hospital Army Medical University Chongqing 400038 China; ^6^ Department of Radiology and Department of Geriatrics Huaxi MR Research Center (HMRRC) National Clinical Research Center for Geriatrics Frontiers Science Center for Disease‐Related Molecular Network State Key Laboratory of Biotherapy West China Hospital Sichuan University No. 37 Guoxue Alley Chengdu 610041 China

**Keywords:** machine learning, nanomedicine, self‐assembly, small‐molecular drug carrier

## Abstract

Small molecules as nanomedicine carriers offer advantages in drug loading and preparation. Selecting effective small molecules for stable nanomedicines is challenging. This study used artificial intelligence (AI) to screen drug combinations for self‐assembling nanomedicines, employing physiochemical parameters to predict formation via machine learning. Non‐Steroidal Anti‐Inflammatory Drugs (NSAIDs) are identified as effective carriers for antineoplastic drugs, with high drug loading. Nanomedicines, PEG‐coated indomethacin/paclitaxel nanomedicine (PiPTX), and laminarin‐modified indomethacin/paclitaxel nanomedicine (LiDOX), are developed with extended circulation and active targeting functions. Indomethacin/paclitaxel nanomedicine iDOX exhibits pH‐responsive drug release in the tumor microenvironment. These nanomedicines enhance anti‐tumor effects and reduce side effects, offering a rapid approach to clinical nanomedicine development.

## Graphical abstract

Machine learning technology is used to screen small molecule drug combinations for self‐assembling nanomedicines. Utilizing small molecule non‐steroidal anti‐inflammatory drugs as carriers, they effectively transport antineoplastic drugs. Boasting remarkable drug‐loading capabilities, they open avenues for tumor treatment through chemotherapy and immunomodulation combined therapy. These nanomedicines demonstrate vital attributes such as extended systemic circulation, controlled drug release, and precise tumor targeting.

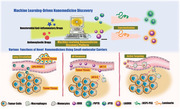

## 1 Introduction

Nanomedicine has emerged as a promising therapeutic modality in preclinical and clinical trials, primarily due to the advantages conferred by its nanoscale dimension of it and its multifunctionality.^[^ ^1^ ^]^ Nanomedicine derived from functional polymers has shown great promise because the capacity of these polymers can accommodate a broad spectrum of therapeutic/diagnostic drugs and the nanomedicine is endowed with multiple functions including controlled release in response to tumor microenvironment cues, targeted accumulation in lesions, and prolonged systemic circulation.^[^ ^2^, ^3^ ^]^ Recently, there has been an increasing interest in developing nanomedicine utilizing small‐molecule carriers, particularly FDA‐approved drugs.^[^ ^3^ ^]^ Small molecule‐based nanomedicine offers several advantages, including a high drug‐loading capacity, straightforward and cost‐effective preparation processes, well‐characterized metabolic pathways, and established safety profiles.^[^ ^3^ ^]^

However, it is very challenging to identify an FDA‐approved small molecule drug combination that can assemble into nanomedicines, especially one that can provide synergistic therapeutic effects. Meanwhile, the drug–drug assembly mechanisms are barely unveiled.^[^ ^3^, ^4^ ^]^ This is where machine learning and other artificial intelligence technologies come into play. With the advent of sophisticated machine learning algorithms and large‐scale computational capabilities, artificial intelligence is poised to significantly advance drug discovery innovations, including in designing optimal drug formulations and drug delivery systems (DDS).^[^ ^5^ ^]^ Furthermore, computational simulations and physical characterization techniques provide valuable insights into drug–drug assembly mechanisms in small molecule‐based nanomedicine.^[^ ^6^ ^]^

Simultaneously, nanomedicines derived from functional polymers are renowned for their capacity to perform multiple functions.^[^ ^2^ ^]^ These attributes are equally vital for small molecule‐derived nanomedicines.^[^ ^7^ ^]^ Achieving such functionalities in small molecule‐based nanomedicines, currently necessitates sophisticated chemical synthesis techniques, posing significant challenges for clinical translation.

Non‐steroidal anti‐inflammatory drugs (NSAIDs) are effective in relieving cancer‐related symptoms, and they have exhibited potent anti‐tumor effects, thus they have been clinically applied to anti‐cancer treatment.^[^ ^8^ ^]^ The salicylic acid moiety within NSAIDs can engage non‐covalent interactions, such as electrostatic interactions, hydrogen bonding, and π–π stacking with antineoplastic drugs, therefore, NSAIDs have great potential as small‐molecular carriers for antineoplastic drugs to prepare small‐molecule based nanomedicine.

**Figure 1:**
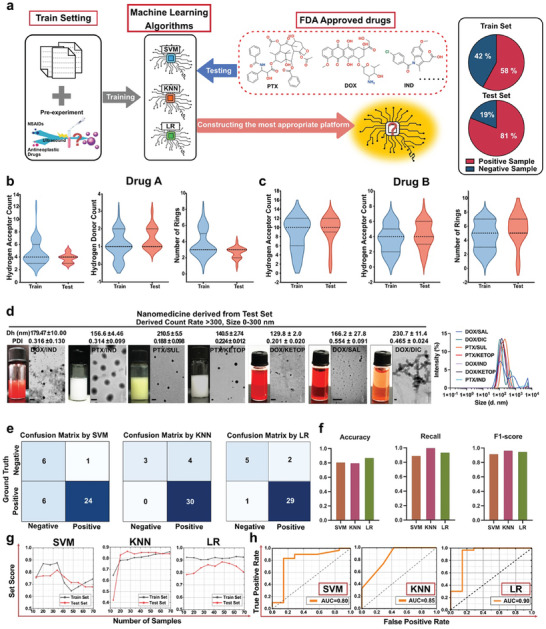
Construction and evaluation of a machine learning platform to predict the formation of nanomedicines using small molecular drugs as carriers. a) Scheme of screening a machine learning model to construct a prediction platform for the formation of nanomedicines using nonsteroidal anti‐inflammatory drugs (NSAIDs) as carriers. The labels for the dataset were negative and positive outcomes: negative for drug combinations that could not self‐assemble into nanomedicines (a derived count rate <300 and a size >300 nm), while positive for drug combinations that could self‐assemble into nanomedicines (a derived count rate >300 and a size <300 nm); b,c) The distribution of features of NSAIDs and antineoplastic drugs in the train set and the test set. NSAIDs were assigned to Drug A, and antineoplastic drugs were assigned to Drug B; d) Photographs and transmission electron microscopy (TEM) images of nanomedicines assembled from small molecule drugs, consistent with the predictions of machine learning models. Scale bars = 200 nm; e) Confusion matrices of different machine learning models; f) Accuracy, Recall, and F1 Score of different machine learning models; g,h) Learning curves, and receiver operating characteristic (ROC) curves of different machine learning models.

In this investigation, we combined FDA‐approved small molecule non‐steroidal anti‐inflammatory drugs (NSAIDs) with chemotherapeutic agents to fabricate nanomedicines. We amalgamated the outcomes with previously documented small molecule carrier nanomedicines^[^ ^3^ ^]^ and successfully devised an AI‐based predictive platform utilizing three machine learning algorithms. This platform proficiently and accurately discerns novel anti‐tumor nanomedicines employing small molecule carriers (**Figure** **1**a).

We elucidated the drug–drug assembly mechanisms by integrating computational simulations with empirical scientific experiments, thereby laying the groundwork for future enhancement of the AI model. Among the nanomedicines forecasted by the AI platform, we exemplified the functionalization of nanomedicines constituted by indomethacin (IND) and prevalent anticancer drugs such as paclitaxel or doxorubicin. Utilizing a facile supramolecular assembly process, we accomplished the functionalization of these nanomedicines, including long‐circulating paclitaxel nanomedicines, tumor microenvironment‐responsive doxorubicin nanomedicines, and monocyte‐targeting doxorubicin nanomedicines. These meticulously engineered nanomedicines exhibited synergistic anti‐tumor efficacy and multifunctionality, demonstrating substantial potential for clinical translation.

## 2 Results and Discussion

### 2.1 Building a Machine Learning Prediction Platform for Nanomedicines using FDA Approved Small Molecular Drugs as Carriers

In our previous study, we successfully used indomethacin (IND) as a carrier to construct a novel nanomedicine for delivering paclitaxel (PTX).^[^ ^6d^ ^]^ This nanomedicine not only effectively delivered PTX to the lesion site, but also achieved significant combined therapeutic effects of immunomodulation and chemotherapy due to the unique immunomodulatory ability of IND.

Inspired by this, we herein chose IND to pair with FDA‐approved antitumor chemotherapeutic drugs (IND/antitumor drugs, the selection criteria for chemotherapeutic drugs are shown in Table S1, Supporting Information). Additionally, due to multiple therapeutic benefits provided by non‐steroidal anti‐inflammatory drugs (NSAIDs) in cancer treatment, including pain relief,^[^ ^9^ ^]^ reduction in cancer fever,^[^ ^10^ ^]^ promotion of tumor apoptosis,^[^ ^11^ ^]^ inhibition of tumor metastasis,^[^ ^12^ ^]^ suppression of drug resistance,^[^ ^13^ ^]^ sensitization of immunotherapy,^[^ ^14^ ^]^ and modulation of the tumor immune microenvironment,^[^ ^15^ ^]^ we selected doxorubicin (DOX) and paclitaxel (PTX), the most classic chemotherapeutic drugs, to be paired with FDA‐approved NSAIDs (NSAIDs/PTX and NSAIDs/DOX). These drugs were classified into groups A and B. All NSAIDs (Figure S1, Supporting Information) were assigned to group A (drug A), and antineoplastic drugs (Figure S2, Supporting Information) to group B (drug B). Meanwhile, through the literature review, we selected other drug combinations that have been confirmed to self‐assemble into nanomedicines to expand the dataset.^[^ ^3^ ^]^ These drug combinations were also randomly assigned into drug A and drug B (Figure S3, Supporting Information). We obtained a total of 87 drug combinations. The features of drugs A and B were derived from physicochemical parameters recorded in the DrugBank database and standardized by Z‐scores (Figure 1b,c; Figures S4 and S5 and Tables S2–S4, Supporting Information), and the labels were assigned to positive or negative to indicate whether drugs A and B could form a nanomedicine. Size, polydispersity index, and count rate are important properties of nanomedicine, and different application scenarios have different requirements for the above properties,^[^ ^16^ ^]^ there is no completely unified standard to evaluate whether a nanomedicine development research is successful or not, so we set a standard to judge whether the self‐assembled system we obtained is a nanomedicine or not, based on the previous research:^[^ ^16b^ ^]^ a particle size of less than 300 nm, a PDI value of less than 0.5, and a count rate of greater than 300. The dataset of 87 entries was divided into a training dataset and a test dataset at a ratio of 50:37. Subsequently, we chose three machine learning algorithms: Support Vector Machine (SVM), K‐Nearest Neighbors (KNN), and Logistic Regression (LR), to construct the most reliable prediction model as an AI screening platform for small molecule‐assembled nanomedicines (Figure 1a).

After predicting a nanomedicine formed from drug combinations in the test set, we will compare the predicted outcomes with the actual experimental results (Figure 1d; Figures S6 and S7, Supporting Information) to evaluate the predictive performance of the small molecule carrier‐nanomedicine screening platform, which is based on the three previously mentioned machine learning models.

The prediction performance was assessed from the confusion matrix generated from each machine learning model (Figure 1e). Since most drug combinations cannot form a nanomedicine, we considered those drug combinations to be a positive sample, which was labeled as “positive”. The accuracy, recall, and F1 score of three models were calculated from the confusion matrix (Equations S1–S3, Supporting Information), those specific results are listed in (Table S5, Supporting Information).

As these results are displayed in Table S5 (Supporting Information), the LR model had the highest accuracy, reaching 91.89% (34/37), while SVM had the lowest accuracy of 81.08% (30/37). In terms of precision, KNN had the highest precision of 100% (3/3), followed by 83.3% (5/6) from the LR model. It is noteworthy that KNN and SVM were biased to predict one category more accurately. In contrast, LR did not exhibit bias toward any of the four categories, resulting in a more balanced prediction probability. KNN had the highest recall rate, while SVM had the lowest recall rate. The LR model achieved the highest F1 score (Figure 1f). These results suggested the LR model was optimally suited for developing an AI platform to screen FDA‐endorsed candidate pharmaceuticals.

A comprehensive evaluation of the model performance was also performed by learning curves and receiver operating characteristic (ROC) curves. As the sample size increased, the scores for both training and test datasets in learning curves remained closely aligned across all three models, indicating an absence of overfitting (Figure 1g). However, due to a small dataset, the learning curve for SVM did not reach optimal training effectiveness, implying that expanding the dataset might improve its performance (Figure 1g). The area under the curve (AUC) of the ROC curves is commonly used to evaluate the effectiveness of prediction models. A higher AUC value indicates the model could be more effective in predicting the formation of nanomedicines. Figure 1h showed that LR had the highest AUC among the three models. It reached 0.9, indicating that the LR model had the highest prediction effectiveness. Meanwhile, a steeper ROC curve indicates the model is better at distinguishing between positive and negative samples at different thresholds. Among these three models, the ROC curve of the KNN model was relatively flat, suggesting that this model had a poor performance in predicting the formation of nanomedicines.

We acknowledge that a dataset of 87 entries is relatively small, reflecting the nascent stage of research in carrier‐free nanomedicines composed of FDA‐approved small molecules. However, the ML models, particularly LR, have demonstrated promising predictive capabilities despite the limited data. Moreover, ML models possess the inherent ability to self‐improve and become more accurate as additional data become available.^[^ ^17^ ^]^ Future work will involve expanding the dataset with newly discovered nanomedicine formulations, thereby enabling continuous retraining and refinement of the ML models to enhance their robustness and generalizability‐a key advantage of ML‐based frameworks.

Overall, the Logistic Regression model emerged as the most reliable predictor for identifying viable small molecule combinations capable of forming stable nanomedicines, with an accuracy rate of 91.89% and an AUC of ROC curve of 0.9. Therefore, the LR model is considered to be appropriate for constructing an AI screening platform for small molecule‐assembled nanomedicines, which underscores the potential of ML‐driven platforms in accelerating the discovery and optimization of carrier‐free nanomedicines.

### 2.2 The Mechanism for Driving the Formation of the Nanomedicines

Since DOX and PTX are widely used antineoplastic drugs in clinical treatment and their nanomedicines, such as LipoDOX and Abraxane, have been approved by the FDA,^[^ ^18^ ^]^ DOX and PTX were used in the present study as two representative antineoplastic drugs in order to shed light on the mechanism of the formation of their stable nanomedicines due to the interaction between non‐steroidal anti‐inflammatory drugs and antineoplastic drugs.

**Figure 2:**
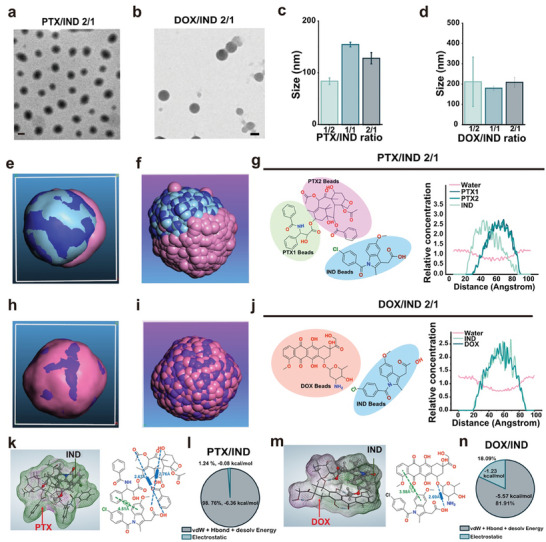
Preparation of nanomedicines containing representative antineoplastic drugs and self‐assembly mechanisms. a) Representative TEM image of the PTX/IND nanomedicine (iPTX) at a feeding ratio of 2:1. Scale bar = 100 nm; b) Representative TEM image of the DOX/IND nanomedicines (iDOX) at a feeding ratio of 2:1. Scale bar = 100 nm; c) Size distribution of iPTX at different feeding ratios; d) Size distribution of iDOX at different feeding ratios; e) 3D view of the PTX/IND mesostructure in a DPD force field at a PTX/IND feeding ratio of 2/1 after 100‐ns DPD simulation. The light pink, light blue, and blue colors represented the DPD force field of IND, PTX1, and PTX2 beads, respectively. PTX1 and PTX2 were two different parts of the PTX molecule; f) Molecular distribution of PTX and IND in iPTX at a PTX/IND feeding ratio of 2/1. IND molecules are shown as light pink sphere particles, and PTX molecules as light blue and blue sphere particles; g) The concentration profiles of various beads in iPTX at a PTX/IND feeding ratio of 2/1 after 100‐ns DPD simulation; h) 3D view of the DOX/IND mesostructure in a DPD force field at a DOX/IND feeding ratio of 2/1 after 100‐ns DPD simulation. The light pink and purple colors represented the DPD force field of IND and DOX beads, respectively; i) Molecular distribution of DOX and IND in iDOX at a DOX/IND feeding ratio of 2/1. IND molecules were shown as light pink sphere particles, and DOX molecules as purple sphere particles; j) The concentration profiles of DOX, IND, or water beads in iDOX at a DOX/IND mass ratio of 2/1 after 100‐ns DPD simulation; k) Rendering of hydrophilic/lipophilic regions and the chemical structure with intermolecular forces in iPTX after 100 ns MD simulation. The hydrophilic area was pink, the lipophilic area in green, and the neutral area in transparent white. The hydrogen bond was represented by a blue dotted line with a blue box in the middle, the π–π stacking by a green dotted line with double green hexagons in the middle, and the π‐hydrogen bond by a green dotted line with H and hexagon symbols in the middle; l) Ratios of different types of intermolecular forces in iPTX; m) Rendering of hydrophilic/lipophilic regions and the chemical structure with intermolecular forces in iDOX after 100 ns MD simulation. The hydrophilic area is presented in pink, the lipophilic area in green, and the neutral area in transparent white. The hydrogen bond was represented by a blue dotted line with a blue box in the middle, the π–π stacking by a green dotted line with double green hexagons in the middle, and the π‐hydrogen bond by a green dotted line with H and hexagon symbols in the middle; n) Ratios of different types of intermolecular forces in iDOX.

First, PTX and DOX nanomedicines were prepared at different feeding ratios using IND as a carrier. TEM results showed that both of them displayed a regular nanomorphology (**Figure** **2**a,b; Figures S8a and S9a, Supporting Information). The particle size of the PTX nanomedicine (iPTX) increased with an increase in the IND mass, while the zeta potential exhibited an opposite trend (Figure 2c; Figure S8b, Supporting Information). The size of the DOX nanomedicine (iDOX) was ≈200 nm at different DOX/IND feed ratios (Figure 2d). Interestingly, the zeta potential of iDOX increased with an increase in the DOX/IND feed ratio (Figure S9b, Supporting Information). The drug loading in both nanomedicines was positively correlated with their feeding amounts (Figures S10 and S11, Supporting Information). Herein, the drug loading of PTX in iPTX exceeded 77% when the PTX/IND feed ratio was 2/1, while the drug loading of DOX in iDOX reached 63% when the DOX/IND mass ratio was 2/1. Both drug loadings for PTX and DOX exceeded their reported levels in clinical nanomedicines.^[^ ^19^ ^]^

Dissipative particle dynamics (DPD) simulation of iPTX over a 100‐ns period (Figure 2e–g; Figures S12–S16 and Tables S6 and S7, Supporting Information) revealed that the IND beads preferred to acting as a shell to coat the PTX core (Figure 2f; Figures S14 and S15, Supporting Information). IND and PTX molecules were highly segregated in iPTX, rather than forming a homogeneous mixture (Figure 2g; Figure S16, Supporting Information). 100‐ns DPD simulation of iDOX (Figure 2h–j; Figures S12 and S17–S20, and Tables S8 and S9, Supporting Information) revealed that the IND beads were sporadically patched on the surface of the DOX/IND nanomedicine (Figure 2i; Figures S18 and S19, Supporting Information). The IND and DOX beads were homogeneously distributed within the nanomedicine (Figure 2j; Figure S20, Supporting Information).

To probe the mechanism for driving the formation of IND‐carried nanomedicines, the intermolecular interactions were unveiled from 100‐ns all‐atom molecular dynamics (MD) simulation via *Materials Studio 8.0* (Table S10, Supporting Information).^[^ ^6^, ^20^ ^]^ The predominant interactions include π–π stacking between aromatic rings of the antineoplastic drugs and IND, and hydrogen bonds between amino, ether, carbonyl, or hydroxyl groups of the antineoplastic drugs and carboxyl groups of IND (Figure 2k–n; Figure S21, Supporting Information). After 100‐ns MD simulation, the IND and these antineoplastic drugs could form stable amphiphilic complexes driven by these intermolecular interactions. Neutral drug molecules (such as PTX) can form molecular complexes with IND through van der Waals forces (such as hydrogen bonds and π–π stacking) (Figure 2k,l; Figure S22, Supporting Information). In contrast, weakly basic drugs with amino groups (such as DOX) can form amphiphilic complexes with IND through additional electrostatic interactions (>18%) on top of van der Waals forces (Figure 2m,n; Figure S23, Supporting Information).

Through the analysis of the intermolecular interactions between NSAIDs and both PTX and DOX in the formation of nanomedicines, we identified that π–π stacking and hydrogen bonding between NSAIDs and the benzene rings, hydroxyl, or carbonyl groups of PTX (Figure S22, Supporting Information) play a critical role in stabilizing PTX nanomedicines. Similarly, π–π stacking and hydrogen bonding between NSAIDs and the anthracycline, hydroxyl, and amino groups of DOX (Figure S23, Supporting Information), significantly contribute to the formation of DOX nanomedicines. These electrostatic interactions further enhance the driving force for the formation of DOX nanomedicines with NSAIDs. In addition, UV absorption spectra, Fourier transform infrared spectra (FTIR), and fluorescence spectra (Figures S24–S26, Supporting Information) confirmed the formation of nanomedicines from IND and PTX or DOX driven by these aforementioned intermolecular forces.

Notably, due to additional electrostatic interactions, iDOX displayed great stability in different dispersion media (Figures S27 and S28, Supporting Information). In particular, its microscopic shape and size in water could be maintained for up to 90 days (Figure S29, Supporting Information). In addition, the results shown in Table S11 (Supporting Information) suggested that rapid mixing of FDA‐approved drugs and NSAIDs in a microfluidic device may be an effective way to screen drug candidates for the construction of translatable nanomedicines.

### 2.3 The Functionalization of the Nanomedicines

The intricate mechanisms of diseases, particularly in oncology, necessitate nanomedicines with advanced functionalities to achieve optimal therapeutic outcomes. Simple drug combinations in small molecule carrier nanomedicines often fall short in addressing these complex requirements. Additionally, the challenges associated with industrial production and the stringent new drug approval processes mean that advanced functionalization techniques, such as incorporating complex synthetic polymers or chemically modifying drug structures, remain largely confined to experimental research stages. To overcome these limitations, we focused on rapidly functionalizing nanomedicines through purely physical modifications and by harnessing the inherent properties of the drug molecules themselves. This strategy enabled the development of nanomedicines with enhanced long‐circulation capabilities in vivo, environment‐responsive drug release, and active targeting of tumor sites, thereby meeting the increasing clinical demands for more sophisticated and effective cancer treatments.

#### 2.3.1 Prolonged Circulation Functionalization of IND‐Carried PTX Nanomedicines

**Figure 3:**
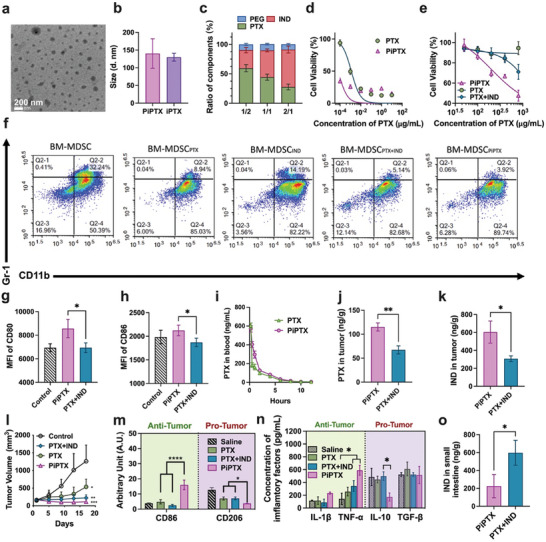
Preparation, characterizations, and anti‐tumor effects of the PEG‐modified PTX/IND nanomedicine (PiPTX). a) TEM image of PiPTX, scale bar = 200 nm; b) Particle sizes of iPTX and PiPTX. PiPTX at a PTX/IND feeding ratio of 2/1 had the morphology for anti‐tumor application (n = 3); c) The mass ratio of PTX, IND, and PEG in the nanomedicines at different PTX/IND feeding ratios. The contents of PTX and IND in the nanomedicine were positively correlated with their feeding weights. At a PTX/IND feeding ratio of 2/1, iPTX had the highest content of PTX, and ≈10% of PEG (n = 3); d) Cytotoxicity of PTX and PiPTX against the HepG‐2 cell line and e) cytotoxicity of different dosage forms of PTX (including PTX, PiPTX and a mixture of PTX and IND) against the A549‐MDR cell line; f) The populations of MDSCs after treatment with different dosage forms of PTX at an equivalent PTX concentration for 24 h; g) Mean fluorescence intensity of CD80 and h) CD86 expressed on the MDSCs after incubation with PiPTX or a mixture of PTX and IND; i) The concentration of PTX in the peripheral blood at different time points after treatment with PTX or PiPTX; j) The concentration of PTX and k) IND in the tumor tissue after treatment with PiPTX or a mixture of PTX and IND; l) Tumor growth curves in the HepG‐2 tumor‐bearing mice receiving different dosage forms of PTX (PTX, PiPTX, and PTX/IND) at an equivalent PTX concentration; m) Fluorescence intensity of CD86^+^ and CD206^+^ cells after immunostaining of tumor sections with APC‐CD68+FITC‐CD86 or APC‐CD68+FITC‐CD206, respectively; n) The concentration of anti‐tumoral cytokines IL‐1β and TNF‐α, and pro‐tumoral cytokines IL‐10 and TGF‐β in the tumor tissue upon completion of the treatment. PiPTX could convert a tumor immune‐suppressive microenvironment into an immune‐active one; o) The concentration of IND in the small intestine upon completion of the treatment. The data in the figures was shown as Mean ± SD. Significant differences were indicated as ^*^ for *p* < 0.05, ^**^ for *p* < 0.01,^***^ for *p* < 0.001, and ^****^ for *p* < 0.0001. Statistical significances were determined using the one‐way analysis of variance (ANOVA) test and Tukey's multiple comparison test.

One widely used strategy for prolonging blood circulation of a functionalized macromolecular‐derived nanomedicine is to incorporate a polyethylene glycol (PEG) layer on the nanomedicine.^[^ ^21^ ^]^ PEG‐coated iPTX (PiPTX) was prepared by adding a small amount of 1, 2‐distearoyl‐sn‐glycero‐3‐phosphoethanolamine‐polyethylene glycol (DSPE‐PEG), an FDA‐approved excipient, into an iPTX dispersion solution.^[^ ^22^ ^]^ PiPTX displayed a slightly larger size than iPTX, and a negatively charged surface (**Figure** **3**a,b; Figures S30 and S31, Supporting Information). The mass ratio of IND and PTX in PiPTX could be manipulated by tuning the feeding ratio of PTX/IND (Figure 3c). Notably, the content of DSPE‐PEG in PiPTX was found to be 9.0%–10.7%. PiPTX at a PTX/IND feeding ratio of 2/1 was selected after considering safe and effective therapeutic doses of IND and PTX.^[^ ^6^, ^23^ ^]^ PiPTX was found to have excellent stability in the cell culture medium, PBS, and deionized water (Figure S32, Supporting Information). Compared to PTX and a mixture of IND and PTX, PiPTX exhibited an enhanced in vitro anti‐tumor activity against HepG‐2 cells (a human hepatocellular carcinoma cell line) and A549‐MDR cells (a multi‐drug resistant tumor cell line), which may be attributed to physiochemical properties of the nanoformulation^[^ ^24^ ^]^ and MDR inhibition by IND (Figure 3d,e).^[^ ^25^ ^]^ Both IND and PTX have been shown to effectively reduce the population of myeloid‐derived suppressor cells (MDSCs) in the tumor environment for activating tumor immunity.^[^ ^26^ ^]^ A stronger effect on inhibiting the differentiation of bone marrow‐derived stem cells (B‐MDSCs) to MDSCs was found by the combination of IND and PTX in the PiPTX nanomedicine in comparison with that by IND, PTX, and their physical mixture (Figure 3f; Figure S33, Supporting Information). The treatment with PiPTX boosted the expression of CD80 and CD86 on B‐MDSCs in the tumor‐conditioned medium (Figure 3g,h). PiPTX with a coating layer of PEG achieved prolonged circulation in vivo and the plasma concentration of PTX was significantly increased. A greater accumulation level of PTX and IND within tumors was realized through PiPTX compared to IND, PTX, and their physical mixture (Figure 3i–k).

Hepatocellular carcinoma is insensitive to chemotherapeutic drugs,^[^ ^27^ ^]^ and its treatment with systemic chemotherapeutic agents has been demonstrated to be ineffective.^[^ ^28^ ^]^ PiPTX was applied to the mice bearing hepatocellular carcinoma. Impressively, PiPTX displayed a significant inhibitory effect on tumor growth in the HepG‐2‐bearing nude mice by inhibiting cell proliferation and promoting tumor apoptosis (Figure 3l; Figures S34 and S35, Supporting Information). Because IND could prevent tumor‐induced MDSCs accumulation and monocytes differentiation toward tumor‐supportive M2‐like macrophages,^[^ ^14^, ^29^ ^]^ treatment with the IND‐derived PiPTX nanomedicine led to a significant increase in the percentage of CD86 positive cells and a decrease in the percentage of CD206 positive cells in the tumor tissue (Figure 3m; Figure S36, Supporting Information). An increase in the concentration of anti‐tumoral cytokines (IL1‐β and TNF‐α) and a decrease in the concentration of pro‐tumoral cytokines (IL‐10 and TGF‐β) were also found in the tumor microenvironment after treatment with PiPTX (Figure 3n). This promising therapeutic effect could be attributed to a high drug concentration in the tumor tissue achieved through prolonged circulation of PiPTX, simultaneous delivery of PTX and IND into tumor cells, and synergistic immunomodulatory and chemotherapeutic effects by PTX and IND.

Although the mixture of IND and PTX (PTX+IND) exhibited distinct in vivo anti‐tumor effects, a death event occurred during the treatment due to severe intestinal injury induced by free drugs (Figure S37, Supporting Information). In contrast, there was no death event in the PiPTX‐treated group. No significant damage to main organs and the intestine was seen throughout the course of the treatment (Figures S37 and S38, Supporting Information), which may be ascribed to a decrease in the distribution of IND in the small intestine which could result from a pronounced reduction in the hepato‐intestinal accumulation of PiPTX (Figure 3o).

These findings suggested that PiPTX could attain longer circulation of IND and PTX compared to their free counterparts. A high drug loading in PiPTX and its nanostructure for passive accumulation resulted in a high retention concentration of PTX and IND in tumors. Furthermore, PiPTX enhanced the immunomodulatory effect of IND on MDSCs and improved the anti‐tumor efficacy of PTX, thus it could effectively treat tumors.

#### 2.3.2 Tumor‐Environment Responsive Release from IND‐Carried DOX Nanomedicines

**Figure 4:**
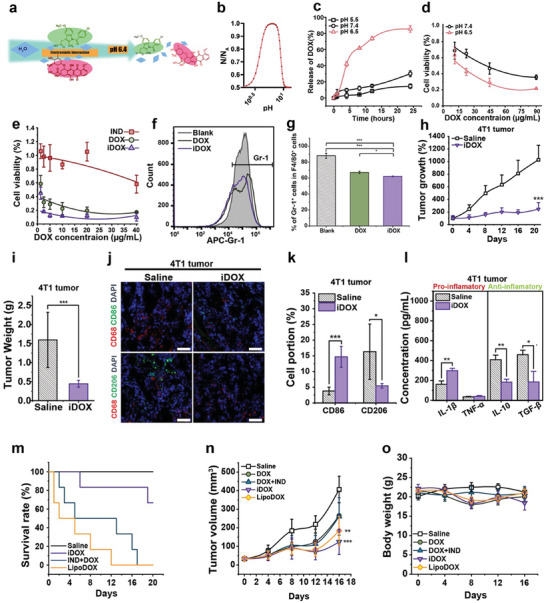
Preparation, characterizations, and anti‐tumor effects of iDOX. a) Schematic diagram of the dissociation of iDOX by water molecules. b) The ionization level of iDOX at feeding ratios of 2/1 (m_DOX_:m_IND_) under pH 6.5. N = number of particles and N_A_ = the Avogadro constant. c) Release of DOX from iDOX at different pH values; d) Cytotoxicity of iDOX against the HepG‐2 cell line at different pH values. iDOX at pH 6.5 had a higher release efficiency than that at pH 7.4, leading to better cytotoxicity against the tumor cell line; e) Cytotoxicity against the A549‐MDR cell line after incubation with IND, DOX, or iDOX for 48 h; f) The level of Gr‐1 in MDSCs after treatment with DOX or iDOX. iDOX could reverse the immunosuppressive phenotype of MDSCs by reducing the expression of Gr‐1; g) The percentage of Gr‐1‐positive cells in MDSCs (F4/80 positive) after treatment with DOX or iDOX; h) The average tumor growth rate and i) the average weight of tumors in a 4T1 xenograft tumor model after treatment with saline or iDOX; j) Immunofluorescent staining images of the tumor tissues after treatment with saline or iDOX (scale bar = 50 µm) and k) Ratios of CD86^+^ and CD206^+^ cells in these tumor tissues labeled with APC‐CD68/FITC‐CD86 or APC‐CD68/FITC‐CD206 in a 4T1 xenograft tumor model. CD68 was labeled with the APC‐CD68 antibody (red), CD206 or CD86 with the FITC‐CD206 antibody (green) or the FITC‐CD86 antibody (green), and the nuclei with DAPI (blue); l) The concentration of anti‐tumoral cytokines (IL‐1β and TNF‐α) and pro‐tumoral cytokines (IL‐10 and TGF‐β) in the tumor tissue after treatment with saline and iDOX in a 4T1 xenograft tumor model. iDOX could convert a tumor immune‐suppressed microenvironment into an immune‐activated one; m) Survival rates, n) tumor volumes, and o) body weights of the Balb/c mice bearing 4T1 xenograft tumor after treatment with different dosage forms of DOX. Representative images were used from three independent parallel experiments. The data in the figures was shown as the Mean ± SD. Significant differences were distinguished by ^*^ for *p* < 0.05, ^**^ for *p* < 0.01, ^***^ for *p* < 0.001, and n.s. for *p* > 0.05.

Novel pH‐responsive nanomedicines have emerged by preparing through electrostatic interactions between inert polymer‐carriers and specific drug molecules.^[^ ^30^ ^]^ Inspired by this discovery, we hypothesize that NSAIDs‐carried nanomedicines, such as the IND/DOX nanomedicine (iDOX), could be endowed with similar pH‐responsive characteristics through intermolecular electrostatic interaction by tuning the ratio of IND/DOX. We calculated the ionization degree of IND and DOX in iDOX at different pH values using the Henderson–Hasselbalch equation.^[^ ^30^, ^31^ ^]^ The ionization level of iDOX at a DOX/IND feeding ratio of 2/1 displayed an unimodal distribution curve with a peak at pH 6.5 (**Figure** **4**a,b; Figure S39, Supporting Information).^[^ ^32^ ^]^ Thus, the iDOX nanomedicine at a DOX/IND feeding ratio of 2/1 was prepared as mentioned before (Figure 2b). The DOX nanomedicine showed a more rapid DOX release pattern at pH 6.5 than that at pH 5.5 and 7.4 (Figure 4c). Since the pH of the tumor microenvironment is ≈6.5, iDOX exhibited superior cytotoxicity against HepG‐2 cells in the cell culture medium at pH 6.5 compared to pH 7.4 (Figure 4d). Meanwhile, compared to other DOX dosage forms, the highest level of cytotoxicity toward three tumor cell lines, particularly A549‐MDR, a multidrug‐resistant tumor cell line, was achieved by iDOX (Figure 4e; Figures S39–S42, Supporting Information). Due to the pH of 7.4 within tumor cells and 5.5 within lysosomes, the release of iDOX is significantly slower at these pH levels compared to its release behavior at pH 6.5. This slower release extends the action time of DOX once it enters the tumor cells. This characteristic is confirmed by fluorescence microscopy images (Figure S43, Supporting Information). In tumor‐conditioned media, where the pH is ≈6.5, iDOX rapidly releases IND, effectively modulating myeloid‐derived suppressor cells (MDSCs) and converting them into immune‐supportive cells (Figure 4f,g).

In both HepG‐2 and 4T1 tumor‐bearing mice models, iDOX displayed the most potent in vivo anti‐tumor effects (Figure 4h,i; Figures S44 and S45, Supporting Information). Immunofluorescence staining of tumor tissues and ELISA results confirmed that treatment with iDOX helped reduce immunosuppressive macrophages in the tumor region and effectively reversed the immunosuppressive environment (Figure 4j–l; Figures S44e–g and S45e–h, Supporting Information). Notably, iDOX exhibited a superior biosafety profile at a lower financial cost compared to a commercially available nano‐formulation of doxorubicin (LipoDOX) (Table S11, Supporting Information). The antitumor effects of iDOX and LipoDOX (DOX dose of 3 mg kg^−1^) were similar. On the other hand, when the DOX‐equivalent dose was 5 mg kg^−1^, the 20‐day mortality rate with iDOX was 40%, whereas LipoDOX resulted in a 100% mortality rate within 12 days (Figure 4m–o).^[^ ^33^ ^]^

These above results supported that iDOX exhibited a highly specific response to a low pH in the tumor microenvironment, as well as a sustained drug release pattern in tumor cells, resulting in remarkable anti‐tumor effects in both nude mice models of human liver cancer and breast cancer. Furthermore, our results suggested that iDOX outperformed LipoDOX in terms of cost‐effectiveness (Table S12, Supporting Information), safety, and efficacy.

#### 2.3.3 Tumor Targeting Ability of Laminarin‐Modified iDOX

**Figure 5:**
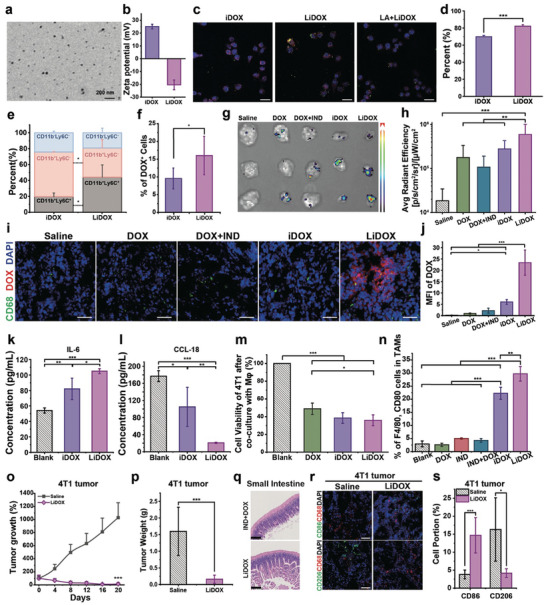
Preparation, characterizations, and anti‐tumor activities effects of LA‐modified DOX/IND nanomedicines (LiDOX). a) TEM image (Scale bar = 200 nm), and b) zeta potentials of iDOX and LiDOX. The morphology of the nanomedicine modified with laminarin appeared similar to that without modification; c) Fluorescent images of RAW 264.7 cells cultured with iDOX, LiDOX or LA+LiDOX (sequentially treated with LA and LiDOX) under confocal microscopy (Scale bar = 20 µm); d) The efficiency for phagocytosis of iDOX or LiDOX by RAW 264.7 cells; e) The phagocytosis level of iDOX and LiDOX by different monocytes subsets; f) The percentage of DOX‐containing cells in peripheral blood. LiDOX had a stronger ability to enter peripheral leukocytes than iDOX; g) Ex vivo fluorescent images, and h) the fluorescence intensity of excised tumors at the end of different dosage forms of DOX treatment; i) Representative immunofluorescence images (scale bar = 50 µm) for the distribution of DOX and CD68^+^ cells in the tumor tissue. CD68 was labeled with the FITC‐CD68 antibody (green). DOX was in red, and the nucleus was in blue; j) Mean fluorescent intensity of DOX in the tumor tissue after the intravenous intervention of different dosage forms of DOX; k) The concentration of IL‐6 (a pro‐inflammatory cytokine) and l) CCL‐18 (an anti‐inflammatory cytokine) in the RAW 264.7 cell line after treatment with saline, iDOX or LiDOX. LiDOX could more efficiently stimulate macrophages to pro‐inflammatory due to its macrophage‐targeting ability; m) Cell viability of the 4T1 cell line after co‐incubation with macrophages that were pre‐treated with different dosage forms of DOX; n) The percentages of CD80^+^ and F4/80^+^ cells in TAMs after treatment with different dosage forms of DOX; o) Tumor growth rates and p) tumor weights in a 4T1 tumor model after treatment with saline or LiDOX; q) H&E staining images of the small intestine after treatment with LiDOX and a mixture of IND and DOX (scale bar = 100 µm); r) Immunostaining images (scale bar = 50 µm) of the tumor tissue and s) the percentage of CD86^+^ and CD206^+^ cells in these 4T1 tumor tissues labeled with APC‐CD68 (red)/FITC‐CD86 (green) or APC‐CD68 (red)/FITC‐CD206 (green), and the nuclei were labeled with DAPI (blue). Representative images were from independent experiments (n = 3). The data in the figures was presented as the average ± SD. Significant differences were distinguished by ^*^ for (*p* < 0.05), ^**^ for (*p* < 0.01), and ^***^ for (*p* < 0.001).

Laminarin (LA), a primary component of *kelp (Laminaria)*, is a typical ligand for the Dectin‐1 receptor and it is found in traditional Chinese medicines listed in the Chinese Pharmacopoeia.^[^ ^34^ ^]^ Given a high expression level of Dectin‐1 on the surfaces of both M‐MDSCs and monocytes,^[^ ^35^ ^]^ we hypothesize that coating iDOX with laminarin could empower iDOX with the ability to target M‐MDSCs and monocytes. After migration of these immune cells loaded with laminarin‐modified iDOX to the tumor site, DOX and IND released from LiDOX could exert their therapeutic effects on tumor cells.^[^ ^36^ ^]^ Through electrostatic interaction, we successfully obtained laminarin‐modified iDOX (LiDOX) (**Figure** **5**a,b) and confirmed the targeting ability of LiDOX for M‐MDSCs and monocytes (Figure 5c–f; Figures S46 and S47, Supporting Information). As a result, LiDOX was seen to be significantly enriched in the tumor tissue compared with other DOX dosage forms (Figure 5g–j; Figures S48 and S49, Supporting Information).

We thoroughly evaluated the impact of LiDOX on the phenotypes and immune functions of RAW264.7 cells and assessed its enhanced anti‐tumor activity in 4T1 (Figure 5k–n; Figure S50, Supporting Information). It was found that LiDOX had no significant impact on the cell viability of RAW264.7 cells (Figure S51, Supporting Information). LiDOX displayed a more potent therapeutic effect than iDOX on both 4T1 and HepG‐2 animal models (Figure 5o,p; Figures S44 and S45, Supporting Information). This enhanced therapeutic effect may be attributed to the unique targeting ability of LiDOX toward M‐MDSCs and monocytes. Notably, the LiDOX‐treated mice group exhibited a significantly prolonged survival rate than the free drug‐treated group in a 4T1 tumor animal model (Figure S52, Supporting Information), which may be due to minimal damage to the intestine and heart by LiDOX compared to free DOX during transport of LiDOX by monocytes to the tumor site (Figure 5q; Figure S53, Supporting Information). Additionally, LiDOX treatment could significantly increase the number of immune‐active macrophages and decrease the number of immunosuppressive tumor‐associated macrophages (TAMs) in the tumor tissue (Figure 5r,s; Figures S44e–g and S45e–h, Supporting Information). These findings indicated that LiDOX not only enhanced the chemotherapeutic effect of DOX through precise targeting of immune cells with migratory behavior toward tumors via the laminarin moiety but also more effectively exerting the immunomodulatory effects of IND rely on its ability to actively target monocytes, thereby synergistically improving the efficacy of anti‐tumor therapy.

## 3 Conclusion

Leveraging machine learning algorithms, physicochemical parameters of small molecular drugs were used as features to assess their assembly into nanomedicines. An artificial intelligence screening platform was successfully established to identify small molecular drugs as a carrier for constructing nanomedicines with a predictive accuracy of above 91%. Among these small molecule carriers for nanomedicines predicted from the model, we selected two widely used antineoplastic drugs to assemble with INDs to elucidate their binding mechanisms, which could lay a crucial theoretical foundation for refining the screening platform. To meet clinical demands, we have functionalized the aforementioned nanomedicines through supramolecular interactions, facilitated by a simple ultrasonic mixing process, achieving clinically urgent functionalities such as in vivo prolonged circulation, tumor‐responsive drug release, and active tumor targeting. The successful establishment of the aforementioned screening platform and the proposal of functionalization strategies for nanomedicines constructed by small molecule drug carriers could open a new avenue for potent and safe anti‐tumor drug nanoformulations with significant translational potential. Furthermore, we are currently developing an online platform for drug assembly, which leverages community‐driven practice and feedback to continuously optimize the predictive model, thereby accelerating the advancement of drug‐drug‐assembled nanomedicines.

## 4 Experimental Section

Detailed experiments and methods, and other data are available in the Supporting Information. Implementation of screening, evaluation, and establishment of a machine learning screening platform using Python Software. All mouse experiments were performed in accordance with protocols approved by the Chongqing University of Technology Ethics Committee. Statistical analysis was performed using OriginPro Trail (OriginLab Corporation, Northampton, Massachusetts, USA) to calculate the mean and SD for each experimental group as well as the control group. Statistical significances were determined using the one‐way analysis of variance (ANOVA) test and Tukey's multiple comparison test. A *p* value <0.05 was considered to be significant.

## Conflict of Interest

The authors declare no conflict of interest.

## Supporting information



Supporting Information

## Data Availability

The data that support the findings of this study are available from the corresponding author upon reasonable request.
