# Supplementary material for: Machine Learning‐Driven Prediction, Preparation, and Evaluation of Functional Nanomedicines Via Drug–Drug Self‐Assembly
Source: Adv Sci (Weinh). 2025 Jan 10;12(9):2415902. doi: 10.1002/advs.202415902 (PMC11884566; doi:10.1002/advs.202415902)
Supplement: Supplementary file 1 — Supporting Information [file ADVS-12-2415902-s001.docx]

Supporting Information

**Machine Learning-Driven Prediction, Preparation, and Evalution of Functional Nanomedicines via Drug-Drug Self-Assembly**

*Chengyuan Zhang, Yuchuan Yuan, Qiong Xia, Junjie Wang, Kangkang Xu, Zhiwei Gong, Jie Lou, Gen Li, Lu Wang, Li Zhou, Zhirui Liu, Kui Luo*, Xing Zhou**

Prof. C. Zhang, Prof. X. Zhou

Yunnan Key Laboratory of Stem Cell and Regenerative Medicine, Kunming Medical University, Kunming 650500, China

*E-mail: [diszhou@126.com](mailto:diszhou@126.com) & [zhouxing@kmmu.edu.cn](mailto:zhouxing@kmmu.edu.cn)

Q. Xia, J. Wang, K. Xu, Z. Gong, Prof. J. Lou, Prof. G. Li, Dr. L. Wang

Department of pharmacy, School of Pharmacy and Bioengineering, Chongqing University of Technology, Chongqing 400054, China.

Dr. Y. Yuan

School of Medicine, Northwest University, Xi’an 710068, China.

Dr. Z. R. Liu

Department of pharmacy, Xinan hospital, Army Medical University, Chongqing 400038, China.

L. Zhou

Department of Biomedical Engineering, School of Engineering, China Pharmaceutical University, Nanjing 210009, China.

Prof. K. Luo

Department of Radiology, Huaxi MR Research Center (HMRRC), Institution of Radiology and Medical Imaging, Breast Center, Institute of Breast Health Medicine, State Key Laboratory of Biotherapy, West China Hospital, Sichuan University, Chengdu 610041, China.

*E-mail: [luokui@scu.edu.cn](mailto:luokui@scu.edu.cn)

Content

[1. Experimental Section 1](#_Toc176275523)

[1.1 Materials 1](#_Toc176275524)

[1.2 Animals 1](#_Toc176275525)

[1.3 Preparation of self-assemblies 2](#_Toc176275526)

[1.3.1 Self-assemblies of antineoplastic drugs with IND 2](#_Toc176275527)

[1.3.2 Self-assemblies of PTX with NSAIDs 2](#_Toc176275528)

[1.3.3 Self-assemblies of DOX with NSAIDs 2](#_Toc176275529)

[1.4 Building a machine learning prediction platform for nanomedicines using FDA approved small molecular drugs as carriers 2](#_Toc176275530)

[1.5 Calculation of drug interactions and intermolecular forces via Autodock. 3](#_Toc176275531)

[1.6 Intermolecular dynamics calculated via DPD simulation. 3](#_Toc176275532)

[1.7 Preparation of LiDOX and PiPTX 4](#_Toc176275533)

[1.8 Physicochemical properties of nanomedicines. 4](#_Toc176275534)

[1.9 Stability of iDOX and PiPTX. 5](#_Toc176275535)

[1.10 Drug loading efficiency of iDOX or PiPTX. 5](#_Toc176275536)

[1.11 Release behavior of iDOX. 5](#_Toc176275537)

[1.12 In vitro anti-tumor effects 5](#_Toc176275538)

[1.13 Cytotoxicity of iDOX under different pH 6](#_Toc176275539)

[1.14 The effect of LiDOX on the viability of macrophages. 6](#_Toc176275540)

[1.15 The effect of LiDOX on the phagocytic activity of macrophages. 6](#_Toc176275541)

[1.16 In vitro uptake of LiDOX by RAW 264.7 cells 7](#_Toc176275542)

[1.17 Tumor-bearing mice models 7](#_Toc176275543)

[1.18 In vivo distribution of nanomedicines 7](#_Toc176275544)

[1.18.1 Concentration of PTX and IND in peripheral blood 7](#_Toc176275545)

[1.18.2 Concentration of PTX and IND in the tumor and small intestine 7](#_Toc176275546)

[1.18.3 In vivo distribution of iDOX and LiDOX 8](#_Toc176275547)

[1.19 In vivo anti-tumor effects 8](#_Toc176275548)

[1.19.1 Treatment regimen 8](#_Toc176275549)

[1.19.2 Assessment of biosafety 9](#_Toc176275550)

[1.19.3 Levels of proliferation and apoptosis within tumor tissues. 9](#_Toc176275551)

[1.20 Immune activation by nanomedicines. 9](#_Toc176275552)

[1.20.1 Modulation towards macrophage cells. 9](#_Toc176275553)

[1.20.2 Modulation towards TAMs. 10](#_Toc176275554)

[1.20.3 Modulation towards bone marrow-derived macrophages. 10](#_Toc176275555)

[1.20.4 Analysis of the tumor site immune environment 10](#_Toc176275556)

[2. Supplementary Figures 12](#_Toc176275557)

[3. Supplementary Tables 38](#_Toc176275558)

# Experimental Section

## Materials

Antineoplastic drugs (amsacrine, azathioprine, belinostat, bendamustine, bortezomib, cabazitaxel, carfilzomib, carmustine, cyclophosphamide, daunorubicin, diethylstilestrol, doxorubicin, epirubicin, etoposide, idarubicin, ifosfamide, irinotecan, melphalan, methylprednisolone, mitoxantrone, paclitaxel, pixantrone, pralatrexate, raltitexed, tacrolimus, temsirolimus, teniposide, valproic acid, verteprofin, vinblastine, vincristine, vinflunine, and vinorelbine) and NSAIDs (indomethacin, sulindac, ketoprofen, aceclofenac, aspirin, celecoxib, diclofenac, etodolac, fenoprofen, flufenamic acid, ketorolac, lornoxicam, mefenamic, meloxicam, naproxen, nimesulide, piroxicam, salicylic acid, tenoxicam, tolfenamic acid, and ursodeoxycholic acid) were purchased from MedChemExpress (USA); HCl, NaOH, laminarin, DSPE-PEG, M-CSF, a TUNEL/Caspase-3 detection kit, and a TUNEL/Ki67 detection kit were purchased from Sigma-Aldrich (USA); dimethyl sulfoxide (DMSO), methanol (CH_3_OH), and 4% paraformaldehyde were supplied by Aladdin Reagent Co., Ltd. (Shanghai, China); a H&E staining kit and DAPI were purchased from Servicebio (Wuhan, China); O.C.T compounds, a CCK-8 assay kit, and ELISA kits (IL-1β, IL-10, TGF-β, TNF-α, IL-6 and CCL-18) were purchased from Beyotime (Shanghai, China); antibodies (FITC-CD68, FITC-CD11b, APC-CD80, APC-CD86, FITC-Ly6C, APC-Gr-1, APC-CD206 and PE-Ly6C) were purchased from Thermo Fisher Scientific (MA, USA). 4T1, A549, A549-MDR, Raw 264.7, THP-1 and HepG-2 cell lines were supplied by ATCC (USA); FBS, DMEM and RPMI-1640 culture media were purchased from Gibco (USA); LPS, IL-4, IL-13, and IFN-γ were purchased from R&D Systems (USA).

## Animals

All animal care and experimental protocols were performed in compliance with the Animal Management Rules of the Ministry of Health of the People's Republic of China and the guidelines for Care and Use of Chongqing University of Technology (Approval No. 2022178 ).

## Preparation of self-assemblies

### Self-assemblies of antineoplastic drugs with IND

An antineoplastic drug and IND were dissolved in DMSO at a weight ratio of 1:1 (2 mg/2 mg). The DMSO solution was slowly dropped into ddH_2_O on a vortex mixer. After the precipitate was centrifugated at 14000 rpm at 4 ˚C for 15 min under protection from light, the precipitation products were collected and re-dispersed in ddH_2_O for subsequent studies.

### Self-assemblies of PTX with NSAIDs

PTX and NSAIDs were dissolved in DMSO at a weight ratio of 1:1 (2 mg/2 mg). The DMSO solution was slowly dropped into ddH_2_O on a vortex mixer at 4 ˚C. The water suspension was centrifugated at 14000 rpm at 4 ˚C for 15 min, and the precipitation products were collected and re-dispersed in ddH_2_O for subsequent studies.

### Self-assemblies of DOX with NSAIDs

DOX and NSAIDs were dissolved in DMSO at a weight ratio of 1:1 (2 mg/2 mg). The DMSO solution was slowly dropped into ddH_2_O on a vortex mixer at 4 ˚C. The water suspension was centrifugated at 14000 rpm at 4 ˚C for 15 min, and the precipitation products were collected and re-dispersed in ddH_2_O for subsequent studies.

## Building a machine learning prediction platform for nanomedicines using FDA approved small molecular drugs as carriers

FDA-approved non-steroidal anti-inflammatory drugs (NSAIDs) were paired with small-molecule anticancer chemotherapeutics, integrating existing research on small-molecule-based nanomedicines, resulting in 87 drug combinations. Drug parameters were sourced from the publicly available DrugBank dataset. The dataset was partitioned into training and test sets in a 50:37 ratio. Drug parameter features underwent Z-score normalization, followed by training and prediction using three machine learning models: Support Vector Machine (SVM), K-Nearest Neighbors (KNN), and logistic regression (LR). Model performance was assessed by calculating accuracy, recall, and F1 score, and by plotting confusion matrices. Based on the confusion matrix results, learning curves and ROC curves were generated using Python software. ROC curves were generated by calculating true positive rates and false positive rates at different thresholds. The area under the curve (AUC) was used to quantify overall model classification performance. The methods for calculating accuracy, recall, and F1 score are detailed in Equations (1)-(3).

$\text{A=}\frac{\text{TP+TN}}{\text{N}}$ Equation (1)

$\text{R=}\frac{\text{TP}}{\text{TP+FN}}$ Equation (2)

$\text{F=}\frac{\text{2×TP}}{\text{2}\text{×T}\text{P+FN+FP}}$ Equation (3)

Where A is for accuracy, R for recall and F fort the F1 score. TP, TN, FP, and FN are true positive, true negative, false positive, and false negative values shown in the confusion matrix. N is the number of samples.

## Calculation of drug interactions and intermolecular forces *via* Autodock.

Molecular structures of antineoplastic drugs (amsacrine, azathioprine, belinostat, bendamustine, bortezomib, cabazitaxel, carfilzomib, carmustine, cyclophosphamide, daunorubicin, diethylstilestrol, doxorubicin, epirubicin, etoposide, idarubicin, ifosfamide, irinotecan, melphalan, methylprednisolone, mitoxantrone, paclitaxel, pixantrone, pralatrexate, raltitexed, tacrolimus, temsirolimus, teniposide, valproic acid, verteprofin, vinblastine, vincristine, vinflunine, and vinorelbine) and NSAIDs (indomethacin, sulindac, ketoprofen, aceclofenac, aspirin, celecoxib, diclofenac, etodolac, fenoprofen, flufenamic acid, ketorolac, lornoxicam, mefenamic, meloxicam, naproxen, nimesulide, piroxicam, salicylic acid, tenoxicam, tolfenamic acid, and ursodeoxycholic acid) were downloaded from DrugBank (https://www.drugbank.ca/). They were optimized through geometry optimization via Materials Studio 2017 software (Accelrys Inc.). The optimized structures of two drugs were imported into the Autodock 4.2 software. One drug was set as a receptor and the other as a ligand. The grid box was confined to a dimension of 126 Å× 126 Å× 126 Å, and the distance between two discrete grid points was 0.0375 nm. The Lamarckian GA, a genetic algorithm, a simulated annealing algorithm, and a local search algorithm were used for calculations. Other parameters were set by default.

## Intermolecular dynamics calculated *via* DPD simulation.

First, PTX, DOX, IND, and water molecules were coarse-grained to their corresponding beads. Flory-Huggins interaction parameters (χ_ij_) between two beads were calculated by the Blends Module in MS (Table S4). DPD simulation was conducted at an ultra-fine grid level to investigate the self-assembly process of PTX1/PTX2/IND or DOX/IND at a PTX1/PTX2/IND/water ratio of 1/1/1/18 or a DOX/IND/water ratio of 1/1/18, respectively. The maximum repulsion between the coarse grain i and the grain j, α_ij,_ was obtained from the equation: α_ij_ (ρ = 3) = 78 + 3.25χ_ij_ (Table S3, 5). Consequently, the size and number of the simulated aggregates were used to determine the simulation box size and the integration time step. DPD simulations were performed in a box of 100 × 100 × 100 r_c_^3^ for 100 ns, where r_c_, a DPD length unit or the cut-off radius, was 6.46 Å in our simulations. Thus, the box size in our simulations was defined with an effective dimension of 1938Å × 1938Å × 1938Å. The simulations were performed via the Mesocite program incorporated in *Materials Studio 2017* at 298 K, and the total simulation time was 100 ns with an integration time step of 150 fs.

## Preparation of LiDOX and PiPTX

DOX/IND or PTX/IND was self-assembled into iDOX or iPTX, respectively. Laminarin at a weight of ten times of DOX was added to the iDOX aqueous solution. After laminarin was completely dissolved, the mixture was centrifuged at 14,000 rpm for 10 min under the protection from light at 4 ˚C, and the supernatant was discarded to remove unbounded laminarin. The solid pellet was re-dispersed in ddH_2_O to obtain LiDOX. DSPE-PEG at a weight of ten times of PTX was dissolved in DMSO to reach a concentration of more than 0.5 mg/mL, and the DSPE-PEG solution was added to the iPTX aqueous solution dropwise in an ultrasound field. The mixture was centrifuged at 14,000 rpm for 10 min at 4 ˚C and the supernatant was discarded to remove the unbounded DSPE-PEG. The pellet was re-dispersed in ddH_2_O to obtain PiPTX.

## Physicochemical properties of nanomedicines.

Lyophilized nano-assemblies were sampled for FTIR spectra and UV-Vis spectra. The FTIR spectra of nano-assemblies was acquired via an FTIR spectrophotometer (PerkinElmer, Germany) in a reflectance mode within the wavenumber region of 500–4000 cm^−1^, while UV-Vis spectra via a UV spectroscopy (UV-1700 Hitachi High Technologies, Tokyo). The size distribution, PDI, and zeta potential of nano-assemblies were measured via laser granulometry (Nano Zetasizer, Malvern Instruments) at room temperature. A drop of the lyophilized nano-assemblies solution was dripped onto the formvar coated copper mesh, dried at room temperature without staining and observed under a transmission electron microscope (TEM). MALDI-TOF MS analysis of PiPTX was performed on MALDI-7090 (Shimadzu, Japan).

## Stability of iDOX and PiPTX.

The prepared PiPTX was lyophilized and dispersed in ddH_2_O, PBS or the DMEM medium. Digital photos of samples were taken at 0 h, 12 h and 24 h. iDOX at a feeding weight ratio (m_DOX_/m_IND_) of 4/1, 2/1, 1/1, 1/2 or 1/4 was prepared and stored in a sterile EP tube. Digital photos of samples were taken after 24 h. iDOX at a feeding ratio of 2/1 was dispersed in ddH_2_O or PBS, and a small amount of samples were withdrawn at 0 h, 12 h and 24 h for TEM images. To investigate long-term stability of iDOX, iDOX at a DOX/IND feeding ratio of 2/1 was stored in a sample vial protected from light at room temperature. Digital photos were taken on day 0, 7, 30 and 90. After 90-day storage, a small amount of iDOX was sampled and lyophilized for TEM images.

## Drug loading efficiency of iDOX or PiPTX.

iDOX and PiPTX at different feeding ratios were prepared. They were lyophilized, weighed and re-dissolved in methanol (CH_3_OH). The contents of PTX in iPTX and PiPTX were determined by high-performance liquid chromatography (HPLC; LC-20A, Shimadzu, C8 column 4.6 × 150 mm. Mobile phase A: water with 0.02% acetic acid, 45%. Mobile phase B: acetonitrile, 55%. Flow rate was 1 mL/min, and analysis time was 15 min). The contents of DOX in iDOX and LiDOX were determined via a fluorescence spectrophotometer (E_x_: 478 nm, E_m_: 596 nm). The drug loading (%) = (The loaded drug mass)/(The total drug mass) × 100%. The content of PEG (%) = 100% - PTX loading (%) - IND loading (%).

## Release behavior of iDOX.

An iDOX aqueous solution was transferred into a dialysis bag (1.5 kDa) and incubated in PBS at different pH values in a water bath at 37 ˚C for 24 h. 1 mL of the buffer solution was collected at different time points and analyzed for the DOX content using a spectrofluorometer. 1 mL of fresh PBS was replenished after each sample collection to maintain the same volume of PBS outside of the dialysis bag.

## *In vitro* anti-tumor effects

The HepG-2 cell line, the 4T1 cell line, the paclitaxel-resistant A549 (A549/MDR) cell line and the RAW 264.7 cell line was cultured in the DMEM media or RPMI-1640 media containing 10% fetal bovine serum (FBS), and antibiotics including penicillin (100 IU/mL) and streptomycin (100 mg/L). The cells were generously donated by the School of Pharmacy at the Army Medical University. CCK-8 assays were employed to evaluate the cytotoxicity of nanomedicines. Cells were seeded at a density of 6×10^3^ cells per well in 96-well plates (Corning, NY, USA). PTX or PiPTX was dispersed in the cell culture medium of the HepG-2 cell line at various concentrations and the cells in the medium were incubated for 48 h; PTX, a PTX/IND mixture or PiPTX was dispersed in the cell culture medium of the A549/MDR cell line at various concentrations and these cells were incubated for 48 h; DOX, a DOX/IND mixture, iDOX, or IND was dispersed in the cell culture medium of the HepG-2 cell line or the 4T1 cell line at various concentrations and these cells were incubated for 48 h; DOX, iDOX, or IND was dispersed in the cell culture medium of the A549/MDR cell line at various concentrations and these cells were incubated for 12 or 48 h; DOX, a DOX/IND mixture or LiDOX was dispersed in the cell culture medium of the RAW264.7 cell line at various concentrations and these cells were incubated for 6, 12 or 24 h. After incubation, 10 μL of a CCK-8 solution was added to each well. After incubation for an additional 1 h at 37 ˚C, absorption of each well was measured using a microplate reader (Bio-Rad, USA) at a test wavelength of 450 nm and a reference wavelength of 690 nm. Cell viability (%) = (A_sample_)/(A_blank_) × 100 %. All experiments were performed in triplicate.

## Cytotoxicity of iDOX under different pH

The HepG-2 cell line was cultured in the DMEM medium containing 10% fetal bovine serum (FBS) at different pH values, and the initial pH value of the culture medium was regulated via the addition of HCl or NaOH. After incubation for 24 h, CCK-8 assays were employed to determine the cell viability of HepG-2 cells. All experiments were performed in triplicate.

## The effect of LiDOX on the viability of macrophages.

The Raw 264.7 cell line was cultured in the RPMI-1640 medium containing 10% FBS for 24 h. DOX, a physical mixture of DOX and IND, or LiDOX was dispersed in the medium at various concentrations. The Raw 264.7 cells were incubated for 6 h, 12 h or 24 h. After incubation, CCK-8 assays were employed to determine the cell viability of the Raw 264.7 cells. All experiments were performed in triplicate.

## The effect of LiDOX on the phagocytic activity of macrophages.

The Raw 264.7 cell line was cultured in the RPMI-1640 medium containing 10% FBS in 6-well plates (Corning, NY, USA). iDOX or LiDOX at 5 μg/mL was dispersed in the culture medium. After 24 h incubation, 100 μL of Neil Red-labeled PS nanoparticles (0.2%, 0.53 μm, Spherotech, Inc., USA) was added into each well and these nanoparticles were incubated with the cells for another 12 h at 37℃. The phagocytic efficiency of these cells to Neil Red-labeled PS nanoparticles was analyzed by flow cytometry (FACSVerse, BD Biosciences, USA).

## In vitro uptake of LiDOX by RAW 264.7 cells

RAW 264.7 cells were seeded onto confocal dish well plates at 5 × 10^4^ cells/well and treated with iDOX, LiDOX or LA+iDOX at an equivalent DOX concentration of 10 μg/mL for 6 h. In the LA+iDOX treatment group, the cells were pretreated by 10 μg/mL of laminarin before they were exposed to iDOX. Thereafter, the cells were washed with PBS three times and then fixed with 4% formaldehyde for 5 min. After washing with PBS another three times, the cell nuclei were stained with 4’, 6-diamidino-2-phenylindole (DAPI, blue) for 10 min under the protection from light. Subsequently, the cells were washed with PBS three times before they were observed under CLSM.

## Tumor-bearing mice models

The Balb/C or nude mice of 6-8 weeks (18-22 g) were used for the establishment of tumor-bearing mice models. The HepG-2 cell line or the 4T1 cell line was cultured. After harvest, 5 ×10^5^ cells were injected into the right flank of each mouse subcutaneously. Animal experiments were initiated once the average tumor volume reached 100 mm^3^.

## *In vivo* distribution of nanomedicines

### Concentration of PTX and IND in peripheral blood

PTX or PiPTX at an equivalent PTX concentration of 10 mg/kg was injected into healthy Balb/C mice through tail vein. After treatment, mice were anesthetized with pentobarbitone and 100 μL of peripheral blood was collected at 0.25, 0.5, 1, 2, 4, 6, 8, 10, and 12 h post-injection through an orbital puncture in an EDTA tube. PTX and IND were extracted by adding 1 mL of acetonitrile into the serum extracted from the blood sample. The concentration of PTX or IND was analyzed via high-performance liquid chromatography (HPLC) (Shimadzu: LC-20A; C8 column: 4.6 × 150 mm; mobile phase A: water with 0.02% acetic acid, 45%; mobile phase B: acetonitrile, 55%; flow rate: 1 mL/min; and analysis time: 15 min).

### Concentration of PTX and IND in the tumor and small intestine

PTX or PiPTX at an equivalent PTX concentration of 10 mg/kg was injected into HepG-2 tumor bearing-mice through tail vein. The mice were euthanasia with pentobarbitone at 8 h after injection. The tumor tissue and the small intestine were excised, weighed, and ground at 8 h after injection. PTX and IND were extracted by adding 1 mL of acetonitrile into the ground tissues, and the concentration of PTX or IND was analyzed via HPLC.

### In vivo distribution of iDOX and LiDOX

The Balb/C mice bearing 4T1 tumors were established and treated with DOX in various dosage forms at an equivalent DOX concentration of 5 mg/kg or saline every 4 days. After 6 treatments, mice were euthanasia with pentobarbitone and the tumor tissue and main organs of each mouse were isolated and were observed under an in vivo multispectral imaging system (IVIS Spectrum, PerkinElmer, USA). The averaged fluorescent intensity of each organ or tissue was calculated for semi-quantitative biodistribution analysis. The tumor tissue was fixed, sectioned, labeled with an FITC-CD68 antibody and observed under CLSM.

The Balb/C mice bearing 4T1 tumors were established and treated with DOX in various dosage forms at an equivalent DOX concentration of 5 mg/kg or saline. Mice were anesthetized with pentobarbitone and the peripheral blood from each mouse was collected in a heparin-containing tube 2 h after injection. Red blood cells were lysed using a red blood cell lysing buffer (Sigma, USA). The cells suspensions were stained for 30 min at 4˚C with a FITC-CD11b antibody and a PE-Ly6C antibody. The distribution of DOX in cells labeled with different antibodies was analyzed via flowcytometry (FACSVerse, BD Biosciences USA).

## *In vivo* anti-tumor effects

### Treatment regimen

PTX, PiPTX, a mixture of PTX and IND (PTX+IND), or saline was injected intravenously into HepG-2 tumor-bearing mice every 4 days for 4 times at an equivalent dosage of 10 mg PTX /kg to assess the anti-tumor activity of PTX in various dosage forms. DOX, a mixture of DOX and IND (DOX+IND), iDOX, LiDOX or saline was injected intravenously into HepG-2 tumor-bearing mice every 4 days at an equivalent dosage of 5 mg DOX/kg for 12 days to assess the anti-tumor activity of iDOX or LiDOX. The exact treatment procedure for these DOX dosage forms was applied to a 4T1 tumor-bearing mice model, but the treatment duration was extended to 20 days. To compare the anti-tumor effect of iDOX with LipoDOX® (a commercial nanomedicine of DOX carried by liposomes), DOX, DOX+IND, iDOX, LipoDOX® or saline was injected intravenously into Balb/C mice bearing 4T1 tumors every 4 days at an equivalent DOX dosage of 3 mg/kg for 16 days. To compare the mortality of iDOX with LipoDOX, DOX, a mixture of DOX and IND (DOX+IND), iDOX, LipoDOX or saline was injected intravenously into Balb/C mice bearing 4T1 tumors through tail vein every 4 days at an equivalent DOX dosage of 5 mg/kg for 20 days. The tumor size, the body weight, and the survival rate of each mouse were recorded during the treatment course. The tumor volume = width^2^ × length/2. Upon completion of the above treatment procedures, the mice were euthanasia with pentobarbitone, and tumor tissues and main organs were collected.

### Assessment of biosafety

The liver, heart, spleen, lung, kidney, and small intestine tissues were excised after treatment with various dosage forms of PTX or DOX. These organs/tissues were fixed in the O.C.T (optimal cutting temperature compound) for 24 h for histology investigation. The fixed organs/tissues were cut into sections at a thickness of 10 µm and the sections were stained by hematoxylin & eosin according to the standard protocol and observed under a microscope. The body weight was measured at each treatment and the behaviors of each mouse were monitored for any signs of illness during the experiment process.

### Levels of proliferation and apoptosis within tumor tissues.

The tumor tissue collected from each mouse after *in vivo* treatment by various dosage forms of PTX or DOX was fixed in 4% paraformaldehyde in PBS (4˚C, overnight). The tissue was sectioned for fluorescent labeling. The TUNEL/Ki67 assay of the tumor tissue from the mice treated with PTX, PTX+IND, PiPTX or saline was conducted using a TUNEL/Ki67 detection kit according to the manufacturer’s instructions. The TUNEL and Caspase-3 assay of the tumor tissue from the mice treated with DOX, DOX+IND, iDOX, LiDOX or saline was conducted using a TUNEL and Caspase-3 detection kit according to the manufacturer’s instruction, respectively. The stained samples were observed under a confocal laser scanning microscope (CLSM) (Carl Zeiss LSM880, Germany).

## Immune activation by nanomedicines.

### Modulation towards macrophage cells.

The human monocytic THP-1 cell line or the RAW 264.7 cell line was incubated in the RPMI-1640 culture medium containing 10% of FBS and antibiotics including penicillin (100 IU/mL) and streptomycin (100 mg/L). Macrophages were polarized to the M1 or M2 phenotype by stimulating the cell line with 20 ng/mL IFN-γ + 100 ng/mL LPS for 24 h or 20 ng/mL IL-4 + 20 ng/mL IL-13 for 48 h, respectively. The M1 or M2 macrophages were treated with different dosage forms of PTX or DOX at various concentrations for additional 48 h. The secreted concentrations of IL-6 and CCL-18 in the medium were detected by ELISA assays using ELISA kits following the manufacture’ instructions. Stimulated macrophages were seeded into an upper chamber and the 4T1 cell line into a lower chamber in a Transwell system. After co-incubation for 48 h, the cell viability of 4T1 cells in the lower chamber was detected using the CCK-8 assay to determine the cytotoxicity of macrophages after stimulation by DOX, iDOX or LiDOX.

### Modulation towards TAMs.

The 4T1 tumor-bearing mice model was established as described above. Mice were euthanasia with pentobarbitone when the average tumor volume reached around 100 mm^3^. The tumor tissue was surgically resected, incubated with trypsin at 37˚C for 60 min, ground and filtered through a 70-mesh cell sieve to obtain a single-cell suspension of the tumor tissue. These cells were incubated with different dosage forms of PTX or DOX for additional 48 h. After incubation, the cells were labeled with an FITC-CD68-FITC antibody and an APC-CD206 or APC-CD86 antibody and analyzed via flowcytometry (FACSVerse, BD Biosciences USA).

### Modulation towards bone marrow-derived macrophages.

After the C57BL/6 mice were euthanasia with pentobarbitone, the cells in the tibia and femur cavity were collected and cultured in the RPMI-1640 medium. They were activated by 10 ng/mL of Macrophage Colony-Stimulating Factor (M-CSF) for 72 h to obtain bone marrow-derived macrophages (BMDMs). The spent medium was replaced with a fresh medium containing 100 ng/mL of IL-4. After 72-h cultivation, myeloid-derived suppressor cells (MDSCs) were obtained. The MDSCc were treated with different dosage forms of PTX at an equivalent PTX concentration of 10 μg/mL for another 72 h. At the end of co-incubation, the cells were collected and labeled with an FITC-Ly6C antibody and an APC-Gr-1 antibody, and analyzed via flow cytometry (FACSVerse, BD Biosciences USA).

### Analysis of the tumor site immune environment

The ELISA assay was employed to investigate the cytokine level of IL-1β, IL-10, TGF-β and TNF-α in the tumor tissue after treatment with different dosage forms of PTX or DOX using ELISA kits under the guidance of manufacturer’ instructions. To analyze the distribution of immune cells, tumor tissues were collected after *in vivo* treatment by various dosage forms of PTX or DOX and fixed in 4% paraformaldehyde in PBS (4˚C, overnight) for fluorescence labeling. The fixed tissues were frozen-sectioned and labeled with an FITC-CD68 antibody/an APC-CD86 antibody or an FITC-CD68 antibody/an APC-CD206 antibody, and they were observed under CLSM.

# Supplementary Figures


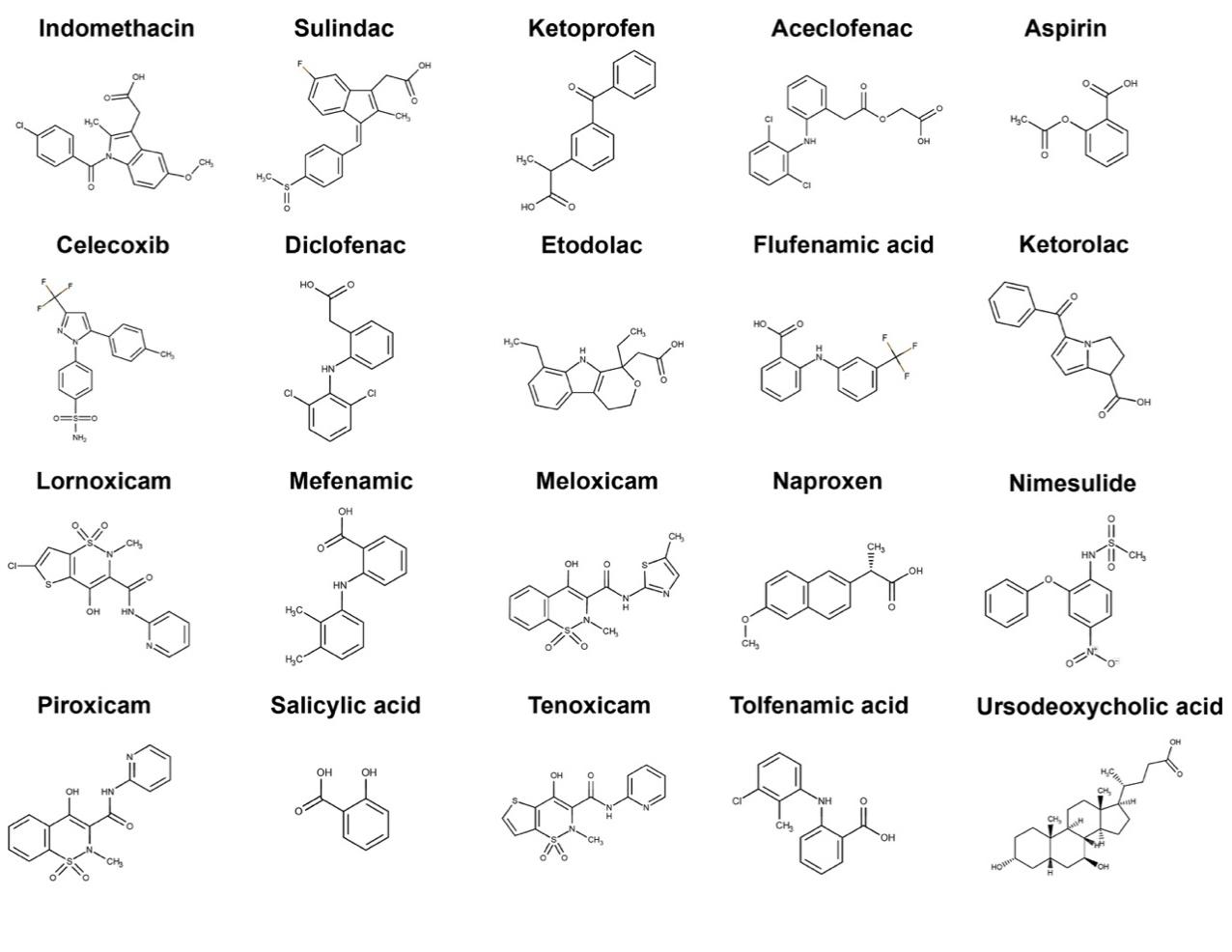


**Figure S1.** Molecular structures of FDA-approved NSAIDs


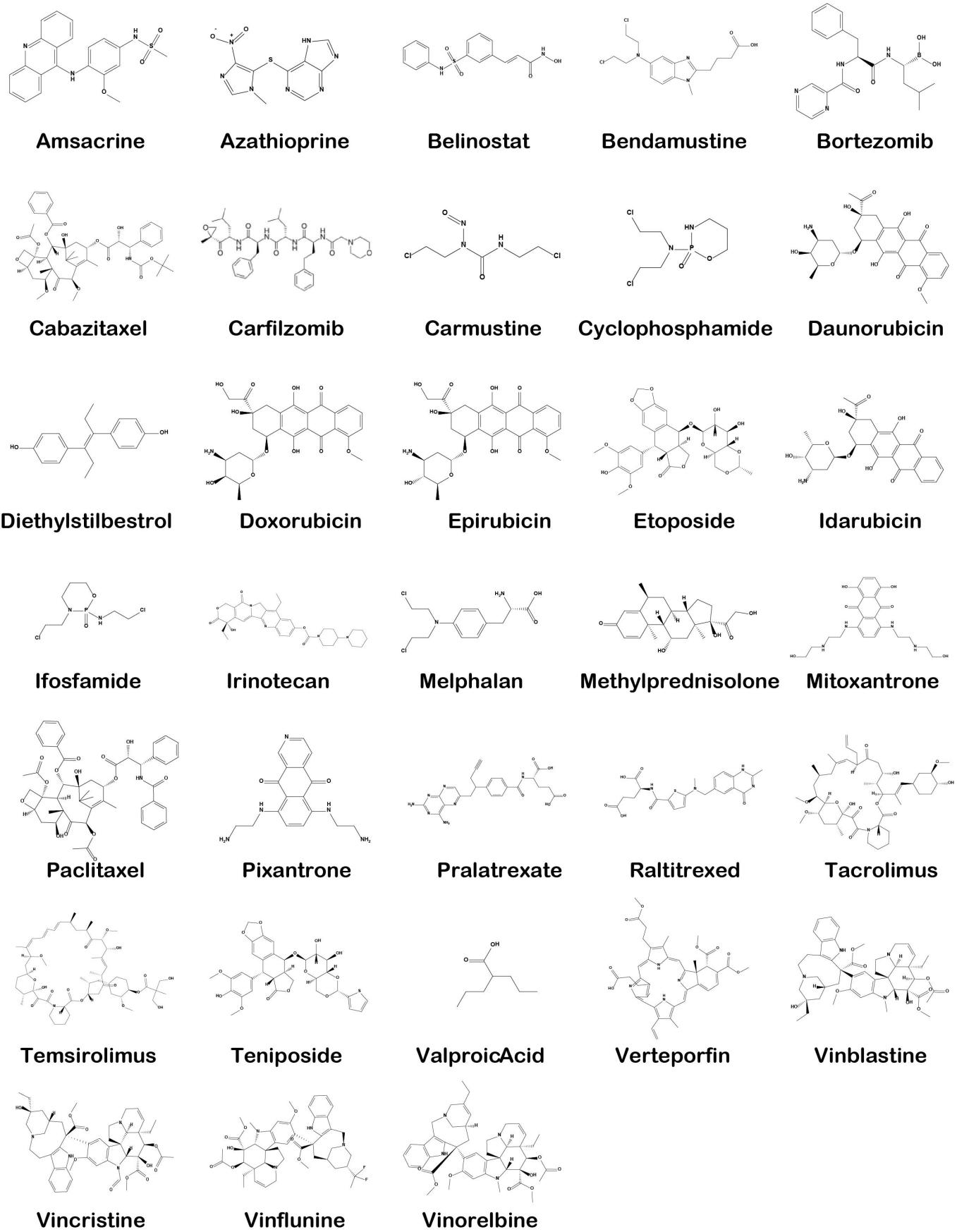


**Figure S2.** Molecular structures of FDA-approved antineoplastic drugs.


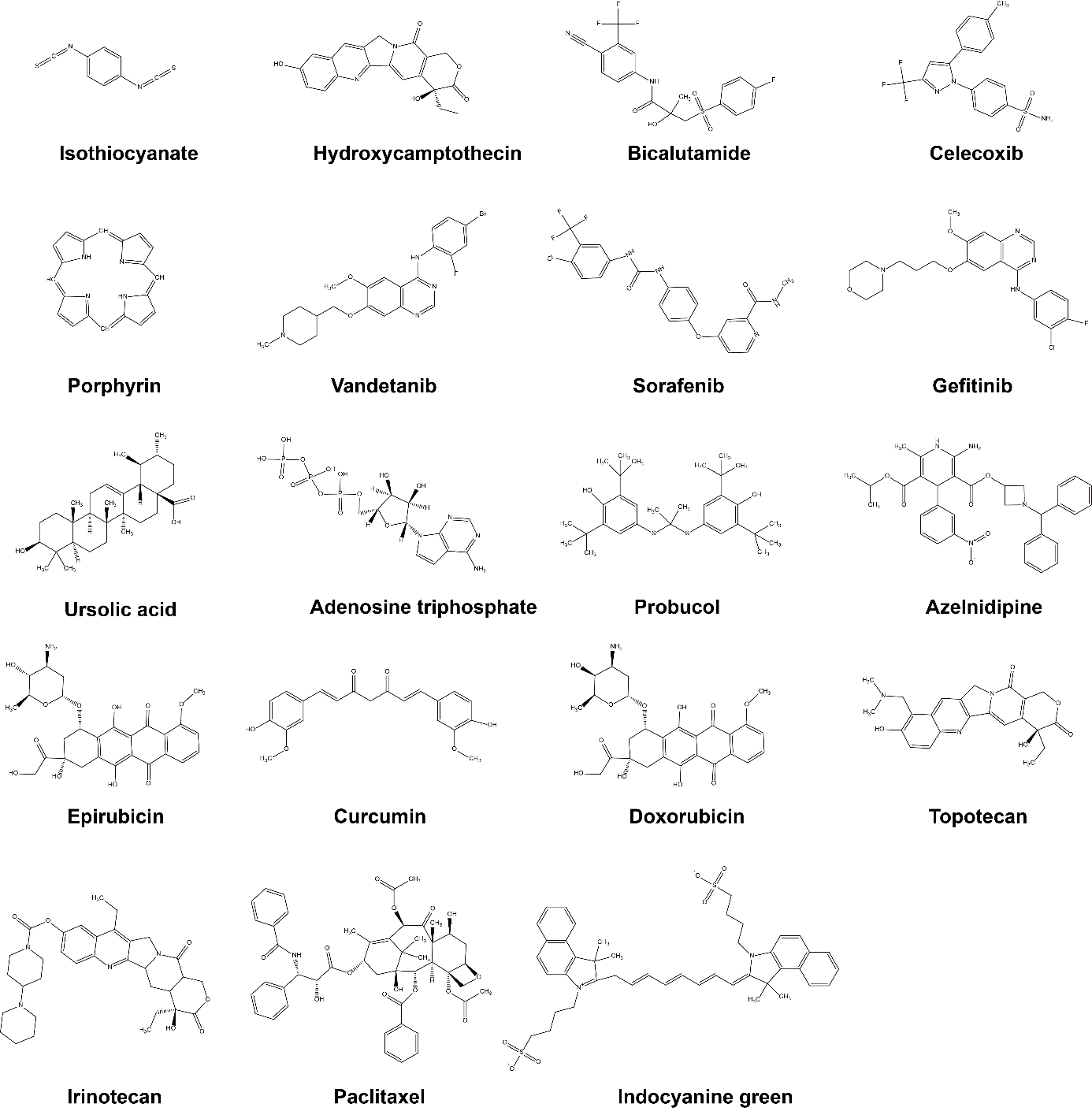


**Figure S3.** Molecular structures of drugs which have been reported to be capable of forming nano-assemblies.


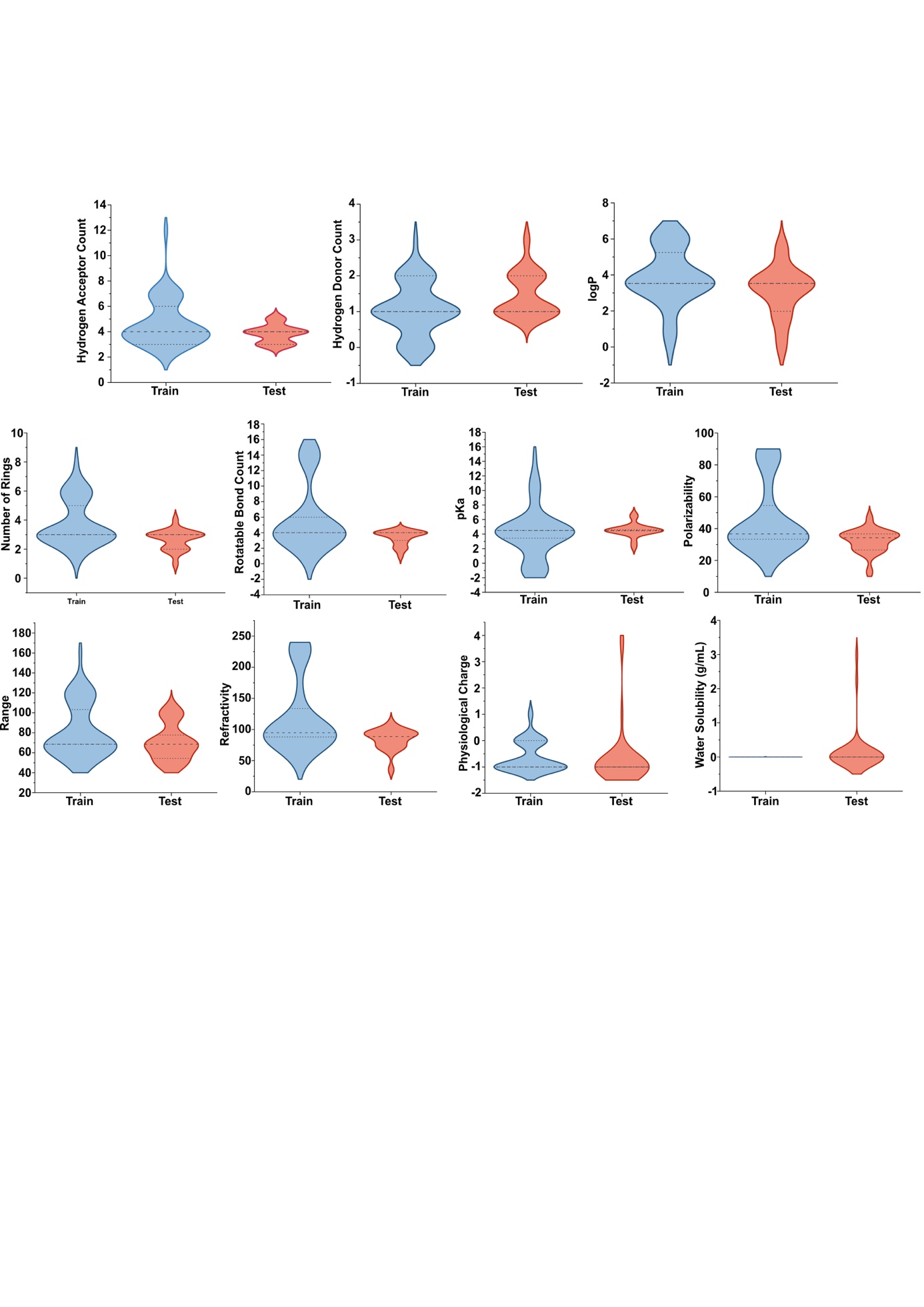


**Figure S4.** Comparison of drug parameters of the drug A in the training and test dataset. The dotted lines represent the median values.


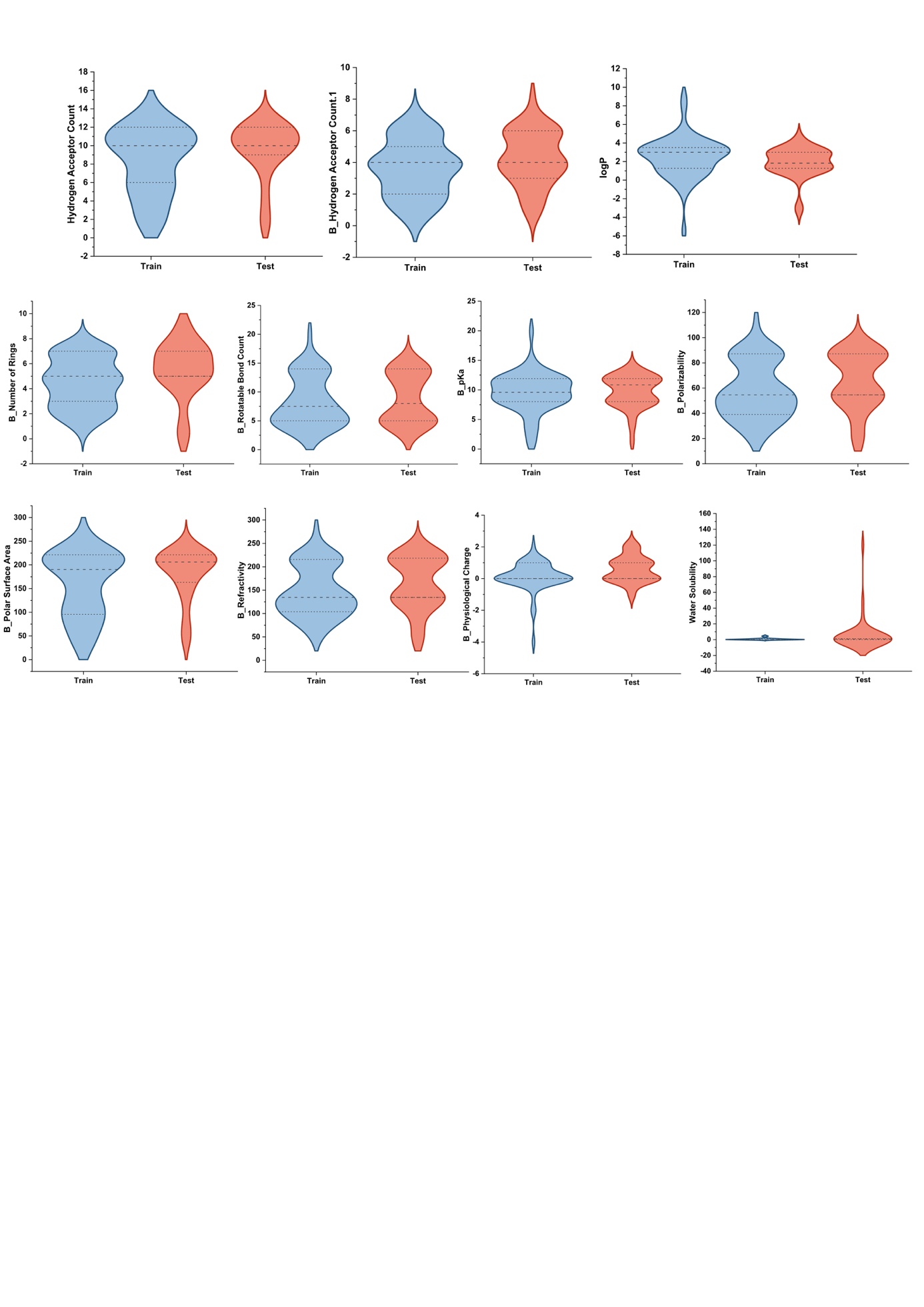


**Figure S5.** Comparison of drug parameters of the drug B in the training and test dataset. The dotted lines represent the median values.


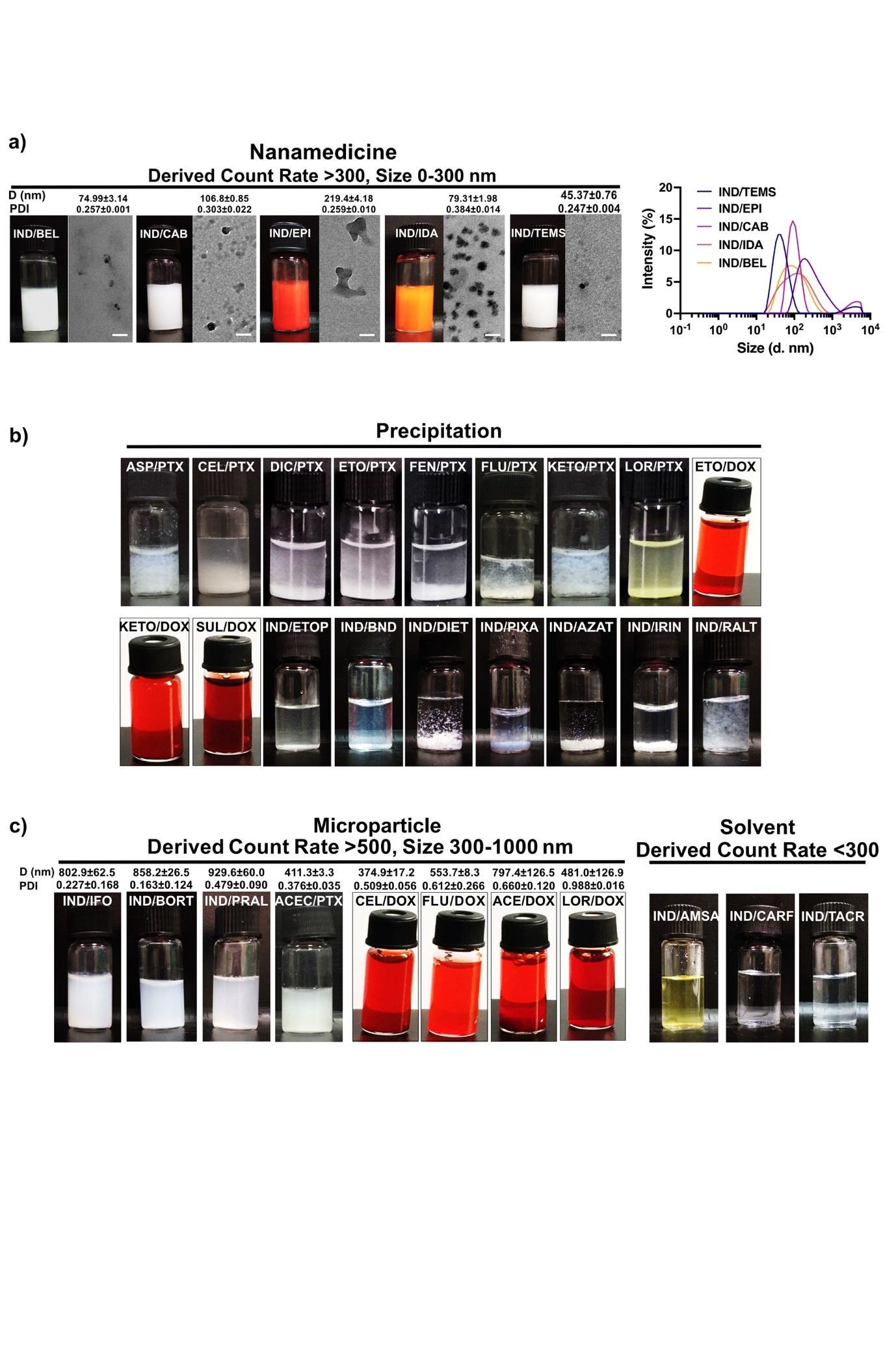


**Figure S6.** Observations of a dispersion system after combination of a drug A and a drug B in the training set in an aqueous phase. a) Digital pictures, TEM images and size distributions of nanomedicines constructed by drug combination in the training set. Scale bar = 100 nm; b) Digital pictures of precipitations from drug combination in the training set; c) Digital pictures and size distributions of microparticles or solvents from drug combination in the training set.


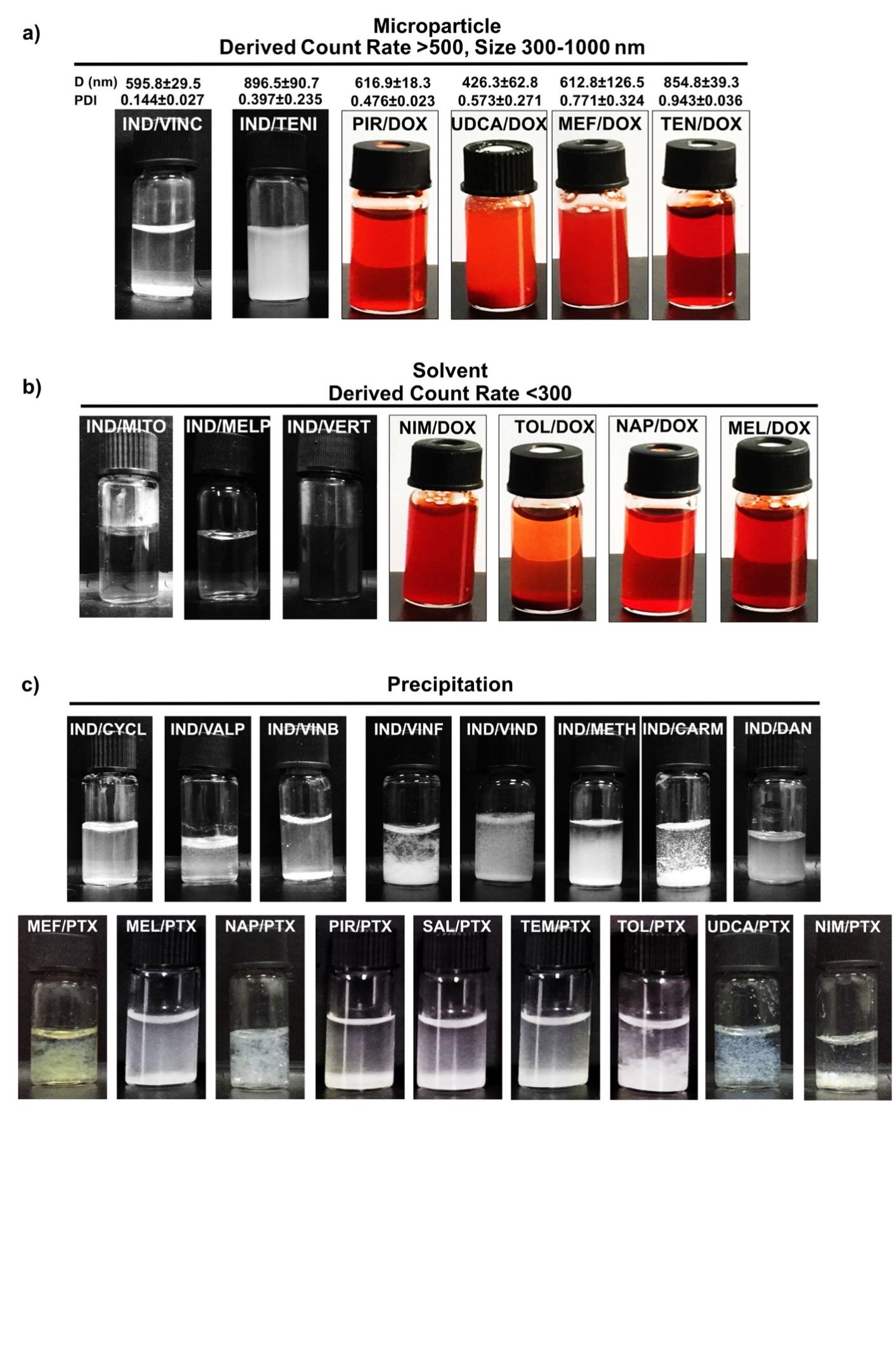
**Figure S7**. Observations of a dispersion system after combination of a drug A and a drug B in the testing set in an aqueous phase. a) Digital pictures and size distributions of microparticles from drug combination in the testing set; b) Digital pictures of solvents from drug combination in the testing set; c) Digital pictures of precipitations from drug combination in the testing set.


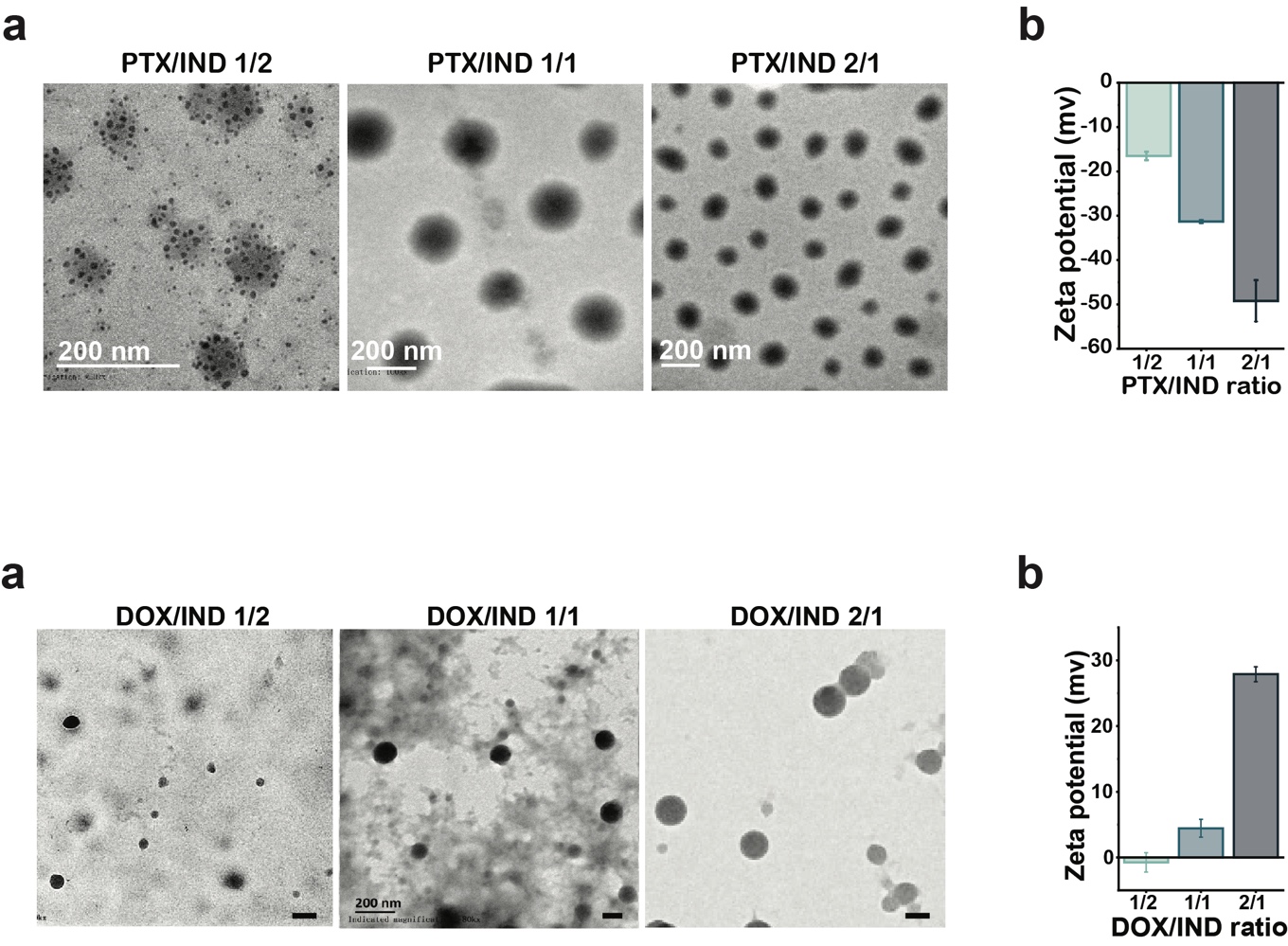


**Figure S8.** Representative TEM images (a) and zeta potentials (b) of the PTX/IND nanomedicine (iPTX) at various feeding ratios. Scale bar =200 nm.


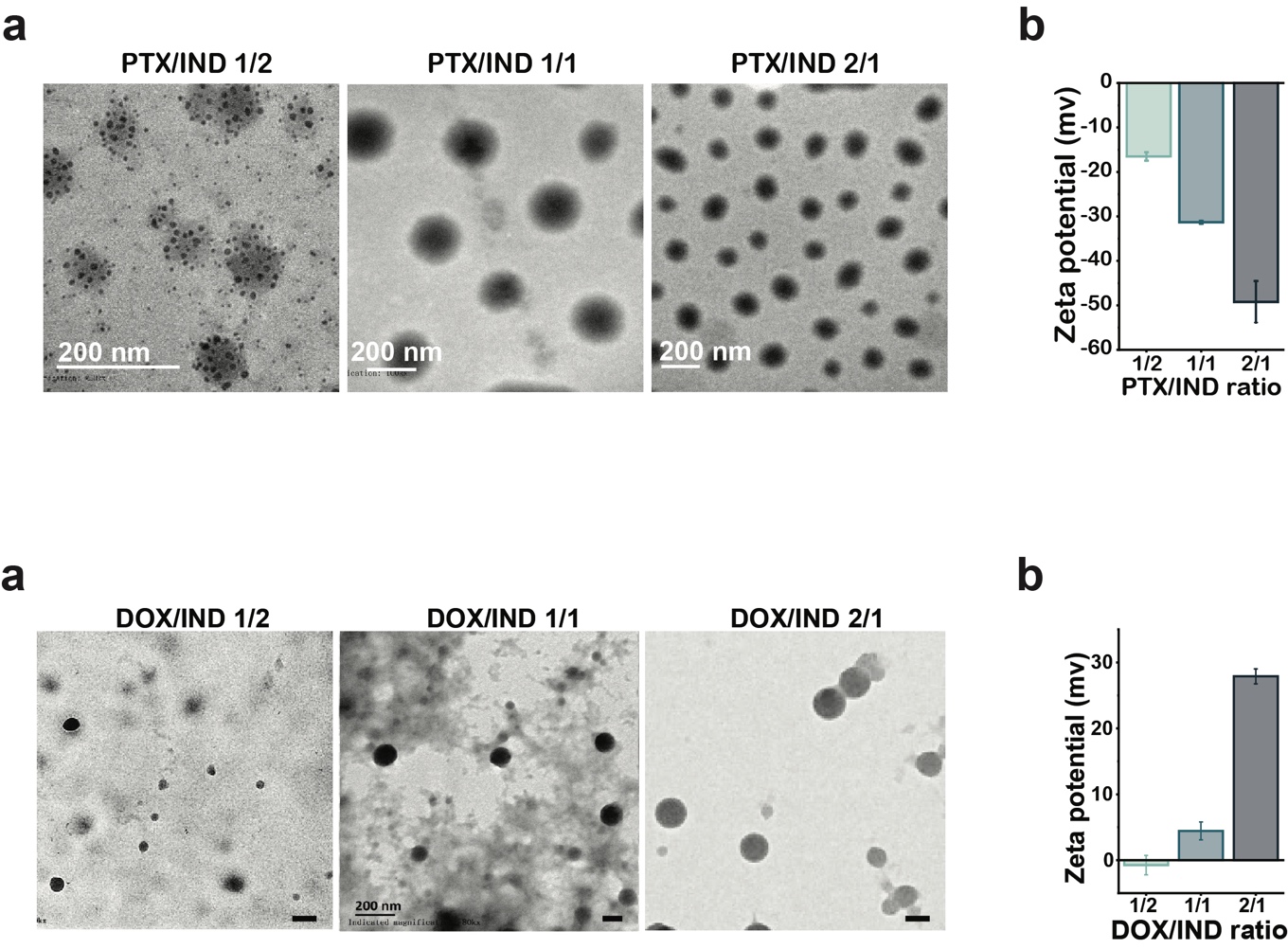


**Figure S9.** Representative TEM images (a) and zeta potentials (b) of the DOX/IND nanomedicine (iDOX) at various feeding ratios. The data in the figures is presented as the Mean ± SD, n=3. Scale bar =100 nm.


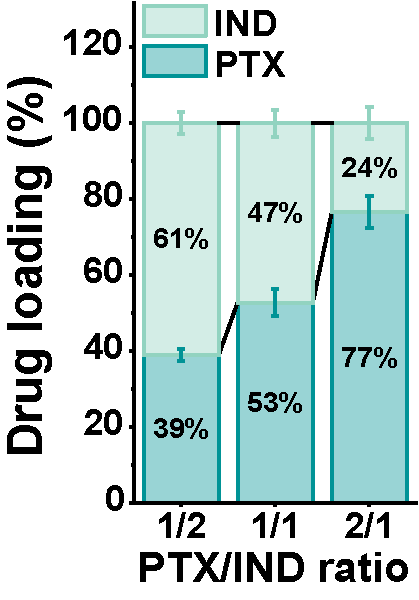


**Figure S10.** Drug loading of PTX and IND in the PTX/IND nanomedicine at different feeding ratios. The data in the figures is presented as the Mean ± SD, n=3


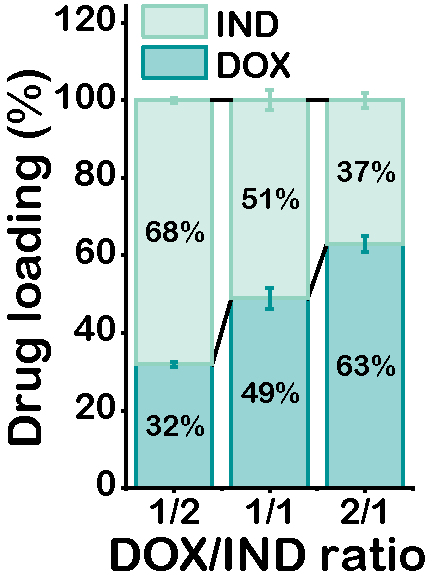


**Figure S11.** Drug loading efficiencies of the DOX/IND nanomedicine at different DOX/IND feeding ratios. The data in the figures is presented as the Mean ± SD, n=3


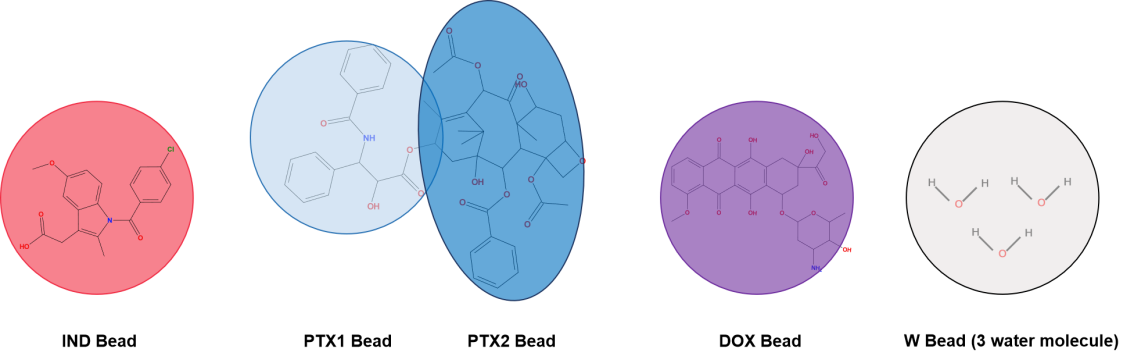


**Figure S12.** Beads settings in the DPD simulation.


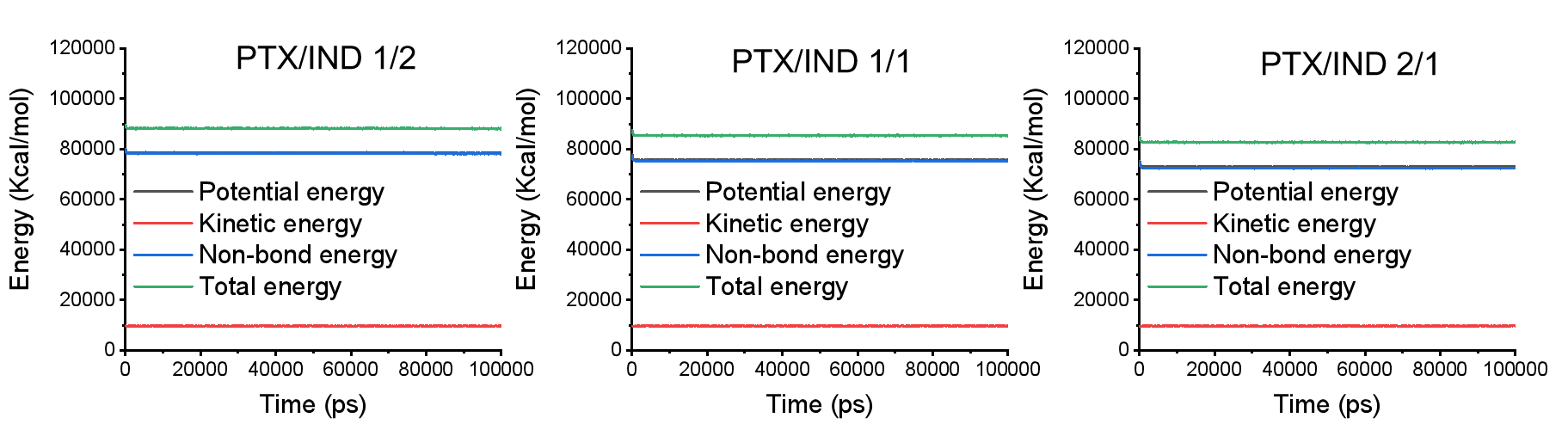


**Figure S13.** Changes in the dynamic energies in the 100-ns DPD simulation of the PTX/IND nanomedicine at various feeding ratios.


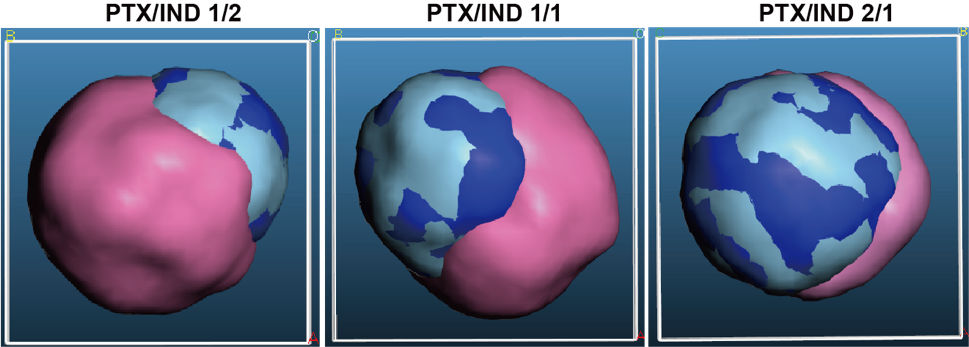


**Figure S14.** Mesostructures of the PTX/IND nanomedicine at different feeding ratios after 100-ns DPD simulation.


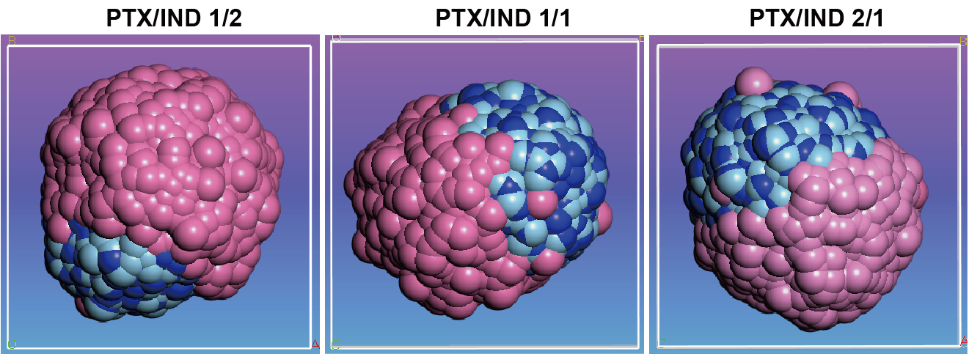


**Figure S15.** Molecular distribution in the PTX/IND nanomedicine at different feeding ratios. IND molecules are shown as light pink spherical particles, and PTX molecules as light blue/blue spherical particles.


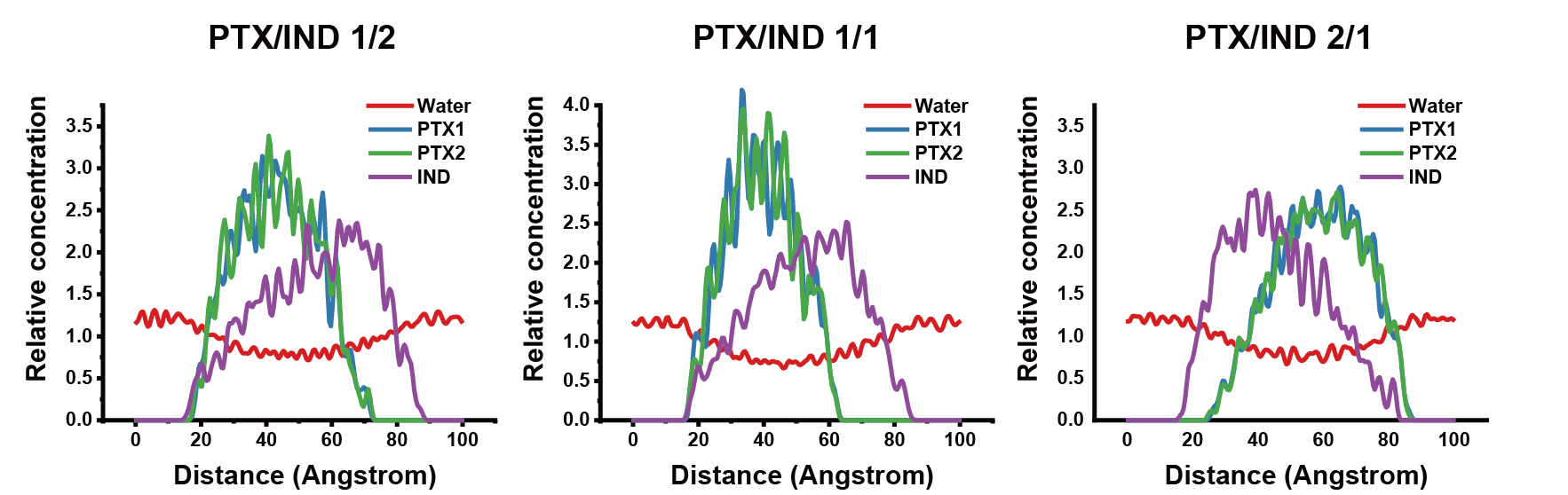


**Figure S16.** Concentration profiles of molecules in the PTX/IND nanomedicine at different feeding ratios after 100-ns DPD simulation. PTX1 and PTX2 represent different parts of the PTX molecule.


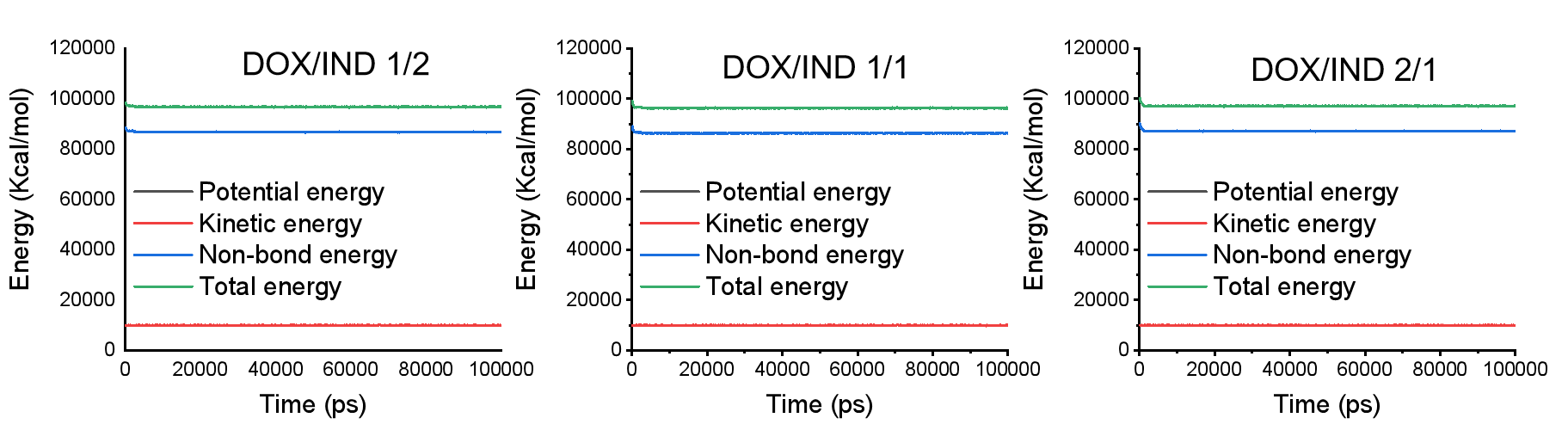


**Figure S17.** Changes in the dynamic energies from the 100-ns DPD simulation of the DOX/IND nanomedicine at various feeding ratios.


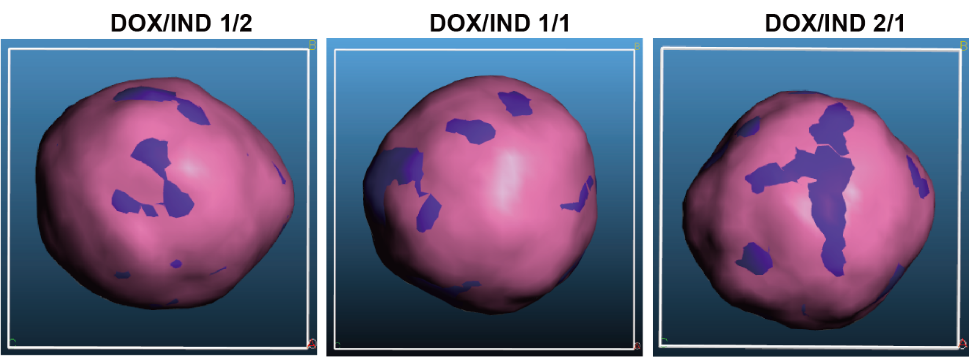


**Figure S18.** Mesostructures of DOX, IND or water beads in the DOX/IND nanomedicine at various DOX/IND feeding ratios after 100-ns DPD simulation.


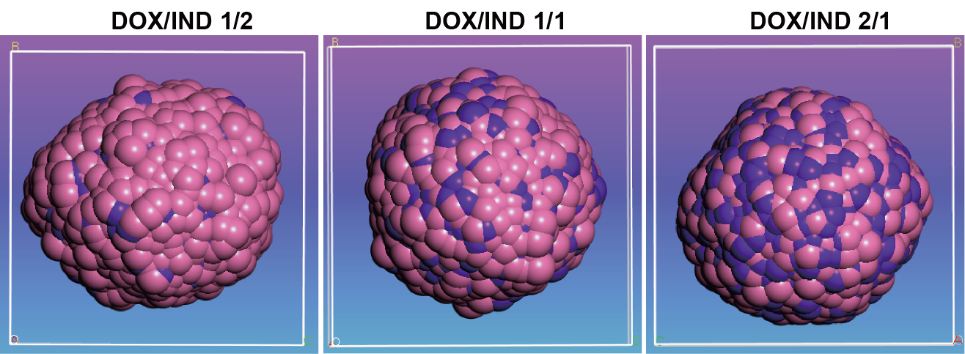


**Figure S19.** Molecular distribution in the DOX/IND nanomedicine at different feeding ratios. The IND molecules are shown as light pink spherical particles and DOX molecules as purple spherical particles.


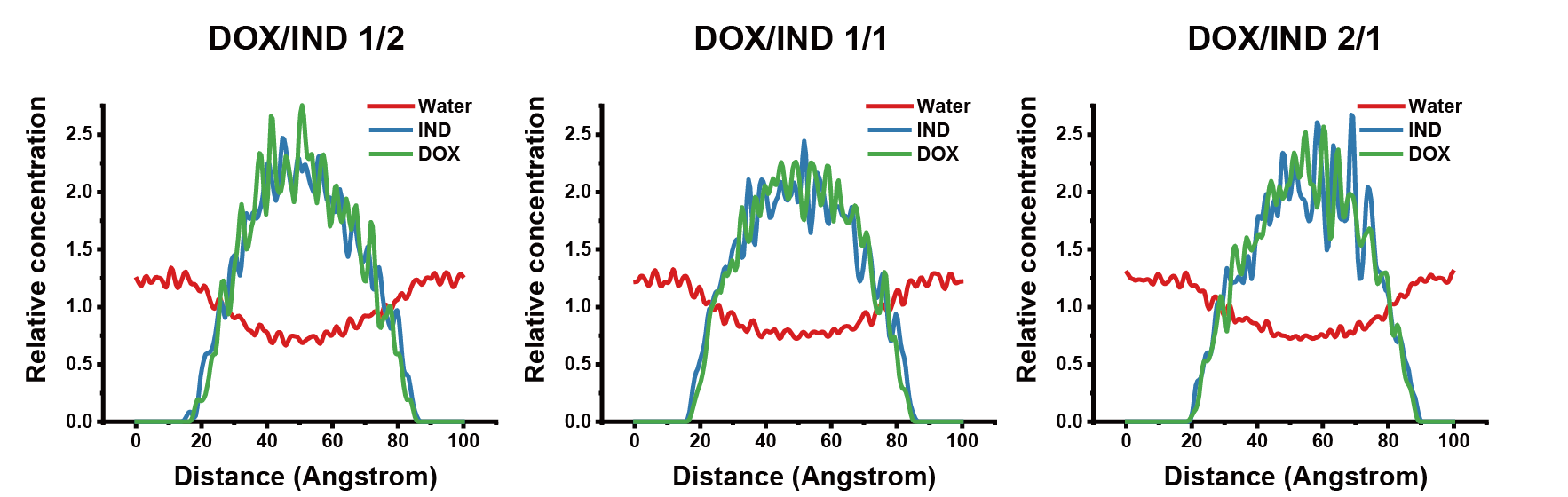


**Figure S20.** Concentration profiles of DOX, IND or water beads in the DOX/IND nanomedicine at various DOX/IND feeding ratios after 100-ns DPD simulation.


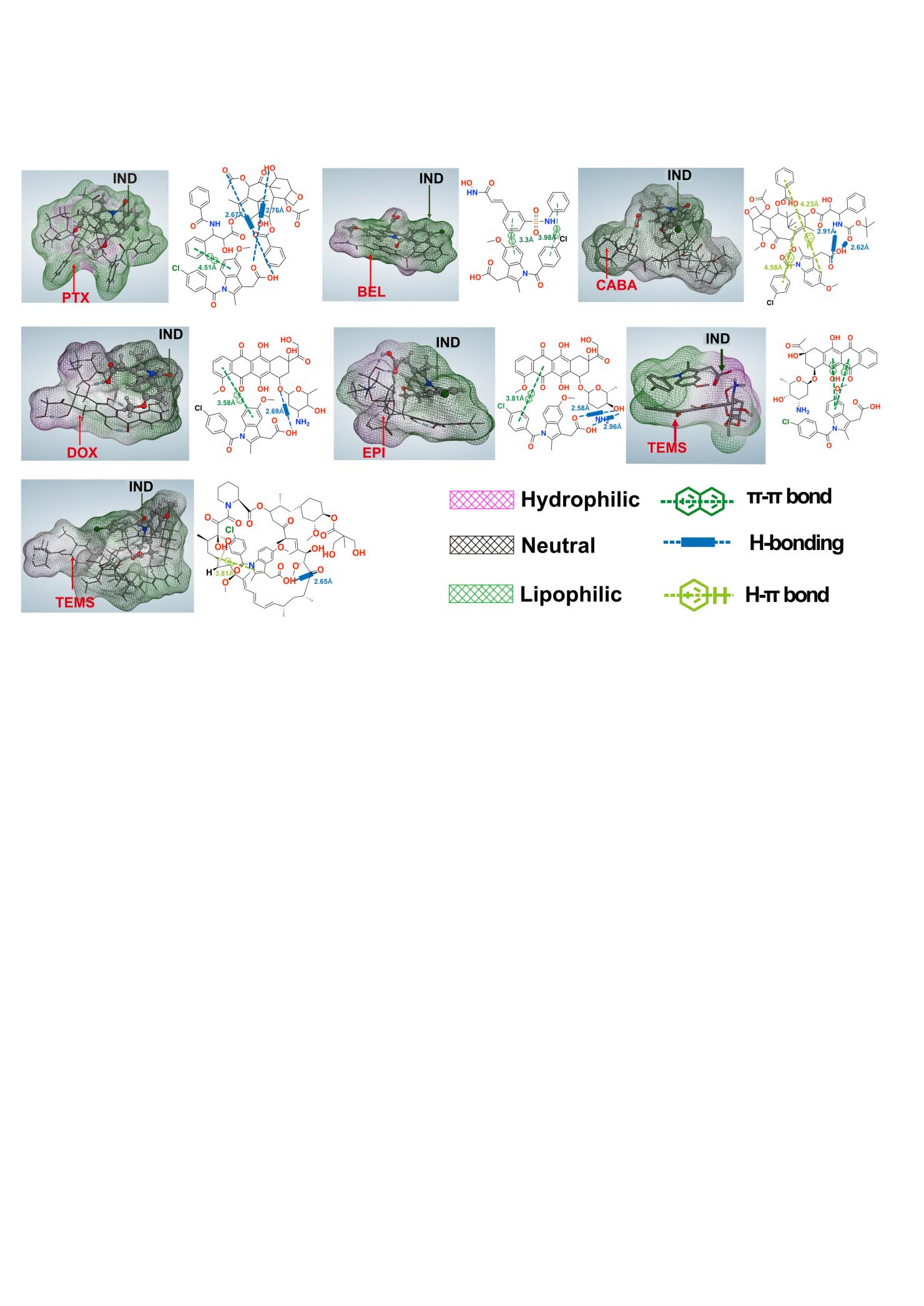


**Figure S21.** Renderings of hydrophilic and hydrophobic regions in individual molecules and schematic diagrams of intermolecular forces in the IND/an antineoplastic drug nanomedicine after 100-ns MD simulation. The hydrophilic area is presented in pink, the lipophilic area in green, and the neutral area in transparent white. The intermolecular hydrogen bonding and π-π stacking are shown in the molecular structures. The hydrogen bond is represented by a blue dotted line with a blue box in the middle, the π-π stacking by a green dotted line with double green hexagons in the middle, and the π-hydrogen bond by a green dotted line with H and hexagon symbols in the middle.


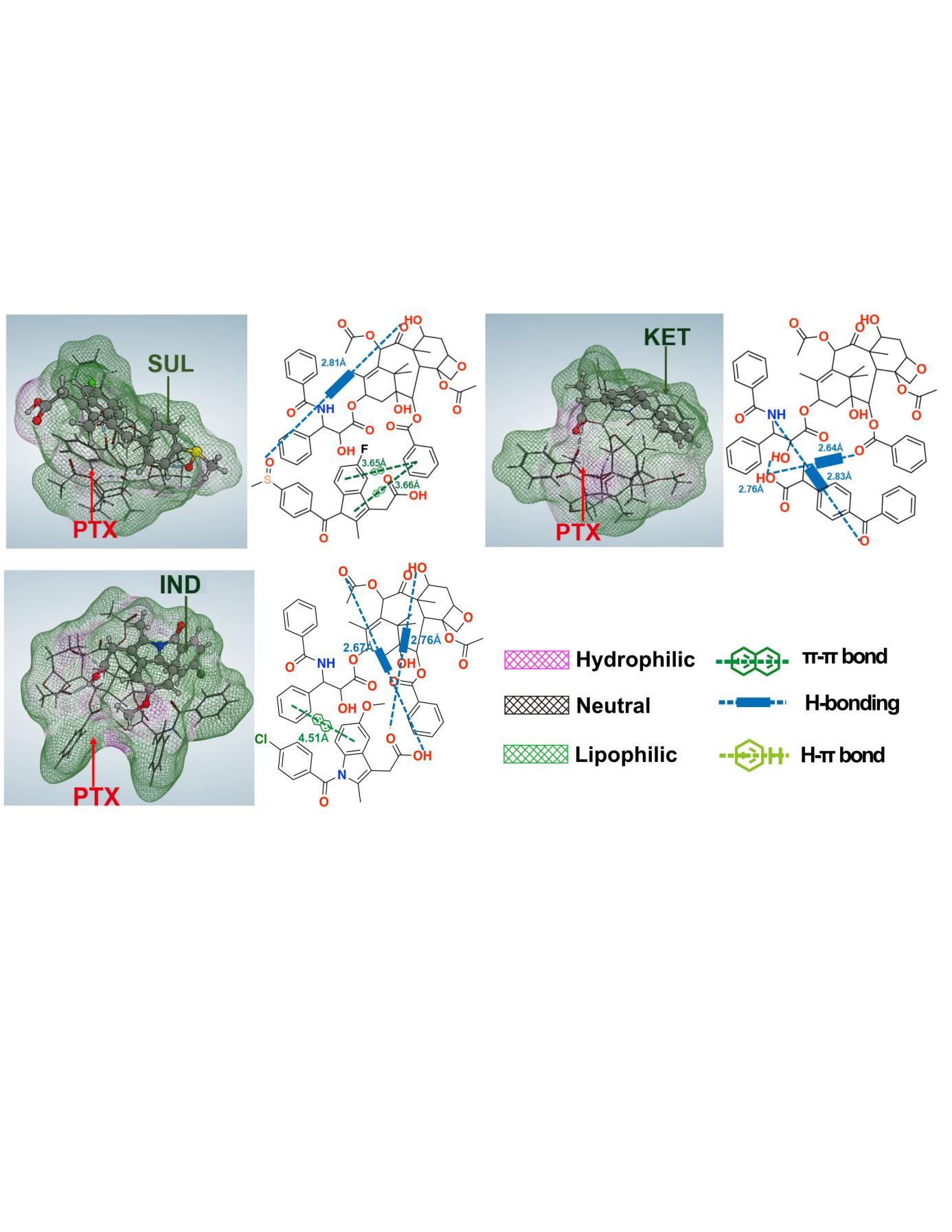


**Figure S22.** Renderings of hydrophilic and hydrophobic regions in individual molecules and schematic diagrams of intermolecular forces in the PTX/an NSAID nanomedicine after 100-ns MD simulation. The hydrophilic area is presented in pink, the lipophilic area in green, and the neutral area in transparent white. The intermolecular hydrogen bonding and π-π stacking are shown in the molecular structures. The hydrogen bond is represented by a blue dotted line with a blue box in the middle, the π-π stacking by a green dotted line with double green hexagons in the middle, and the π-hydrogen bond by a green dotted line with H and hexagon symbols in the middle.


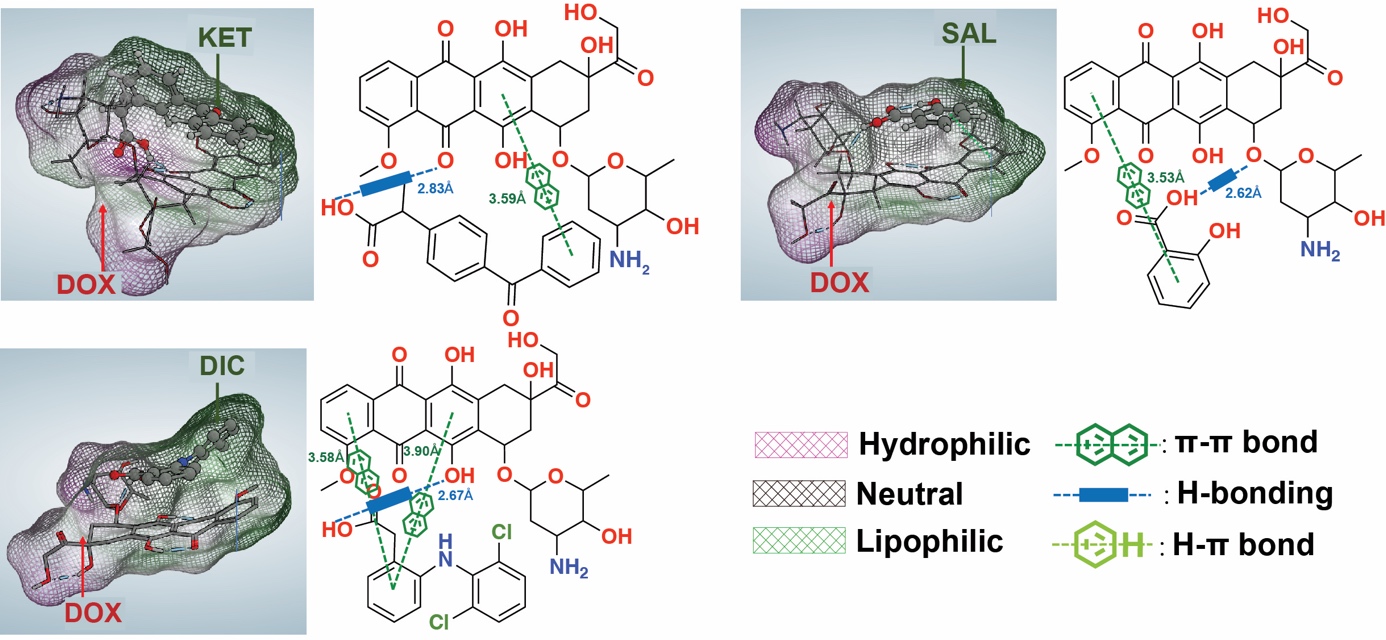


**Figure S23.** Rendering of hydrophilic region, lipophilic region and intermolecular forces distribution of DOX/NSAIDs nanomedicines after 100 ns MD simulation, the hydrophilic area is presented as pink grid, the lipophilic area is presented as green grid, the neutral area is presented as transparent white, intermolecular hydrogen bonding and π-π stacking is indicated in the molecular structure formula, and the hydrogen bond is represented by blue dotted line with blue box in the middle, π-π stacking is represented by green dotted line with double green hexagons in the middle, π-hydrogen bond is represented by green dotted line with H and hexagon symbol in the middle.


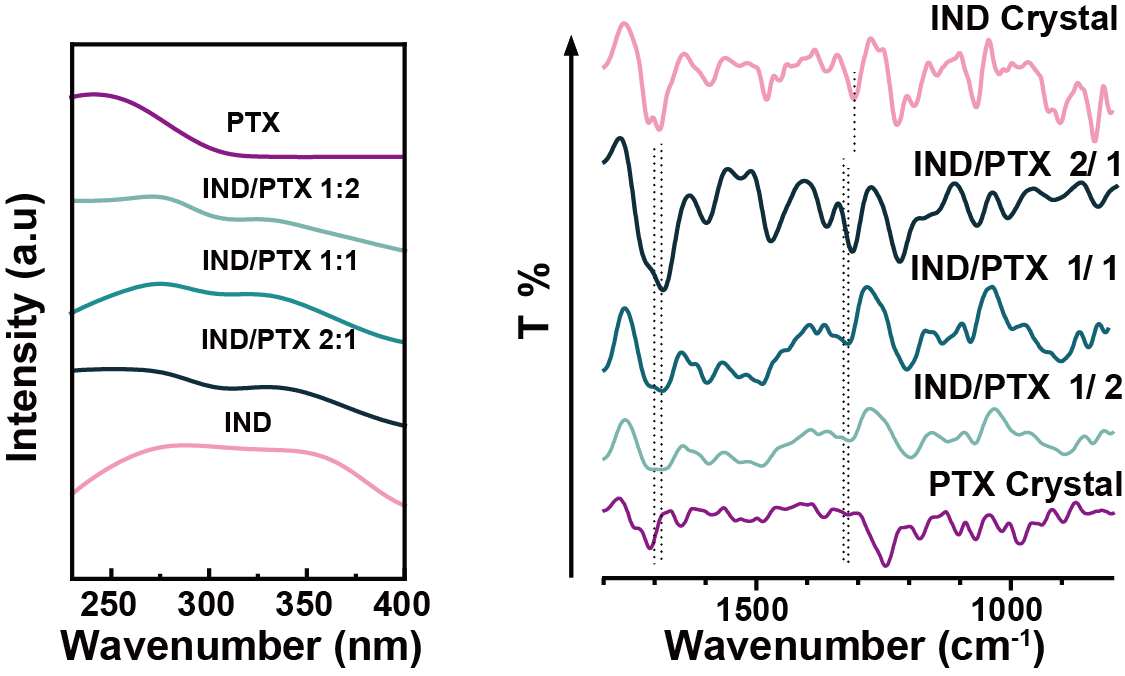


**Figure S24.** Ultraviolet (Left) and infrared spectra (Right) of the PTX/IND nanomedicine at different feeding ratios.


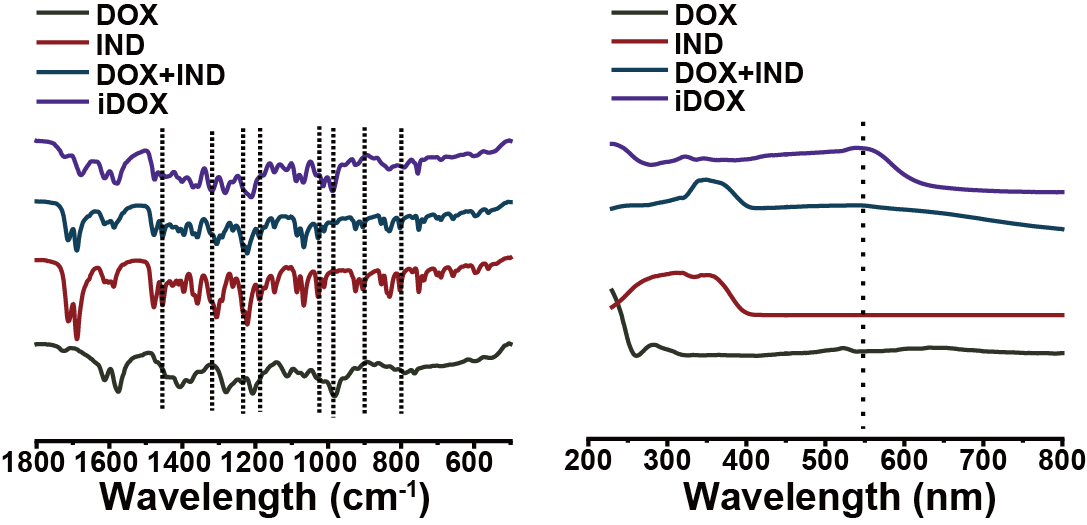


**Figure S25.** FTIR (Left) and UV spectra (Right) of the DOX/IND nanomedicine. The changes in the peak shape of UV and IR spectra indicate that π-π stacking and hydrogen bonding are formed between DOX and IND.


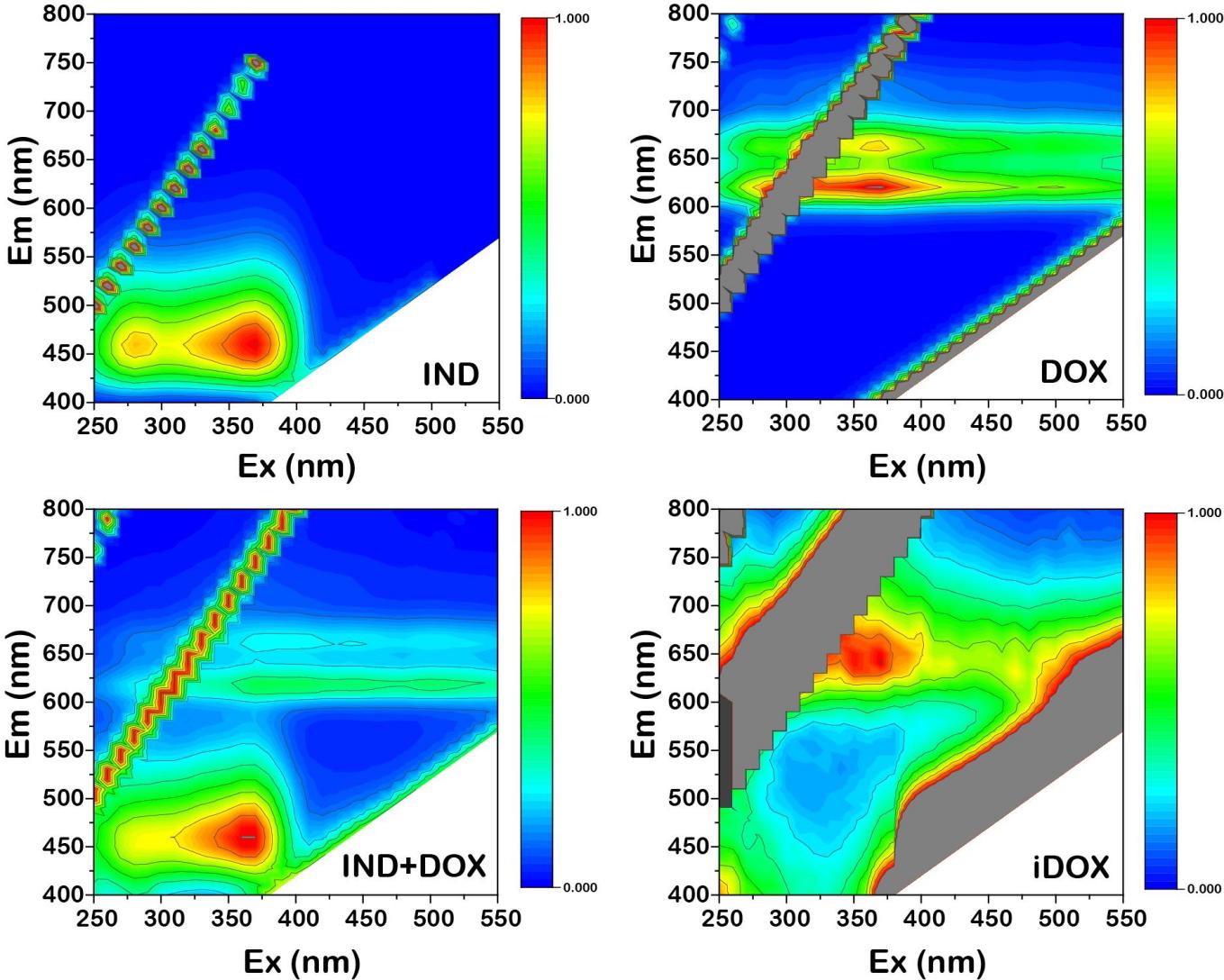


**Figure S26.** Solid fluorescence spectra of IND, DOX, a mixture of IND and DOX (IND+DOX), and iDOX.


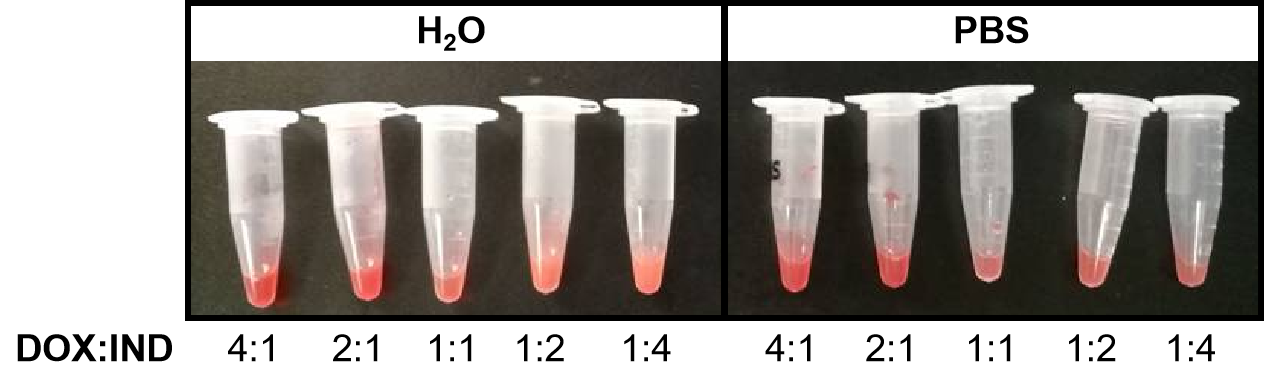


**Figure S27.** Digital photos of the DOX/IND nanomedicine at various DOX/IND feeding ratios after they were dispersed in water or PBS for 24 h.


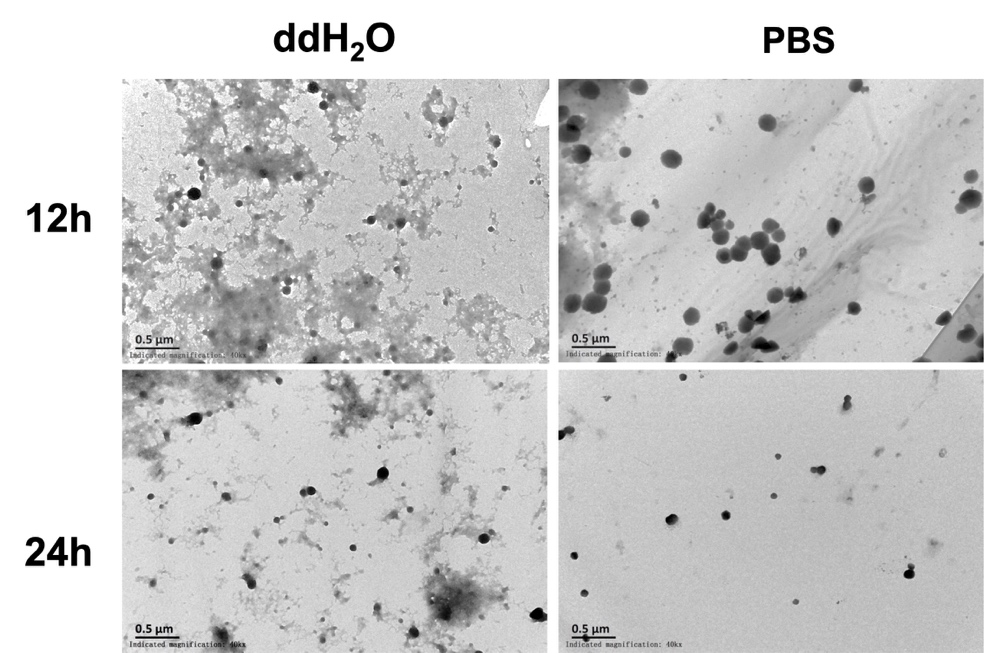


**Figure S28.** TEM images of iDOX dispersed in ddH_2_O and PBS at 12 h and 24 h. Scale bar = 500 nm.


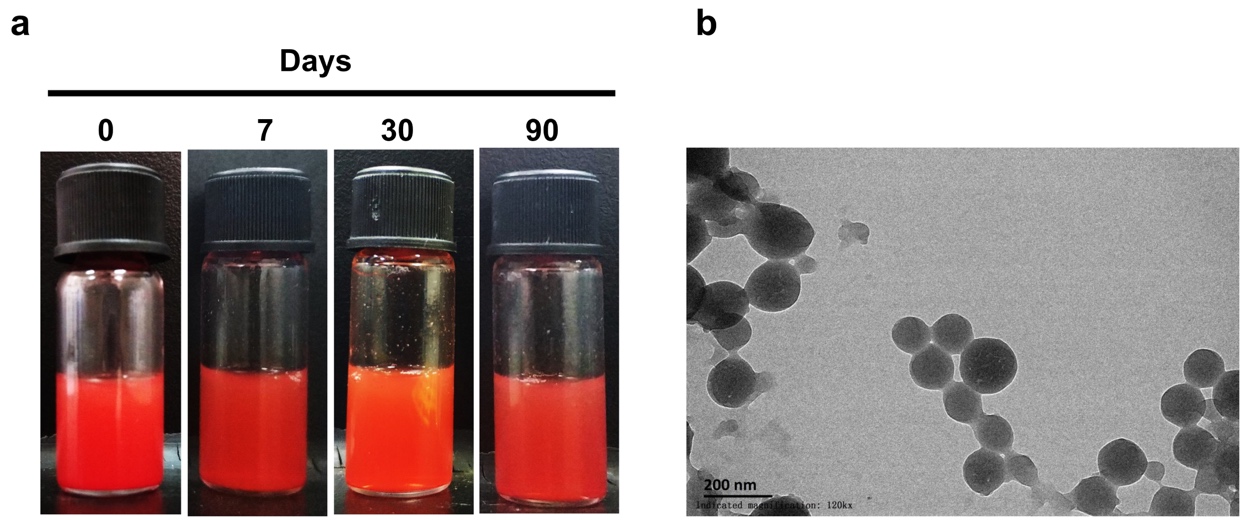


**Figure S29**. A. Digital photos of iDOX after 0, 7, 30, 90 days’ storage. TEM image of iDOX after 90 days’ storage. Scale bar = 200 nm.


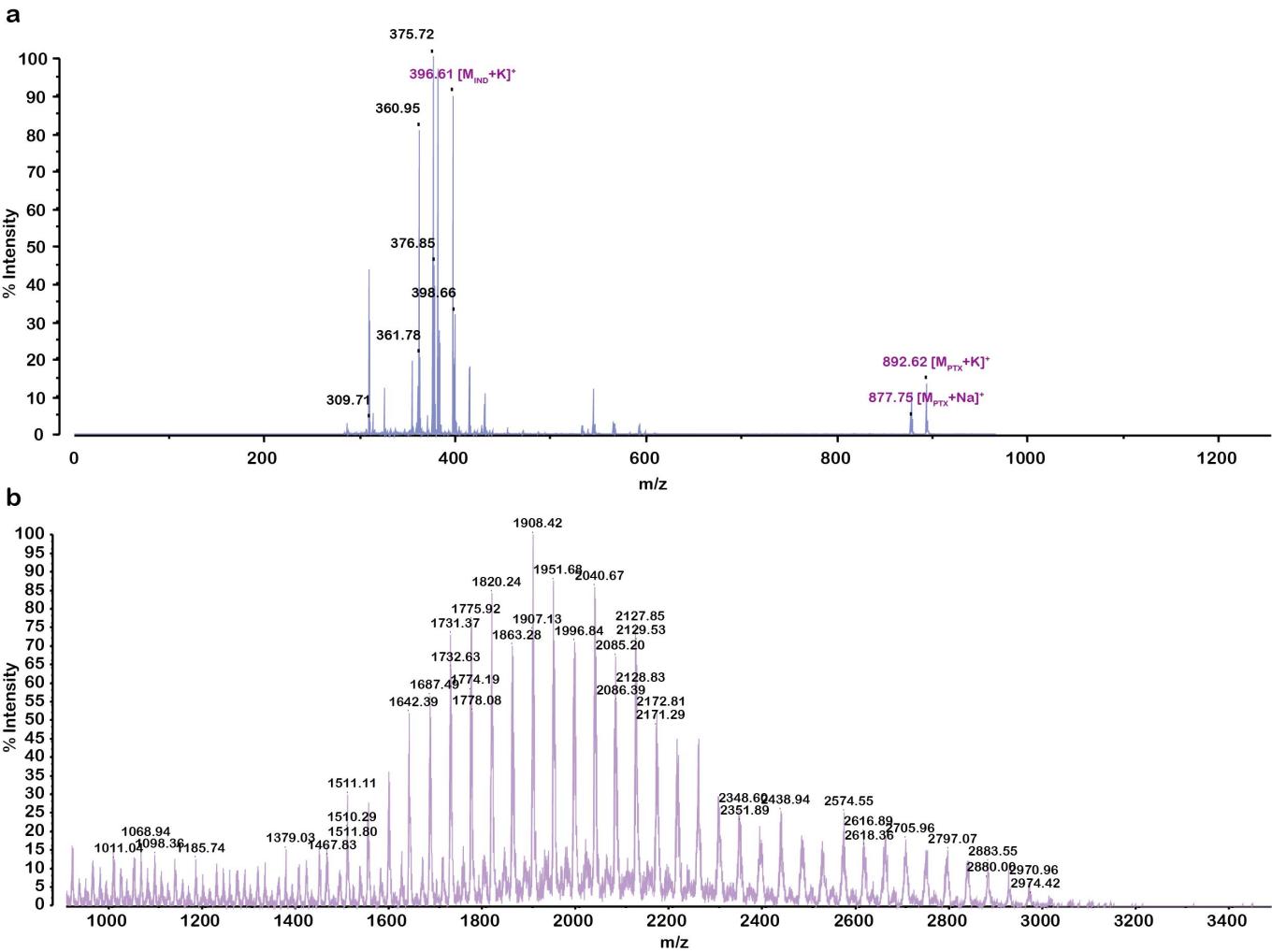


**Figure S30. MALDI-TOF MS of PiPTX.** a. Mass spectrometry within a molecular weight range of 0 - 1200 m/z. **b.** Mass spectrometry within a molecular weight range of 900-3500 m/z. The molecular ion peak at an m/z of 396.61 corresponds to the molecular weight of [M_IND_+K]^+^, indicating the presence of indomethacin; the molecular ion peaks at an m/z of 877.75 and 896.62 for [M_PTX_+Na]^+^, and [M_PTX_+K]^+^, respectively; and the molecular ion peaks ranging from 1400-3000 correspond to PEG.


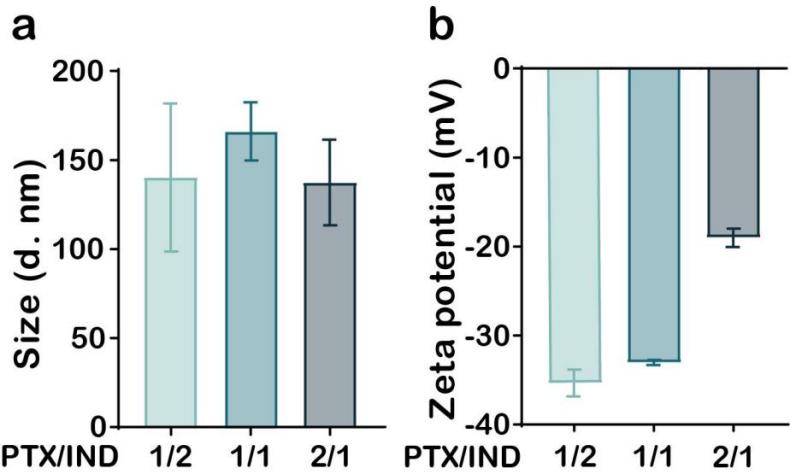


**Figure S31.** a, Size distribution and **b,** zeta potential of PiPTX at various feeding ratios. The data in the figures is presented as the Mean ± SD, n=3.


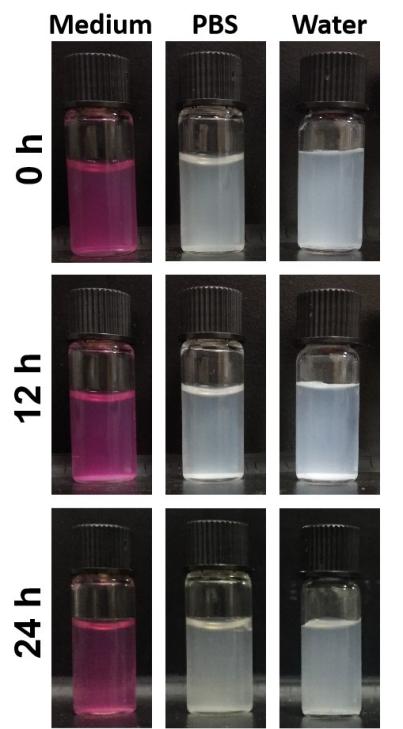


**Figure S32.** Digital photos of PiPTX dispersed in cell culture medium, PBS and water at 0 h, 12 h, or 24 h.


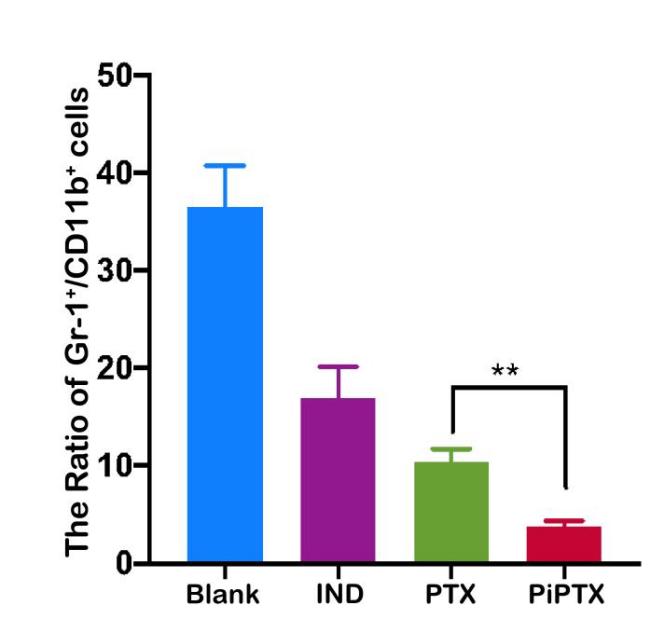


**Figure S33.** Statistical analysis of the ratios of MDSCs in polarized bone-marrow cells after treatment with IND, PTX and PiPTX. The data in the figures is presented as the Mean ± SD. n=3, One-way analysis of variance (ANOVA) test and Tukey`s multiple comparison test were conducted to analyze the Significant differences. Significant difference is indicated by ** for p < 0.01.


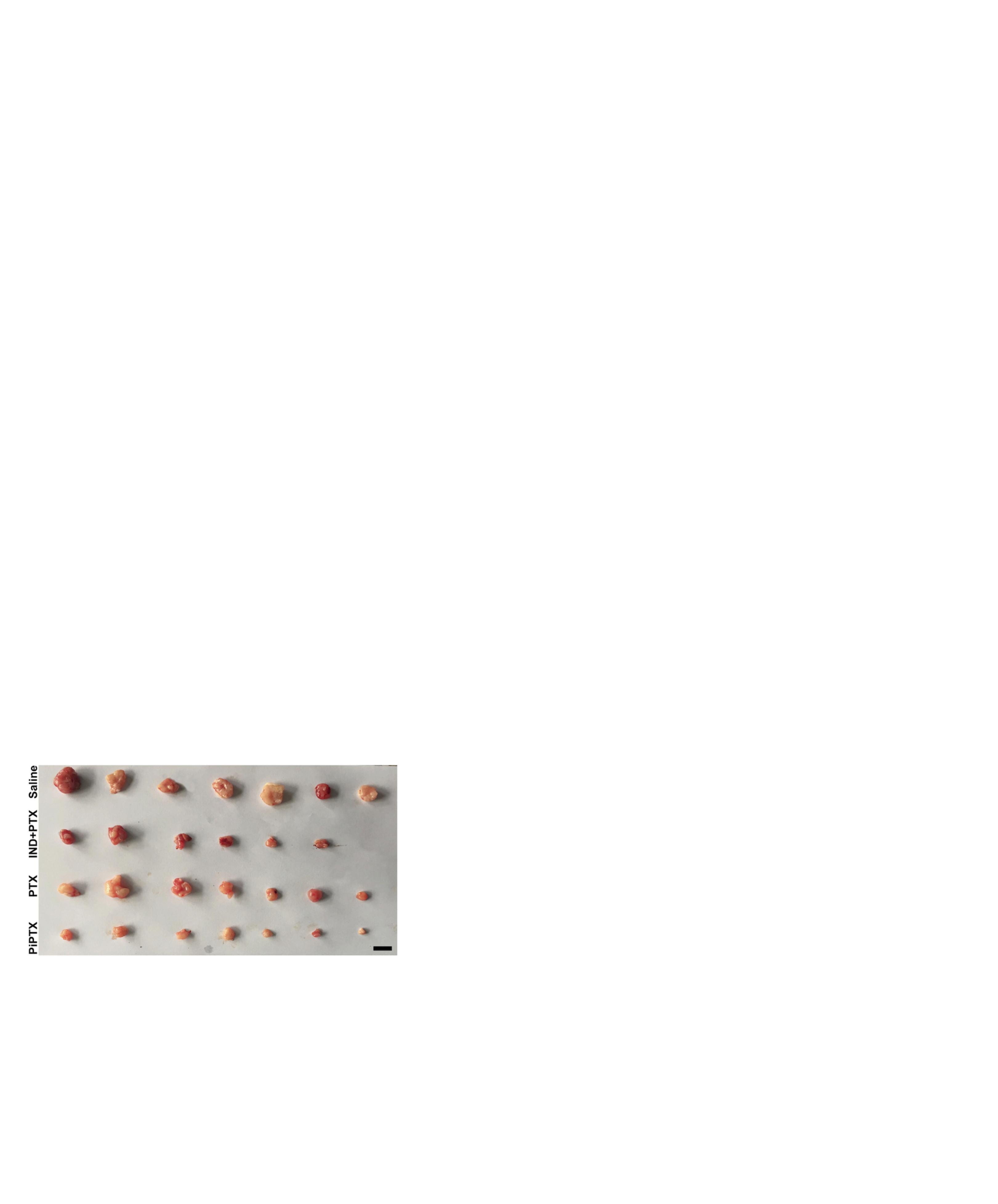


**Figure S34.** Digital photos of excised tumors after *in vivo* treatment by different dosage forms of PTX. Scale bar = 1 cm.


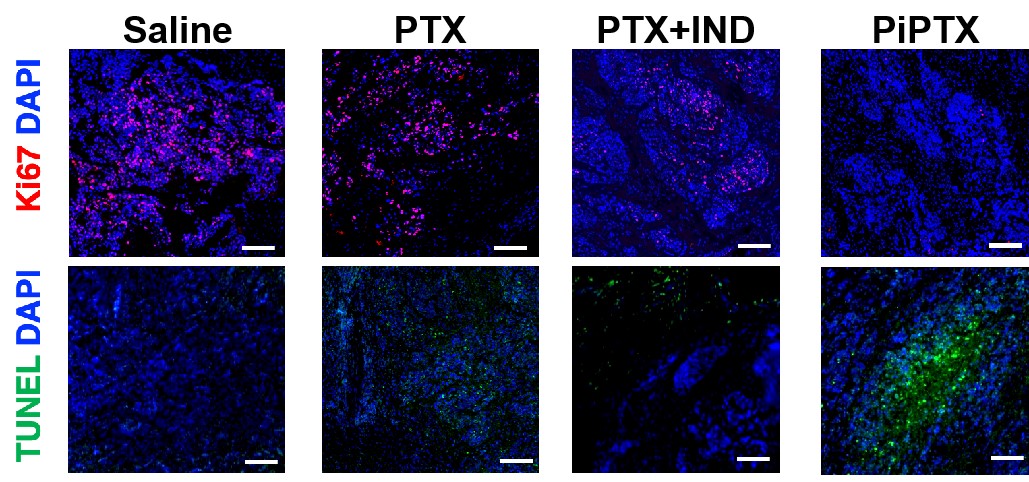


**Figure S35.** Representative images for tumor tissues after Ki67 and TUNEL fluorescent staining after treatment with saline, PTX, a mixture of PTX and IND (PTX+IND), or PiPTX. Magnification 100×, Scale bar = 100 μm.


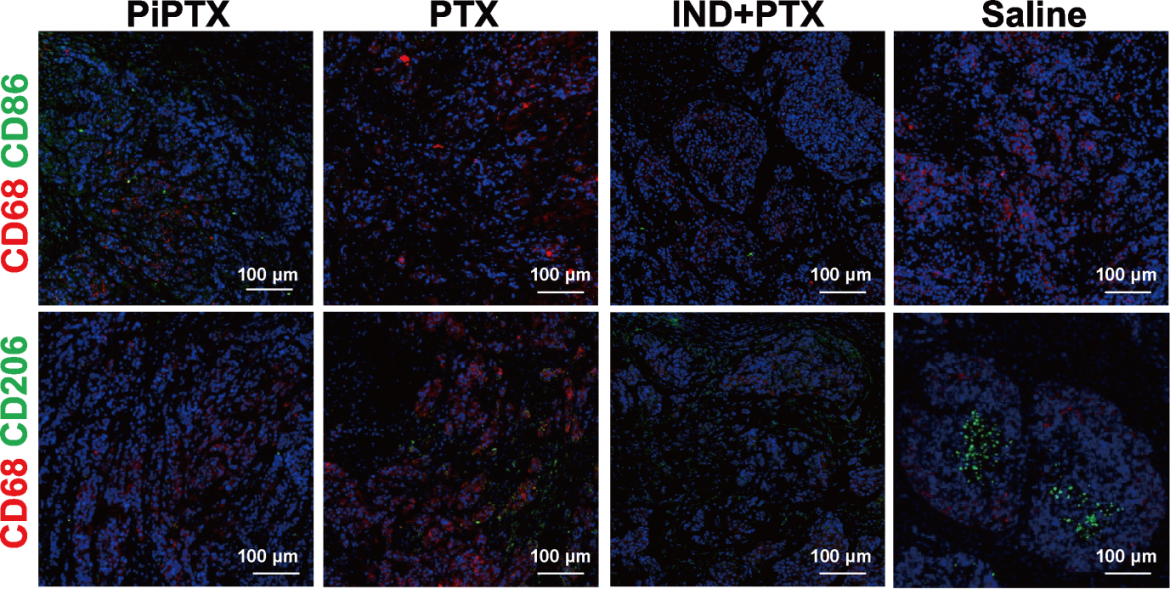


**Figure S36.** Immunostaining images of tumor sections labeled with APC-CD68+FITC-CD86 or APC-CD68+FITC-CD206. CD68 is labeled with an APC-CD68 antibody (red), CD206 or CD86 with an FITC-CD206 antibody (green) or an FITC-CD86 antibody (green), the nuclei with DAPI (blue). Magnification 100×, Scale bar = 100 μm.


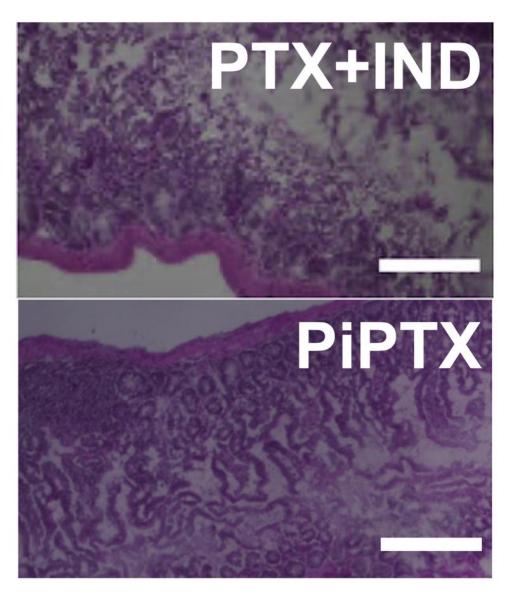


**Figure S37.** H&E staining images of the small intestine after treatment of PiPTX or a mixture of PTX and IND (PTX+IND). PiPTX could alleviate the toxicity of IND to the small intestine. Magnification 200×, Scale bar = 100 μm. Images are a representative of three independent experiments (n=3).


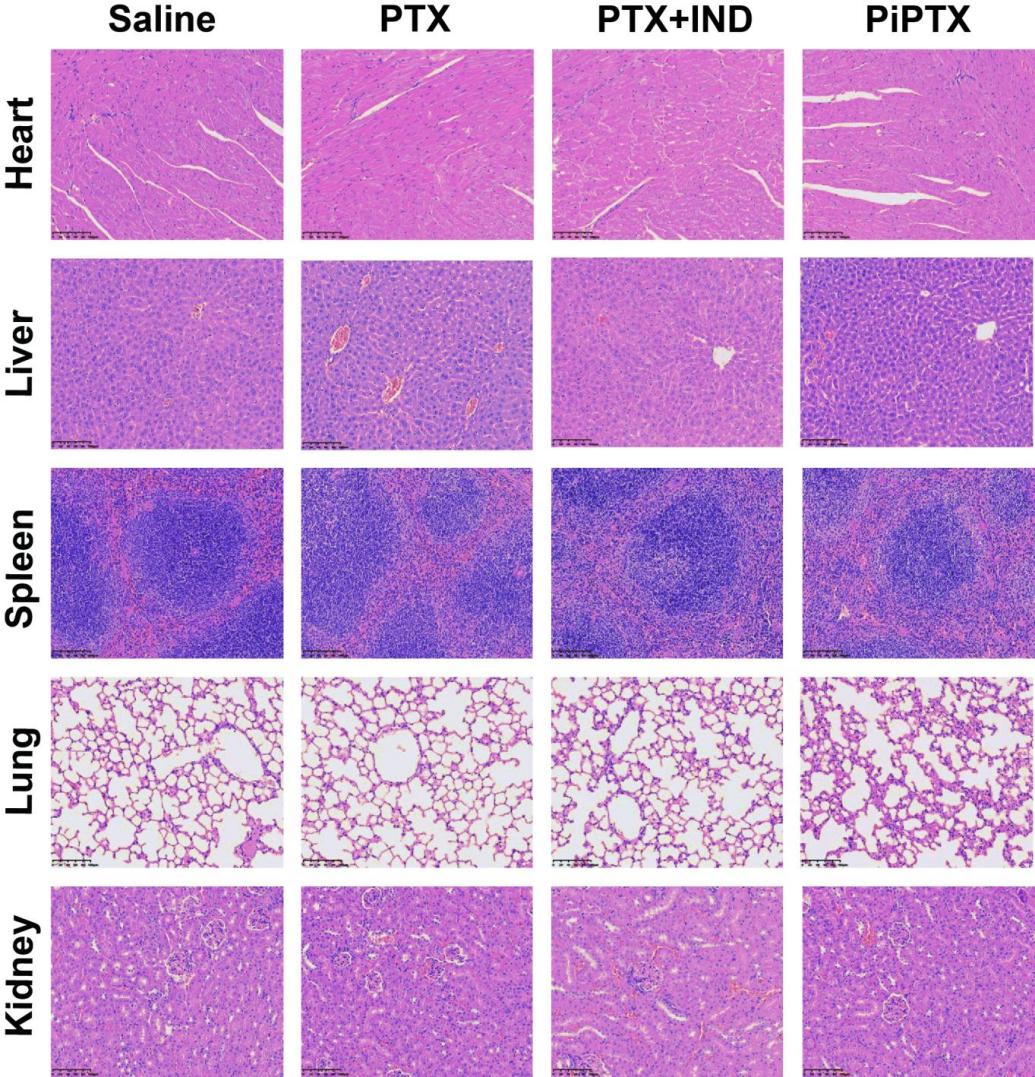


**Figure S38.** H&E staining images of main organs after treatment with saline, PTX, a mixture of PTX and IND (PTX+IND) or PiPTX. Magnification 200×, Scale bar = 100 μm.


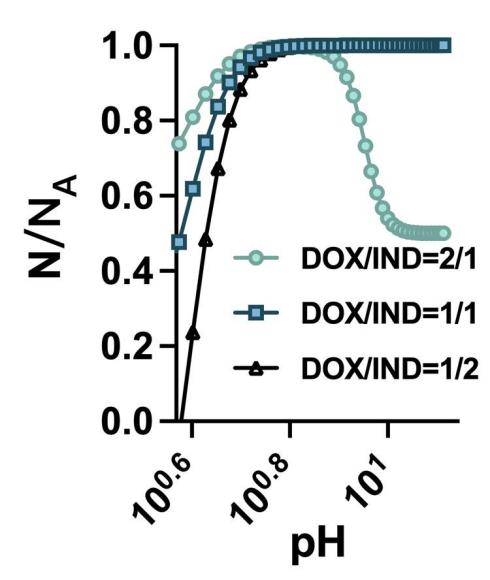


**Figure S39.** The ionization level of iDOX at various feeding ratios under different pH conditions. N is the number of particles and N_A_ is the Avogadro constant.

**Figure S40.** Cytotoxicity of different dosage forms of DOX against the HepG-2 cell line. The data in the figures is presented as the Mean ± SD, n=4.


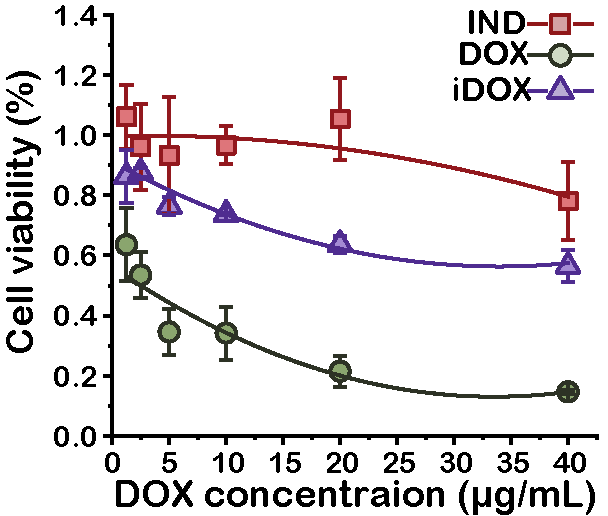


**Figure S41.** Cytotoxicity against the A549 cell line after incubation with IND, DOX or iDOX, The data in the figures is presented as the Mean ± SD, n=4.


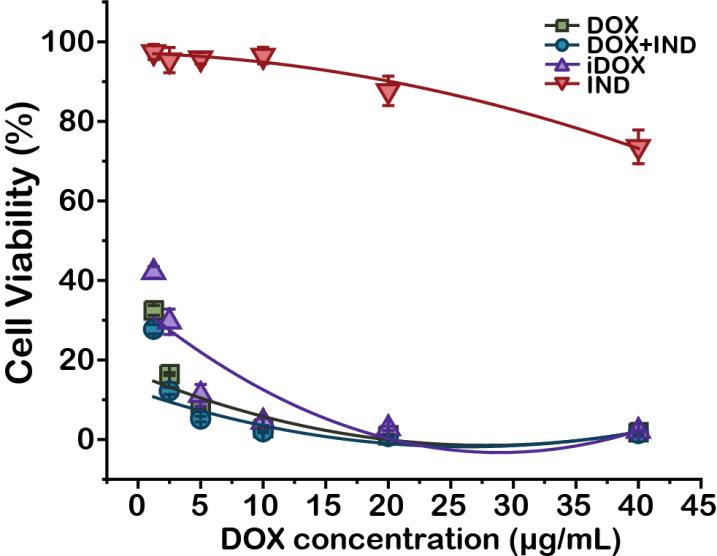


**Figure S42.** Cytotoxicity of various DOX dosage forms at different DOX concentrations against the 4T1 cell line. The data in the figures is presented as the Mean ± SD, n=5.


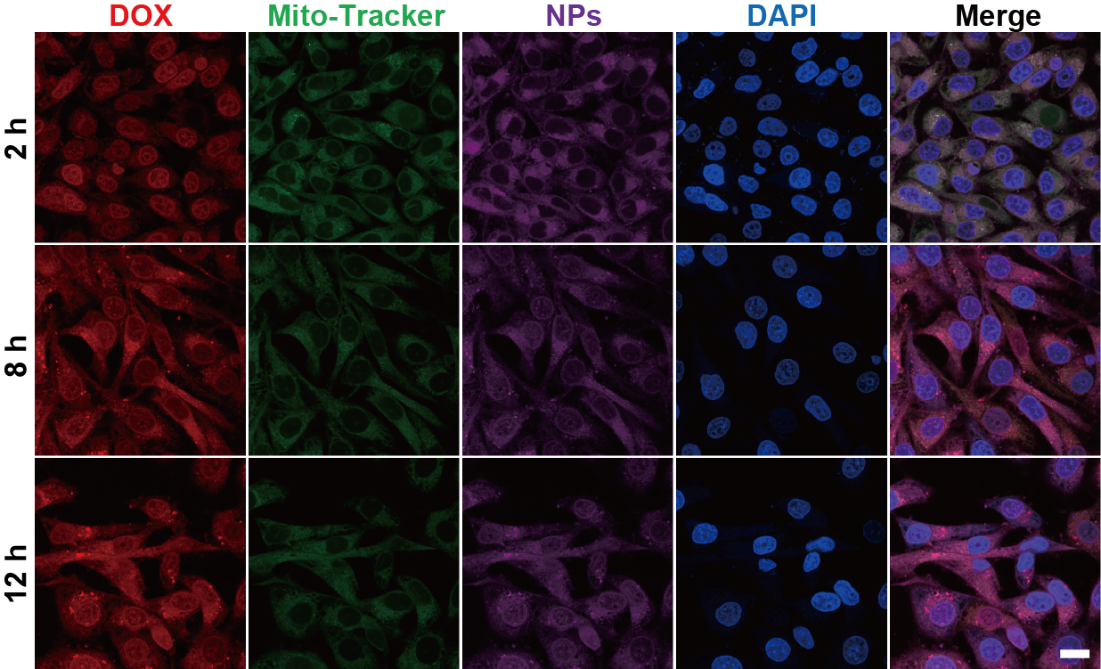


**Figure S43.** Representative images for phagocytosis of iDOX by HepG-2 cells at different time points under a confocal laser scanning microscope (CLSM). Distribution of DOX is observed under the condition of Ex = 490 nm and Em = 550 nm (red), while distribution of iDOX under the condition of Ex = 370 nm and Em = 550 nm (purple). Mitochondria are stained with Mito-Tracker Green (green) and nuclei with DAPI (blue). Magnification 600×, Scale bar = 5 μm.


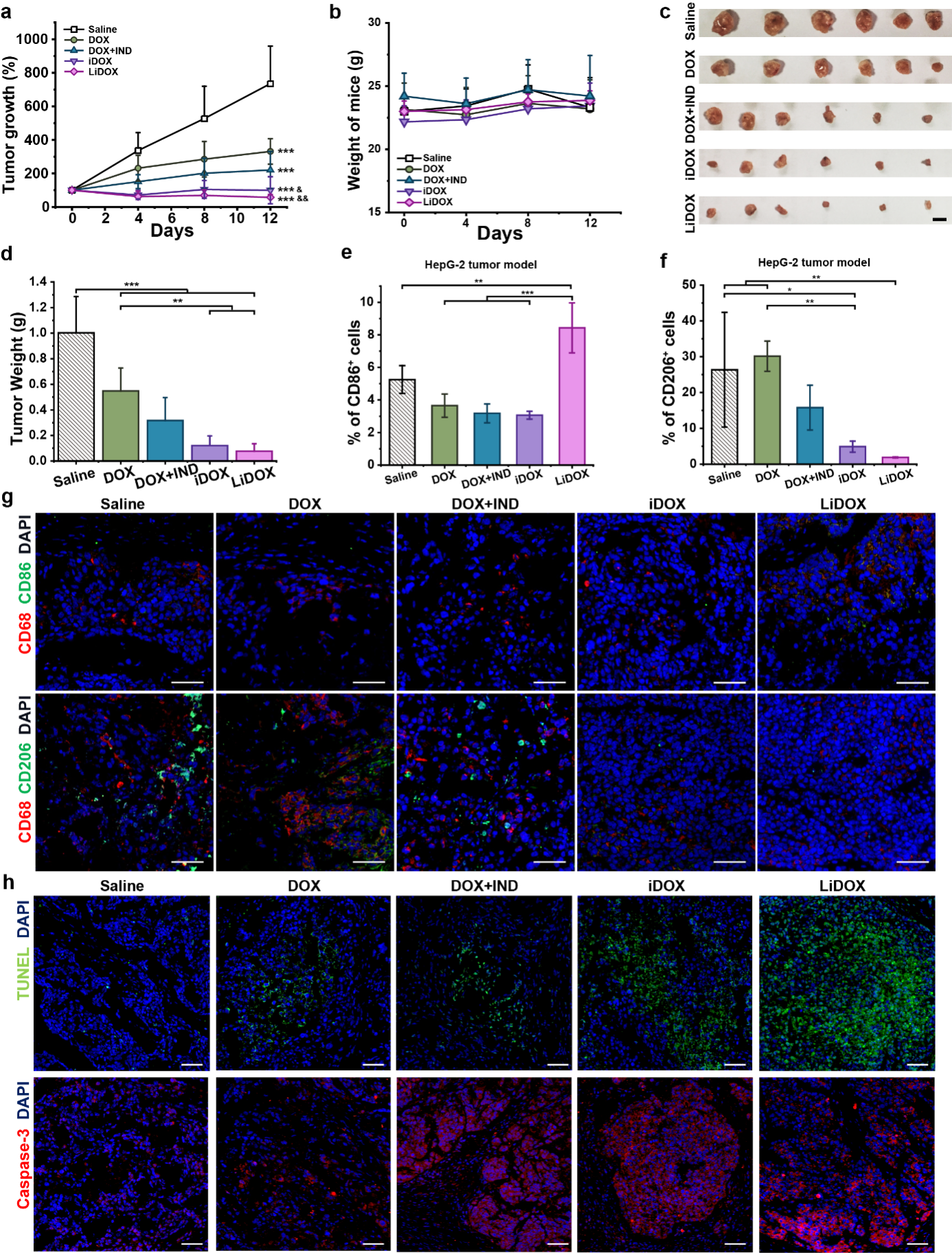


**Figure S44.** **Anti-tumor effects of different DOX dosage forms in a HepG-2 tumor model.** a) Change curves of the tumor volume and b) body weights of the mice during the treatment course; c) Digital photos and d) weights of resected tumors after treatment. Scale bar = 1 cm, n=6.e-f) Quantitative ratios of CD86^+^ and CD206^+^ immune cells in the tumor tissue, respectively, n=3; g) Representative images of CD86^+^ and CD206^+^ immune cells distributed in the tumor tissue under a fluorescence microscope. CD68 is labeled with an APC-CD68 antibody (red), CD206 or CD86 with an FITC-CD206 antibody (green) or an FITC-CD86 antibody (green), and the nuclei with DAPI (blue). Magnification 200×, Scale bar = 50 μm; h) Fluorescence images for tumor tissues after staining caspase-3 and TUNEL. Caspase-3 is labelled in red, TUNEL in green, and DAPI in blue. Magnification 100×, Scale bar = 100 μm. The data in the figures is presented as the Mean ± SD. One-way analysis of variance (ANOVA) test and Tukey`s multiple comparison test were conducted to analyze the Significant differences. Significant differences are indicated as * for p < 0.05, ** for p < 0.01 and *** for p < 0.001. Significant differences in a. are indicated as * for p < 0.05, ** for p < 0.01 and *** for p < 0.001 compare to Saline, & for p < 0.05, && for p < 0.01 compare to DOX.


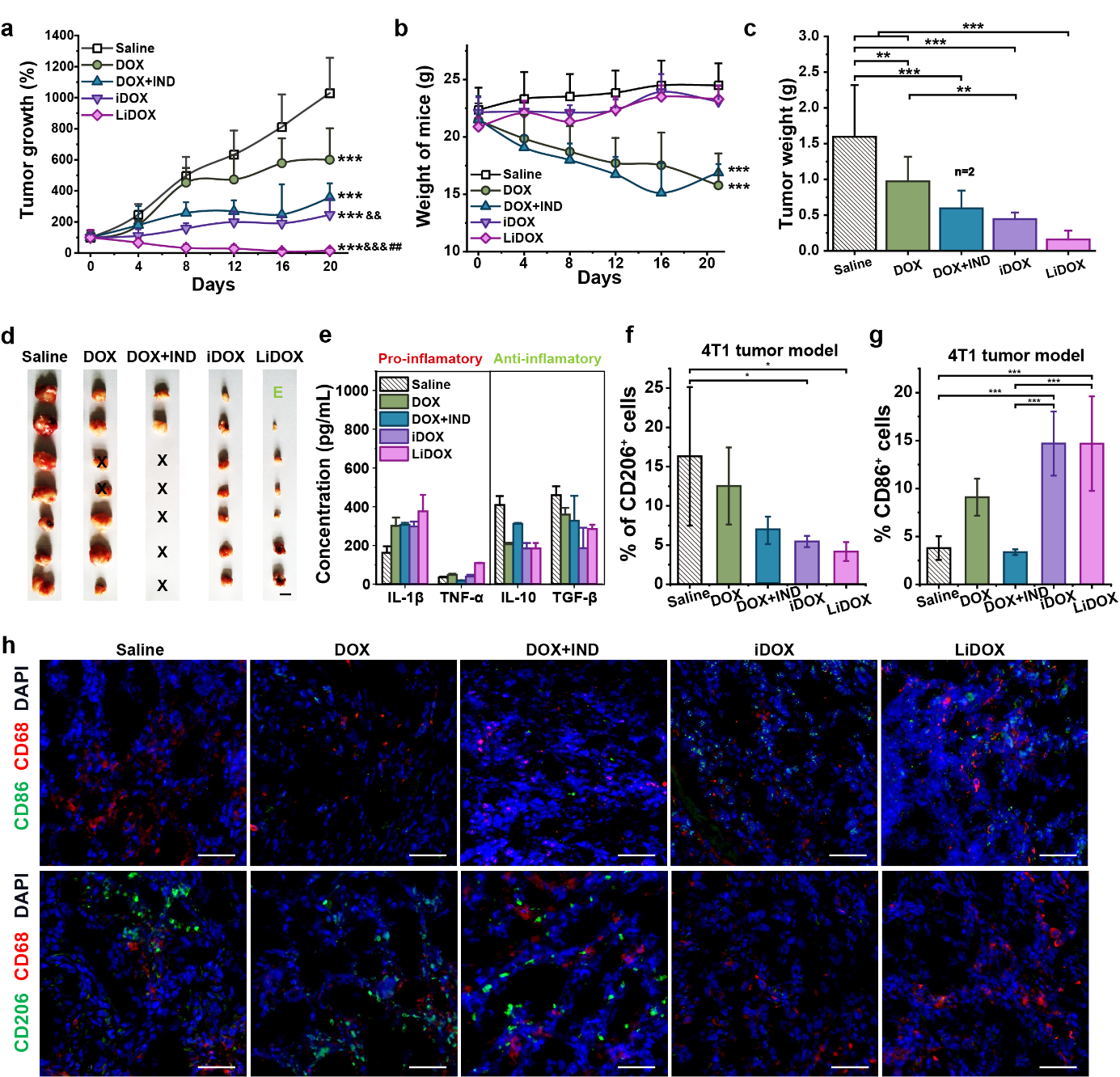


**Figure S45. Anti-tumor effects of different DOX dosage forms in a 4T1 tumor model.** a-b) Change curves of the tumor volume, and the body weight of the mice during the treatment course, respectively, n=7; c-d) Weights and digital photos of excised tumors after treatment, respectively. n=7, Scale bar = 1 cm; e) The concentration of anti-tumoral cytokines (IL-1β and TNF-α) and pro-tumoral cytokines (IL-10 and TGF-β) in the tumor tissue after treatment, n=3 f-g) Quantitative ratios of CD206^+^ and CD86^+^ immune cells within the tumor tissue, respectively, n=3; h) Representative images of CD86^+^ and CD206^+^ immune cells distributed in the tumor tissue under a fluorescence microscope. CD68 is labeled with an APC-CD68 antibody (red), CD206 or CD86 with an FITC-CD206 antibody (green) or an FITC-CD86 antibody (green), and the nuclei with DAPI (blue). Magnification 200×, Scale bar = 50 μm. The data in the figures are presented as the Mean ± SD. One-way analysis of variance (ANOVA) test and Tukey`s multiple comparison test were conducted to analyze the Significant differences. Significant differences are indicated as * for p < 0.05, ** for p < 0.01 and *** for p < 0.001. Significant differences in a. are indicated as *** for p < 0.001 compare to Saline, && for p < 0.01, &&& for p < 0.001 compare to DOX, $$ for p< 0.01 compare to DOX+IND.


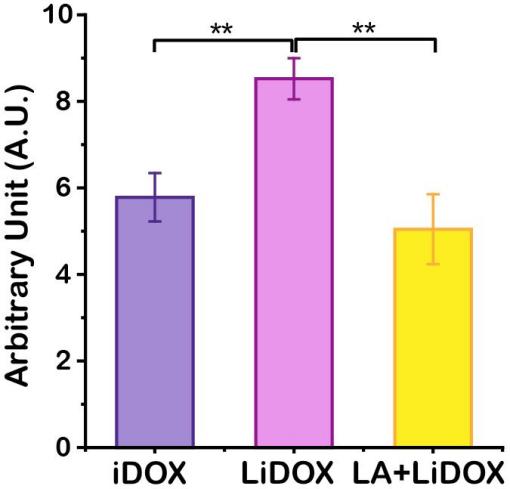


**Figure S46.** Statistical analysis of the fluorescence intensity of DOX in the confocal microscope images of the tumor tissue after treatment with iDOX, LiDOX, or LA+LiDOX (in a sequential order of LA and LiDOX). The data in the figures is presented as the Mean ± SD, n=3, One-way analysis of variance (ANOVA) test and Tukey`s multiple comparison test were conducted to analyze the Significant differences. Significant differences are indicated as ** for p < 0.01.


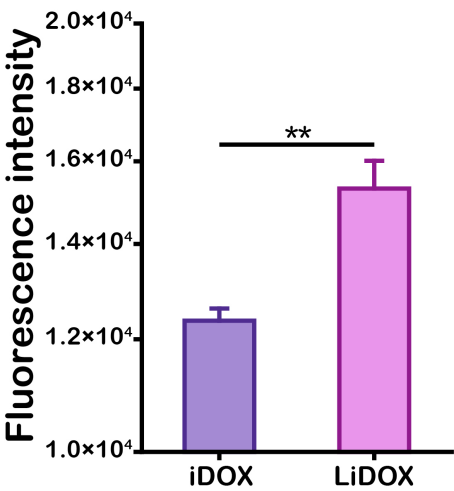


**Figure S47.** Statistical analysis of the fluorescence intensity of DOX in Raw 264.7 cells after treatment with iDOX or LiDOX for 12 h. The data in the figures is presented as the Mean ± SD, n=3, Significant difference analysis was conducted using t-test. Significant difference is indicated as *** for p < 0.001.


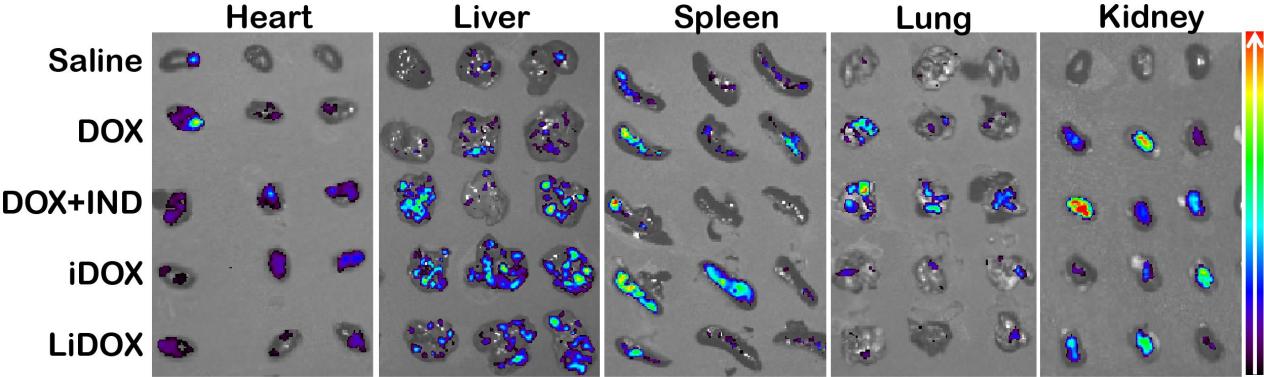


**Figure S48.** *Ex vivo* images of main organs harvested after treatment with saline, DOX, a mixture of DOX and IND (DOX+IND), iDOX or LiDOX.

**Figure S49.** Co-localization of the fluorescence signal of DOX and CD68 in the tumor after intravenous administration of LiDOX.


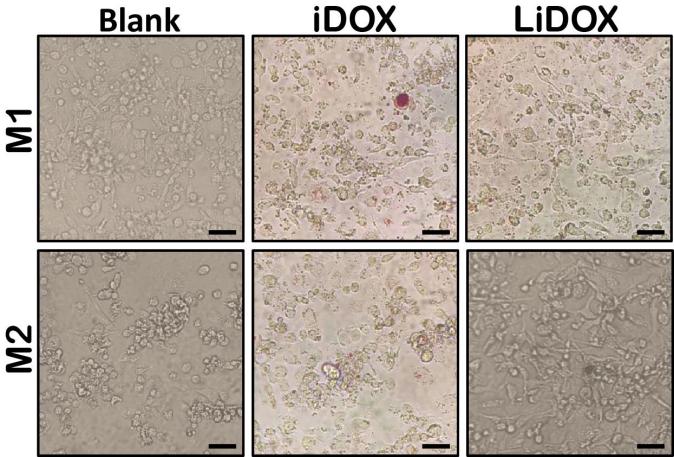


**Figure S50**. Morphological photographs of macrophage phenotypes under a light microscope after co-incubation with iDOX or LiDOX. Magnification 200×, Scale bar = 20 μm.


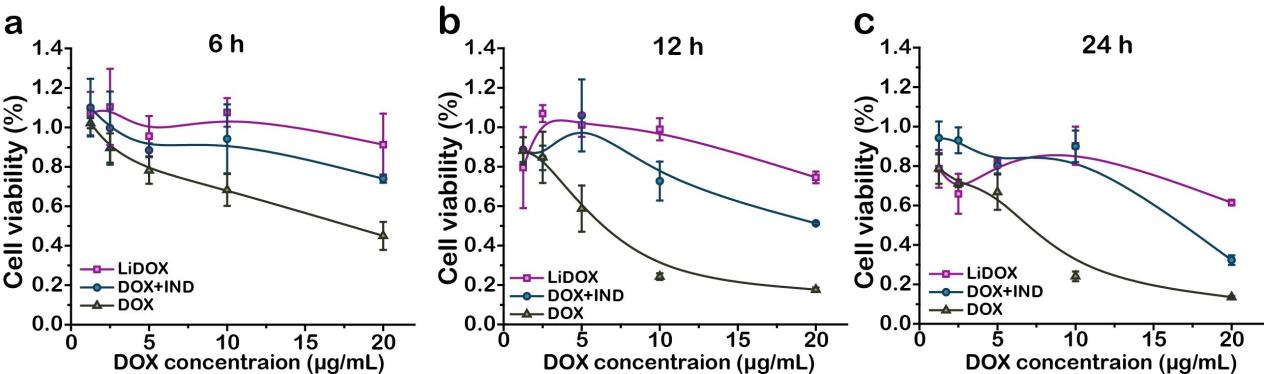


**Figure S51.** Cytotoxicity of different DOX dosage forms against the Raw 264.7 cell line after different incubation durations. The data in the figures is presented as the Mean ± SD, n=4.

**Figure S52.** Survival rates of the mice after administration of different dosage forms of DOX. n=7.


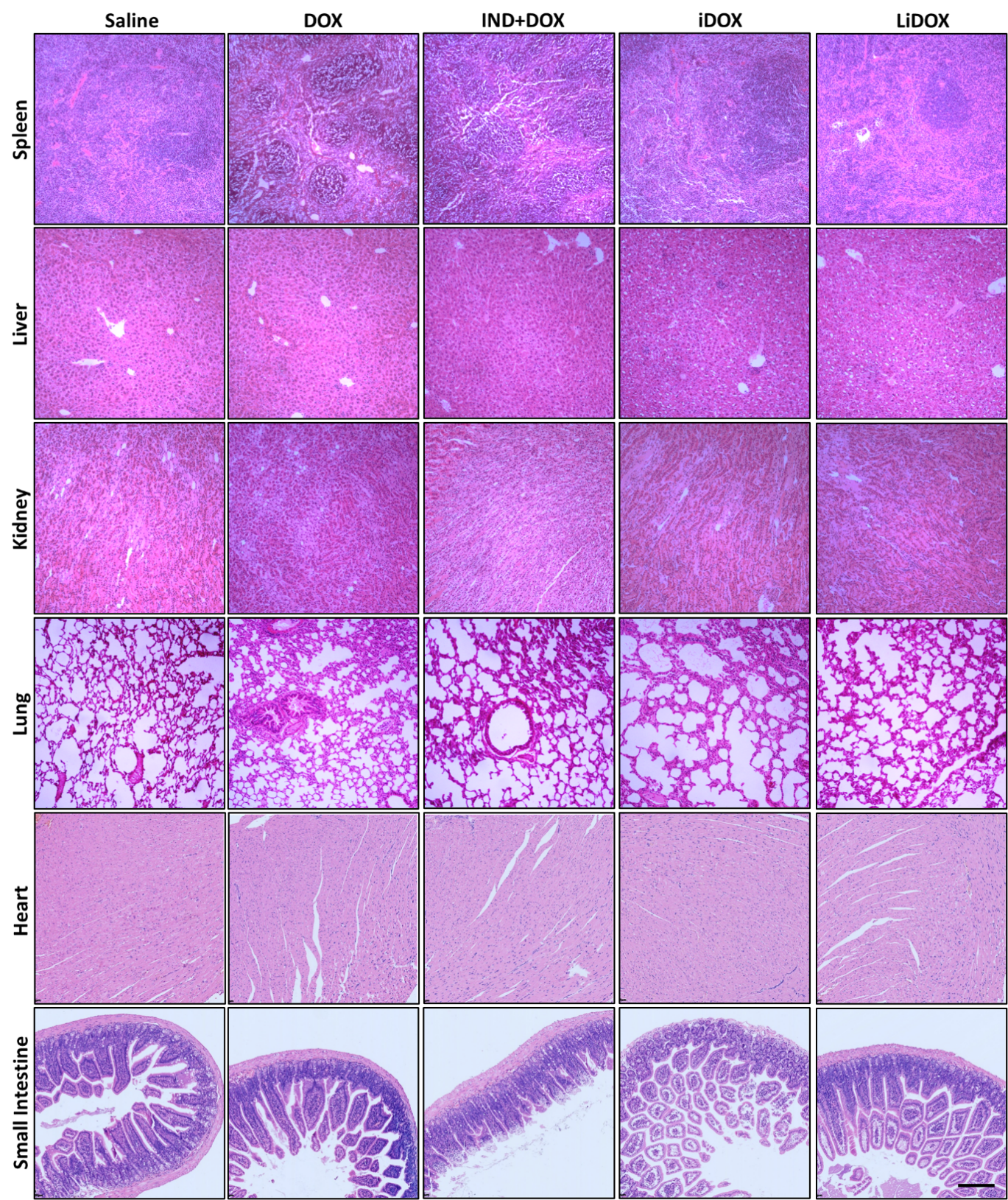


**Figure S53.** H&E staining images of main organs after treatment with saline, DOX, DOX+IND, iDOX or LiDOX for 20 days in a 4T1 tumor model. Magnification 100×, Scale bar = 200 μm.

# Supplementary Tables

**Table S1:** Conditions setting for antineoplastic drugs screening on DrugBank.

| **Conditions** | **Status** |
| --- | --- |
| Approved | √ |
| Category | Antineoplastic Agents |
| Small molecule | √ |
| Predicted logp | > 0 |
| Dosage | Intravenous |
| Products | is present |
| Withdraw | × |

**Table S2:** Properties of antineoplastic drugs.

| **Drug name** | **melting point (°C)** | **water solubility** | **logP** | **logS** | **pKa** | **pKa (Strongest Acidic)** | **pKa (Strongest Basic)** | **Physiological Charge** | **Hydrogen Acceptor Count** | **Hydrogen Donor Count** | **Polar Surface Area** | **Polarizability** | **Number of Rings** |
| --- | --- | --- | --- | --- | --- | --- | --- | --- | --- | --- | --- | --- | --- |
| **Amsacrine** | 235 | <1 mg/mL | 4.66 | -5.1 | - | 10.82 | 8.44 | 1 | 5 | 2 | 80.32 Å^2^ | 41.65 Å^3^ | 4 |
| **Azathioprine** | 243.5 | Insoluble | 0.10 | -2.4 | 7.87 (at 25 °C) | 8.62 | 2.16 | 0 | 6 | 1 | 115.42 Å^2^ | 24.37 Å^3^ | 3 |
| **Belinostat** | - | 0.14 mg/mL | 1.83 | -4 | 7.87 and 8.71 by potentiometry | 7.82 | -5.1 | 0 | 4 | 3 | 95.5 Å^2^ | 30.99 Å^3^ | 2 |
| **Bendamustine** | - | 0.0618 mg/mL | 3.07 | -3.8 | - | 4.36 | 6.41 | -1 | 4 | 1 | 58.36 Å^2^ | 38.19 Å^3^ | 2 |
| **Bortezomib** | 139-143 | 0.0532 mg/mL | 0.89 | -3.9 | - | 8.64 | -0.59 | 0 | 6 | 4 | 124.44 Å^2^ | 40.65 Å^3^ | 2 |
| **Cabazitaxel** | - | 0.00413 mg/mL | 3.69 | -5.3 | - | 11.96 | -3.6 | 0 | 10 | 3 | 202.45 Å^2^ | 86.25 Å^3^ | 6 |
| **Carfilzomib** | - | Insoluble | 3.21 | -5.2 | 3.5 | 11.91 | 4.96 | 0 | 8 | 4 | 158.47 Å^2^ | 79.45 Å^3^ | 4 |
| **Carmustine** | 31 | 4000 mg/L (at 25 °C) | 1.24 | -2.2 | - | 13.36 | -5.3 | 0 | 2 | 1 | 61.77 Å^2^ | 18.8 Å^3^ | 0 |
| **Cyclophosphamide** | 48-49 | 15.1 mg/mL | 0.76 | -1.2 | - | 13.48 | - | 0 | 1 | 1 | 41.57 Å^2^ | 23.72 Å^3^ | 1 |
| **Daunorubicin** | 208-209 | 30000 mg/L at 25 °C | 1.83 | -2.9 | 7.85 | 8.01 | 10.03 | 1 | 11 | 5 | 185.84 Å^2^ | 53.7 Å^3^ | 5 |
| **Diethylstilbestrol** | 1-72 | 12 mg/L (at 25 °C) | 5.07 | -4.35 | - | 8.63 | -6.5 | 0 | 2 | 2 | 40.46 Å^2^ | 30.69 Å^3^ | 2 |
| **Doxorubicin** | 229-231 | 1.18 mg/mL | 1.27 | -2.7 | - | 8.01 | 10.03 | 1 | 12 | 6 | 206.07 Å^2^ | 54.62 Å^3^ | 5 |
| **Epirubicin** | 344.53 | 1.18 mg/mL | 1.41 | -2.7 | - | 8.01 | 10.03 | 1 | 12 | 6 | 206.07 Å^2^ | 54.62 Å^3^ | 5 |
| **Etoposide** | 236-251 | 0.978 mg/mL | 0.73 | -2.8 | - | 9.33 | -3.7 | 0 | 12 | 3 | 160.83 Å^2^ | 57.95 Å^3^ | 7 |
| **Idarubicin** | - | 0.772 mg/mL | 1.69 | -2.8 | - | 8.04 | 10.04 | 1 | 10 | 5 | 176.61 Å^2^ | 50.84 Å^3^ | 5 |
| **Ifosfamide** | 39-41 | 15.0 mg/mL | 0.57 | -1.2 | - | 14.64 | - | 0 | 1 | 1 | 41.57 Å^2^ | 23.94 Å^3^ | 1 |
| **Irinotecan** | 222-223 | 0.107 mg/mL | 3.94 | -3.7 | - | 11.71 | 9.47 | 1 | 6 | 1 | 112.51 Å^2^ | 65.27 Å^3^ | 7 |
| **Melphalan** | 182.5 | < 0.1 g/100 mL at 22 °C | -0.52 | - | - | 1.29 | 9.51 | 0 | 4 | 2 | 66.56 Å^2^ | 31.38 Å^3^ | 1 |
| **Methylprednisolone** | 232.5 | 120 mg/L (at 25 °C) | 1.525 | -2.99 | - | 12.59 | -2.9 | 0 | 5 | 3 | 94.83 Å^2^ | 40.77 Å^3^ | 4 |
| **Mitoxantrone** | - | 0.734 mg/mL | -3.1 | -2.8 | - | 8.27 | 9.36 | 2 | 10 | 8 | 163.18 Å^2^ | 48.49 Å^3^ | 3 |
| **Paclitaxel** | 216-217 | Insoluble | 3 | -5.2 | - | 11.9 | -1.2 | 0 | 10 | 4 | 221.29 Å^2^ | 87.15 Å^3^ | 7 |
| **Pixantrone** | - | 0.494 mg/mL | 0.57 | -2.8 | - | - | 9.52 | 2 | 7 | 4 | 123.13 Å^2^ | 35.09 Å^3^ | 3 |
| **Pralatrexate** | - | 0.0178 mg/mL | 0.1 | -4.4 | 3.25, 4.76, and 6.17 | 3.28 | 2.03 | -2 | 11 | 5 | 207.3 Å^2^ | 47.31 Å^3^ | 3 |
| **Raltitrexed** | 180-184 | 0.0181 mg/mL | 1.65 | -4.4 | - | 3.72 | 1.24 | -2 | 9 | 4 | 148.4 Å^2^ | 45.55 Å^3^ | 3 |
| **Tacrolimus** | 126 | Insoluble | 3.19 | -5.3 | - | 9.96 | -2.9 | 0 | 11 | 3 | 178.36 Å^2^ | 87.9 Å^3^ | 4 |
| **Temsirolimus** | - | 0.00235 mg/mL | 4.39 | -5.6 | - | 9.96 | -2.9 | 0 | 14 | 4 | 241.96 Å^2^ | 112.7 Å^3^ | 4 |
| **Teniposide** | 242-246 | 0.0598 mg/mL | 2.78 | -4 | - | 9.33 | -3.7 | 0 | 12 | 3 | 160.83 Å^2^ | 64.83 Å^3^ | 8 |
| **Valproic acid** | - | 1.3 mg/mL | 2.75 | -1.86 | 4.8 | 5.14 |  | -1 | 2 | 1 | 37.3 Å^2^ | 17 Å^3^ | 0 |
| **Verteporfin** | - | 0.0136 mg/mL | 2.1 | -4.7 | - | 4.18 | 4.86 | -1 | 7 | 3 | 173.56 Å^2^ | 81.21 Å^3^ | 6 |
| **Vinblastine** | 267 | 0.0169 mg/mL | 3.70 | -4.7 | - | 10.87 | 8.86 | 2 | 9 | 3 | 154.1 Å^2^ | 87.46 Å^3^ | 9 |
| **Vincristine** | 220 | 0.03 mg/mL | 2.82 | -4.4 | 5 | 10.85 | 8.66 | 2 | 9 | 3 | 171.17 Å^2^ | 88.32 Å^3^ | 9 |
| **Vinflunine** | 244 | 0.00564 mg/mL | 4.5 | -5.2 | - | 10.87 | 8.66 | 2 | 8 | 2 | 133.87 Å^2^ | 85.68 Å^3^ | 9 |
| **Vinorelbine** | 181-183 | 10 mg/mL | 4.39 | -4.8 | - | 10.87 | 8.66 | 2 | 8 | 2 | 133.87 Å^2^ | 84.31 Å^3^ | 9 |

**Table S3:** Properties of NSAIDs.

| **Drug name** | **melting point (°C)** | **water solubility** | **logP** | **logS** | **pKa** | **pKa (Strongest Acidic)** | **pKa (Strongest Basic)** | **Physiological Charge** | **Hydrogen Acceptor Count** | **Hydrogen Donor Count** | **Polar Surface Area** | **Polarizability** | **Number of Rings** |
| --- | --- | --- | --- | --- | --- | --- | --- | --- | --- | --- | --- | --- | --- |
| **Indomethacin** | 151 | 0.937 mg/L (at 25 °C) | 4.27 | -4.62 | 4.5 | 3.79 | -2.9 | -1 | 4 | 1 | 68.53 Å^2^ | 36.64 Å^3^ | 3 |
| **Sulindac** | 183 | 3000 mg/L | 3.42 | -4.2 | 4.7 | 4.09 | -8.1 | -1 | 3 | 1 | 54.37 Å^2^ | 37.21 Å^3^ | 3 |
| **Ketoprofen** | 94 | 51 mg/L (at 22 °C) | 3.12 | -3.7 | 4.45 | 3.88 | -7.5 | -1 | 3 | 1 | 54.37 Å^2^ | 26.56 Å^3^ | 2 |
| **Aceclofenac** | 149-153 | Insoluble | 2.170 | -5.2 | - | 3.44 | -2.1 | -1 | 4 | 2 | 75.63 Å^2^ | 32.76 Å^3^ | 2 |
| **Aspirin** | 138-140 | 10 mg/mL | 1.18 |  | 3.5 | 3.41 | -7.1 | -1 | 3 | 1 | 63.6 Å^2^ | 17.1 Å^3^ | 1 |
| **Celecoxib** | 157-159 | Poorly soluble | 3.53 | -4.9 | 11.1 | 10.6 | -0.41 | 0 | 3 | 1 | 77.98 Å^2^ | 35.2 Å^3^ | 3 |
| **Diclofenac** | 283-285 °C | 2.37 mg/L (at 25 °C) | 4.51 | -4.8 | 4.15 | 4 | -2.1 | -1 | 3 | 2 | 49.33 Å^2^ | 27.93 Å^3^ | 2 |
| **Etodolac** | 146.5 | 16 mg/L | 2.5 | -3.9 | 4.65 | 4.73 | -4.2 | -1 | 3 | 2 | 62.32 Å^2^ | 31.94 Å^3^ | 3 |
| **Fenoprofen** | 168-171 | 0.0811 mg/mL | 3.87 | -3.5 | 4.5 | 3.96 | -3.7 | -1 | 2 | 1 | 46.53 Å^2^ | 25.3 Å^3^ | 2 |
| **Flufenamic acid** | 133.5 | 9.09 mg/L (at 25 °C) | 5.25 | -4.6 | - | 3.88 | -2.1 | -1 | 3 | 2 | 49.33 Å^2^ | 24.67 Å^3^ | 2 |
| **Ketorolac** | 162-165 (tromethamine salt) | 0.513 mg/mL | 2.66 | -2.7 | - | 3.84 | -7.8 | -1 | 3 | 1 | 59.3 Å^2^ | 26.67 Å^3^ | 3 |
| **Lornoxicam** | - | 0.0437 mg/mL | 3.08 | -3.9 | - | 1.82 | 4.22 | -1 | 5 | 2 | 99.6 Å^2^ | 33.3 Å^3^ | 3 |
| **Mefenamic** | 230-231 | 0.0137 mg/mL | 4.58 | -4.2 | 4.2 | 3.89 | -1.6 | -1 | 3 | 2 | 49.33 Å^2^ | 26.22 Å^3^ | 2 |
| **Meloxicam** | 256 | 22 mg/ml | 2.28 | -3.4 | 4.08 | 4.47 | 0.47 | -1 | 5 | 2 | 99.6 Å^2^ | 34.25 Å^3^ | 3 |
| **Naproxen** | 152 | 15.9 mg/L (at 25 °C) |  | -4.16 | 4.15 | 4.19 | -4.8 | -1 | 3 | 1 | 46.53 Å^2^ | 24.81 Å^3^ | 2 |
| **Nimesulide** | 143-144.5 | 0.0182 mg/mL | 2.56 | -4.2 | - | 6.7 | -3.7 | -1 | 4 | 1 | 98.54 Å^2^ | 28.93 Å^3^ | 2 |
| **Piroxicam** | 198-200 | 23 mg/L (at 22 °C) | 3.06 | -4.16 | 6.3 | 4.76 | 3.79 | -1 | 5 | 2 | 99.6 Å^2^ | 32.27 Å^3^ | 3 |
| **Salicylic acid** | 158 | 2240 mg/L (at 25 °C) | 2.26 | -1.82 | 2.97 | 2.79 | -6.3 | -1 | 3 | 2 | 57.53 Å^2^ | 12.81 Å^3^ | 1 |
| **Tenoxicam** | 211 dec °C | 0.257 mg/mL | 2.42 | -3.1 | - | 2.21 | 4.26 | -1 | 5 | 2 | 99.6 Å^2^ | 31.96 Å^3^ | 3 |
| **Tolfenamic acid** | 207 | 1 mg/ml | 5.17 | -4.2 | 5.11 | 3.88 | -2.1 | -1 | 3 | 2 | 49.33 Å^2^ | 26.46 Å^3^ | 2 |
| **Ursodeoxycholic acid** | 203 | 20 mg/L (at 20 °C) | 3.00 | -4.3 | - | 4.6 | -0.54 | -1 | 4 | 3 | 77.76 Å^2^ | 46.42 Å^3^ | 4 |

**Table S4:** Properties of drugs reported capable of forming nano-assemblies.

| **Drug name** | **melting point (°C)** | **water solubility** | **logP** | **logS** | **pKa** | **pKa (Strongest Acidic)** | **pKa (Strongest Basic)** | **Physiological Charge** | **Hydrogen Acceptor Count** | **Hydrogen Donor Count** | **Polar Surface Area** | **Polarizability** | **Number of Rings** |
| --- | --- | --- | --- | --- | --- | --- | --- | --- | --- | --- | --- | --- | --- |
| **Ursolic acid** | - | 0.00059 mg/mL | 6.35 | -5.9 | - | 4.74 | -0.84 | -1 | 3 | 2 | 57.53 Å^2^ | 54.51 Å^3^ | 5 |
| **Hydroxycamptothecin** | - | 0.331 mg/mL | 1.69 | -3 | - | 9.65 | 3.17 | 0 | 5 | 2 | 99.96 Å^2^ | 37.51 Å3 | 5 |
| **Irinotecan** | 222-223 | 0.107 mg/mL | 3.94 | -3.7 | - | 11.71 | 9.47 | 1 | 6 | 1 | 112.51 Å^2^ | 65.27 Å^3^ | 7 |
| **Topotecan** | 213-218 | 0.861 mg/mL | 1.84 | -2.7 | - | 8 | 9.75 | 1 | 6 | 2 | 103.2 Å^2^ | 44.86 Å^3^ | 5 |
| **Indocyanine green** | - | - | 6.05 | - | - | -0.94 | 4.85 | 0 | 7 | 0 | 120.65 Å^2^ | 86.08 Å^3^ | 6 |
| **Porphyrin** | - | 0.00297 mg/mL | 5.16 | -5.2 | - | 14.67 | 5.23 | 0 | 4 | 0 | 51.56 Å^2^ | 34.1 Å^3^ | 5 |
| **Isothiocyanate** | 136-139 | Insoluble in water | 3.15 | -6.02 | - | - | - | 0 | 2 | 0 | 43.9 Å² | 25.4 Å³ | 1 |
| **Curcumin** | 183 | 0.00575 mg/mL | 3.62 | -4.8 | - | 9.08 | -4.4 | 0 | 6 | 2 | 93.06 Å^2^ | 38.12 Å^3^ | 2 |
| **Geﬁtinib** | - | 0.027 mg/mL | 4.02 | -4.2 | - | 16.11 | 6.85 | 0 | 7 | 1 | 68.74 Å^2^ | 46.11 Å^3^ | - |
| **Sorafenib** | 187-226 | 0.00152 mg/mL | 4.54 | -5.5 | - | -7.2 | 15.04 | 0 | 5 | 3 | 95.84 Å² | 42.13 Å³ | 4 |
| **Vandetanib** | - | Poorly soluble | 3.9 | -5.2 | - | - | 6.6 | 0 | 5 | 2 | 78.4 Å² | 40.7 Å³ | 3 |
| **Probucol** | 124-126 | 0.0017 mg/mL | 10.5 | -6.8 | - | - | - | 0 | 2 | 2 | 40.46 Å² | 41.9 Å³ | 2 |
| **Bicalutamide** | 192-193 | 0.005 mg/mL | 2.92 | -5.2 | - | - | 12.6 | 0 | 3 | 2 | 78.0 Å² | 36.1 Å³ | 2 |
| **Azelnidipine** | - | Poorly soluble | 5.7 | -5.7 | - | - | 8.7 | 0 | 6 | 1 | 107.6 Å² | 52.9 Å³ | 3 |
| **Adenosine triphosphate** | - | Highly soluble | -3.0 | -1.1 | - | 0.9 | 6.6 | -4 | 10 | 8 | 331.8 Å² | 84.1 Å³ | 1 |

**Table S5:** Indicators for the assessment of different models

| Model | Accuracy | Recall | F1-score |
| --- | --- | --- | --- |
| SVM | 0.8108 | 0.8000 | 0.8727 |
| KNN | 0.8919 | 1.0000 | 0.9375 |
| LR | 0.9189 | 0.9667 | 0.9508 |

**Table S6:** The interaction parameters used in DPD simulations for PTX/IND (unit: kT).

| **α_ij_** | **PTX1** | **PTX2** | **IND** | **Water** |
| --- | --- | --- | --- | --- |
| **PTX1** | 78 |  |  |  |
| **PTX2** | 18.57 | 78 |  |  |
| **IND** | 97.27 | 64.42 | 78 |  |
| **Water** | 112.45 | 80.83 | 108.22 | 78 |

**Table S7:** The Flory-Huggins interaction parameters calculated by Blends for DPD simulation of PTX and IND (2/1), PTX in water or IND in water (Unit: Kcal/mol)

| **Base** | **Screen** | **Chi (298 K)** | **E_mix_ (298 K)** | **E_bb_ avg (298 K)** | **E_bs_ avg (298 K)** | **E_ss_ avg (298 K)** |
| --- | --- | --- | --- | --- | --- | --- |
| PTX1 | PTX2 | -18.29 | -10.83 | -9.01 | -10.59 | -8.41 |
| PTX1 | IND | 5.93 | 3.51 | -9.01 | -8.51 | -9.30 |
| PTX1 | water | 10.60 | 6.28 | -9.01 | -3.60 | -2.31 |
| PTX2 | IND | -4.18 | -2.48 | -8.41 | -9.30 | -9.30 |
| PTX2 | water | 0.87 | 0.52 | -8.41 | -3.94 | -2.31 |
| IND | water | 9.30 | 5.51 | -9.30 | -3.83 | -2.31 |
| IND | DOX | -1.55 | -0.92 | -9.30 | -12.00 | -14.52 |
| DOX | water | 27.22 | 16.12 | -14.52 | -4.14 | -2.31 |

**Table S8:** The Flory-Huggins interaction parameters calculated by Blends for DPD simulation of IND and DOX, IND in water or DOX in water (Unit: Kcal/mol).

| **Base** | **Screen** | **Chi (298 K)** | **E_mix_ (298 K)** | **E_bb_ avg (298 K)** | **E_bs_ avg (298 K)** | **E_ss_ avg (298 K)** |
| --- | --- | --- | --- | --- | --- | --- |
| IND | DOX | -8.18 | -4.85 | -4.13 | -7.38 | -9.24 |
| IND | Water | 8.18 | 4.84 | -4.13 | -6.36 | -10.85 |
| DOX | Water | 30.59 | 18.11 | -10.85 | -6.19 | -9.24 |

**Table S9:** The interaction parameters used in DPD simulations for DOX/IND (unit: kT).

| **α_ij_** | **DOX** | **IND** | **Water** |
| --- | --- | --- | --- |
| **DOX** | 78 |  |  |
| **IND** | 72.96 | 78 | 78 |
| **Water** | 166.48 | 108.22 |  |

**Table S10:** The adsorption energy between structures calculated by Adsorption Locater in *Materials Studio 8.0* (Unit: Kcal/mol).

| **Structures** | **Total energy** | **Adsorption energy** | **Rigid adsorption energy** | **Deformation energy** | **Forcefield** |
| --- | --- | --- | --- | --- | --- |
| DOX-SAL | -49.24 | -195.23 | -18.59 | -176.63 | COMPASSII |
| DOX-MEF | -42.73 | -331.94 | -24.48 | -307.46 | COMPASSII |
| DOX-KET | -21.40 | -326.15 | -21.80 | -304.35 | COMPASSII |
| PTX-SUL | -53.60 | -378.80 | -28.20 | -350.60 | COMPASSII |
| PTX-KET | -44.00 | -356.85 | -28.03 | -328.83 | COMPASSII |
| DOX-IND | 25.82 | -365.72 | -27.60 | -338.12 | COMPASSII |
| BEL-IND | 99.22 | -301.22 | -20.25 | -280.97 | Universal |
| BORT-IND | 26.19 | -66.79 | -18.22 | -48.57 | Universal |
| TEMS-IND | 70.08 | -169.84 | -22.40 | -147.44 | COMPASSII |
| CAB-IND | 26.73 | -100.94 | -25.49 | -75.45 | COMPASSII |
| CARM-IND | -58.23 | -89.37 | -15.18 | -74.19 | COMPASSII |
| VINCR-IND | 107.82 | -117.33 | -18.12 | -99.22 | COMPASSII |
| IFO-IND | -83.55 | -232.07 | -26.89 | -205.19 | COMPASSII |
| PTX-IND | 107.13 | -106.34 | -29.71 | -76.62 | COMPASSII |
| EPI-IND | 25.03 | -33.71 | -28.41 | -5.31 | COMPASSII |

**Table S11:** Properties of Nanomedicines Prepared by Microfluidic Technology

| **Combination of Drugs** | **Feeding Ratio** | **Effective Diameter (nm)** | **Polydispersity** | **Count Rate** | |
| --- | --- | --- | --- | --- | --- |
| **IND/DOX** | 1:1 | 266.72 | 0.201 | | 392.4 |
| **SUL/PTX** | 1:1 | 232.9 | 0.211 | | 410 |
| **KET/DOX** | 1:1 | 238.1 | 0.234 | | 433.6 |
| **SAL/DOX** | 1:1 | 208.9 | 0.164 | | 431.8 |
| **IND/TEMS** | 1:1 | 223.5 | 0.082 | | 403.8 |

**Table S12.** Comparison of carrier materials and costs between @LipoDOX and iDOX.

|  | **@LipoDOX** | | | **iDOX** | | |
| --- | --- | --- | --- | --- | --- | --- |
|  | **Materials** | **Portion (mg/mL)** | **Cost ($ per mL)** | **Materials** | **Portion (mg/mL)** | **Cost ($ per mL)** |
| **Excipients** | DSPC;  Cholesterol;  DSPE-PEG | 9.58  3.19  3.19 | 10.83  12.80  22.43 | Indomethacin | 1 | 2.23 |
| **Total ($ per mL)** | 46.06 | | | 2.23 | | |

(Data from: https://www.fda.gov/media/148964/download; https://www.accessdata.fda.gov/drugsatfda_docs/label/2021/214487s000lbl.pdf; <https://www.>chemicalbook.com/SupplierNews_59867.htm)
